# DFT Studies on Ni-Mediated C–F Cleavage for the Synthesis of Cyclopentadiene Derivatives

**DOI:** 10.3389/fchem.2018.00319

**Published:** 2018-08-13

**Authors:** Wen-Jie Chen, Ruo-Nan Xu, Weimin Lin, Xuejiao Sun, Bin Wang, Qi-Hui Wu, Xin Huang

**Affiliations:** ^1^Department of Material Chemistry, College of Chemical Engineering and Materials Science Quanzhou Normal University, Quanzhou, China; ^2^Department of Chemistry, College of Chemistry Fuzhou University, Fuzhou, China

**Keywords:** DFT studies, Ni-mediated, C-F cleavage, cyclopentadiene derivative, regioselectivity

## Abstract

Density functional theory calculations have been performed to study the detailed mechanism of Ni-mediated [3+2] cycloaddition of 2-trifluoromethyl-1-alkenes with alkynes via cleavage of two C-F bonds. It was found that the reaction pathway involves oxidative cyclization, the first *β*-fluorine elimination, and then intramolecular 5-*endo* insertion of difluoroalkene, followed by the second cleavage of C-F bond, and finally the dissociation of difluorides yields the fluorine-containing product cyclopentadienes in sequence. The overall rate-determining step is the combined processes of the β-fluorine elimination and the 5-*endo* insertion. Furthermore, we investigated the effect of different ligands and the regioselectivity of asymmetric alkynes. The detailed energy profiles and structures are presented in this study.

## Introduction

The knowledge of formation and breakage of C–C bonds is important issue in the organic synthesis reactions. However, the high strength of the C–C bond makes it difficult to achieve. Weaker metal–carbon (M–C) bonding provides the necessary fundamental basis for catalytic transformations (Macgregor et al., 2003; Ananikov et al., 2005; Ananikov, 2015). Nickel holds a special place among the transition metals on account of reactivity trends, functional group tolerance, and catalytic activity (Montgomery, 2004). Theoretical studies showed that the M–C bond strength of group 10 metals changes in the order Ni–C < Pd–C < Pt–C < C–C (Macgregor et al., 2003; Ananikov et al., 2005; Ananikov, 2015), which supports the higher reactivity of nickel species and provides a reference for the design of active catalysts. In particular, Ni-catalyzed cross-coupling of electrophiles (organic halides and pseudohalides) with carbon nucleophiles (organometallic compounds) have important consequences (Negishi, 1982; Tamao et al., 1982; Kumada, 2009). The chemical inertness of the C–F bond makes the chemistry of C–F bond activation a specialized field. Fluorine forms the strongest σ bond to carbon and its high electronegativity leads to a significant ionic bond character. Likewise, fluorine substituents are weak Lewis bases and fluoride is a poor leaving group, which all give rise to a high thermodynamic stability and a kinetic inertness of C–F bonds (Kuehnel et al., 2013).

Outstanding progress has been made recently in transition-metal-mediated C–F activation reactions and in the development of new catalytic processes for the functionalization of fluoroorganics (Murphy et al., 1997; Jones, 2003; Mazurek and Schwarz, 2003; Ahrens et al., 2015). Research on transition metal-catalyzed C (*sp*^2^)–F bond activation has been intensively studied (Amii and Uneyama, 2009; Braun et al., 2009; Laot et al., 2010; Ohashi et al., 2011; Lv et al., 2012; Fujita et al., 2016) while C(*sp*^3^)–F bond activation needs much more exploration (Choi et al., 2011; Benedetto et al., 2012; Blessley et al., 2012; Kuehnel et al., 2012; Zhang et al., 2015; Huang and Hayashi, 2016). Recently, Prof. Ichikawa's group (Ichitsuka et al., 2014) developed an efficient synthesis of 2-fluoro-1,3-cyclopentadienes through 2-trifluoromethyl-1-alkenes with alkynes by sequential β-fluorine elimination of a trifluoromethyl group (Equation 1).


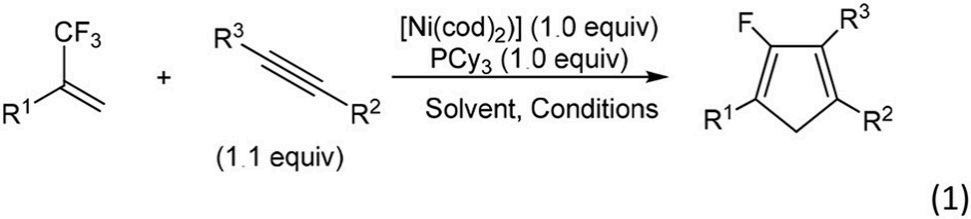


**Scheme 1:**
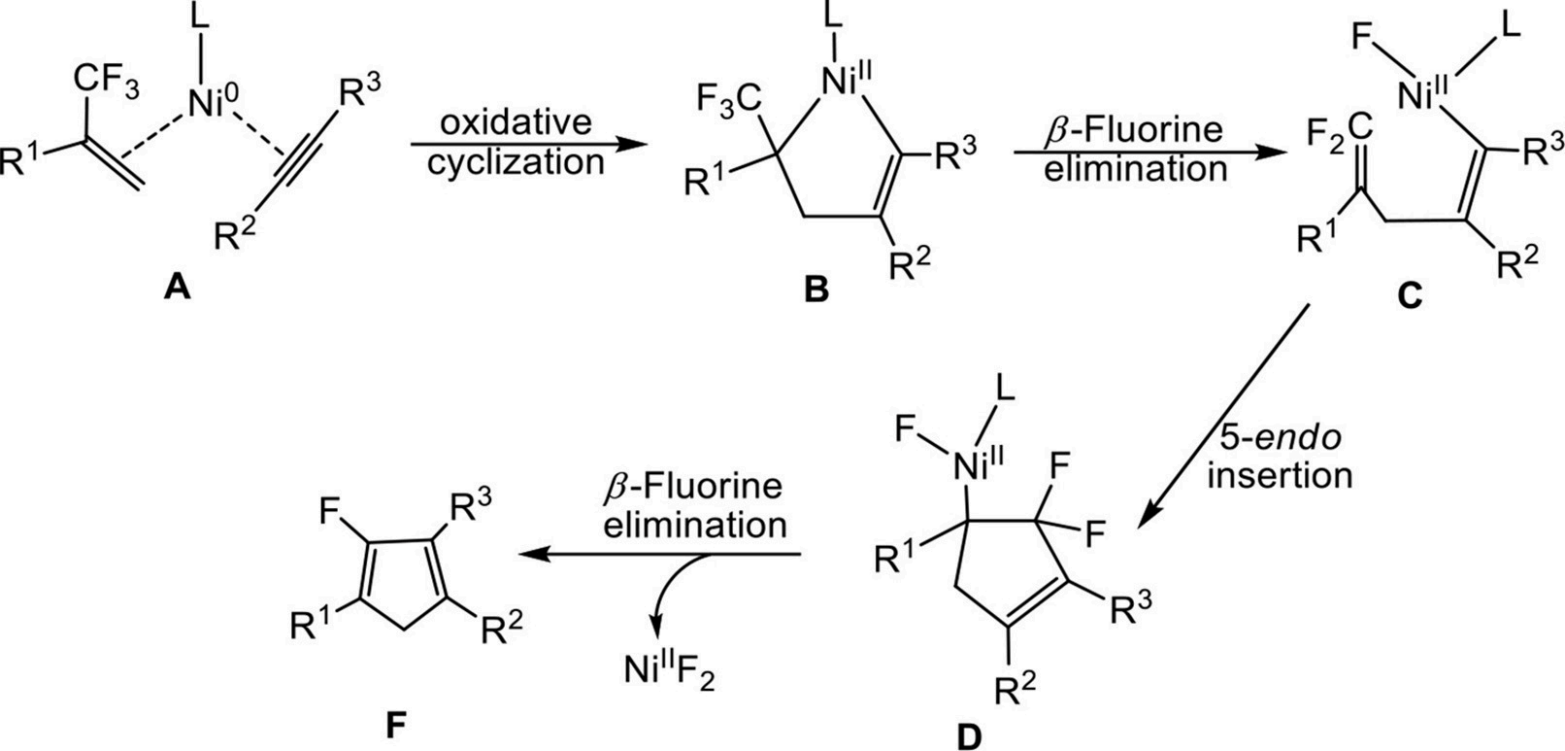
A plausible mechanism proposed on the basis of the experimental observations.

To account for the reactions, the authors proposed a plausible reaction pathway on the basis of the experimental observations (Scheme 1). In the proposed mechanism, nickelacyclopentene **B** bearing a trifluoromethyl group was formed by oxidative cyclization of 2- trifluoromethyl-1-alkene and alkyne with Ni^0^L reactant. Subsequently, *β*-fluorine elimination involving one of C–F bond of trifluoromethyl in organonickel **B** then generates the intermediate **C**, followed by intramolecular 5-*endo* insertion of difluoroalkene leading to the formation of difluorocyclopentenylnickel **D**. Finally, in **D**, the second *β*-fluorine elimination of Ni^II^F_2_ yields the product 2-fluoro-1, 3-cyclopentadienes.

In this article, we plan to elucidate the detailed mechanism of the nickel-mediated [3+2] cycloaddition reaction shown in Equation (1) and explore the sequential *β*-fluorine elimination and the normally disfavored insertion in **C** via density functional theory (DFT) calculations. Moreover, we are interested in another different addition method from **A** to **B**, involving the alkyne insertion process. Depending on the calculations we obtained, we discuss the regio-selectivity of asymmetric alkynes.

## Computational details

The geometries of all reported reactants, intermediates, transition states, and products were optimized at the DFT level using the B3LYP hybrid functional (Lee et al., 1988; Miehlich et al., 1989; Becke, 1993; Stephens et al., 1994). All of the Ni and P atoms in this analysis were described using the LanL2DZ basis set, including a double-ζ valence basis set with the effective core potentials (ECPs) of Hay and Wadt (1985a,b; Wadt and Hay, 1985). Polarization functions were added for Ni (ζ_*f*_ = 3.130) and P (ζ_*d*_ = 0.340) (Huzinaga, 1985; Ehlers et al., 1993; Höllwarth et al., 1993). The 6-31G(d) basis set was used for other atoms (Hariharan and Pople, 1973; Koseki et al., 1992). Vibrational frequency calculations were carried out at the same level to confirm the nature of all of the optimized structures as the minima (zero imaginary frequency) or transition states (one imaginary frequency). Each of the calculated transition states connects two relevant minima were further confirmed by intrinsic reaction coordinates (IRC) calculations (Fukui, 1970, 1981). In consideration of the dispersion and solvation effects, single-point energy calculations (based on the gas-phase optimized geometries) were performed at the M06 (Truhlar, 2008; Zhao and Truhlar, 2008a,b, 2009) level of theory in conjunction with the solvation model density (SMD) continuum method (Marenich et al., 2009). According to the reaction conditions, 1,4-dioxane was employed as the solvent in the SMD calculations. All the single-point calculations employ a larger basis set SDD for Ni and 6-311++G(d,p) for other atoms, respectively, except P atom remains unchanged. In this paper, the dispersion- and solvation-corrected free energies are used for discussion throughout the text. All calculations were performed using the Gaussian 09 software package (Frisch et al., 2009). And the selected calculated structures were visualized using the XYZviewer software (de Marothy, 2010). Cartesian coordinates and total energies for all of the calculated structures are listed in Supplementary Table 1.

## Results and discussion

### Mechanisms of the NI-mediated reaction shown in scheme 1

**Scheme 2:**
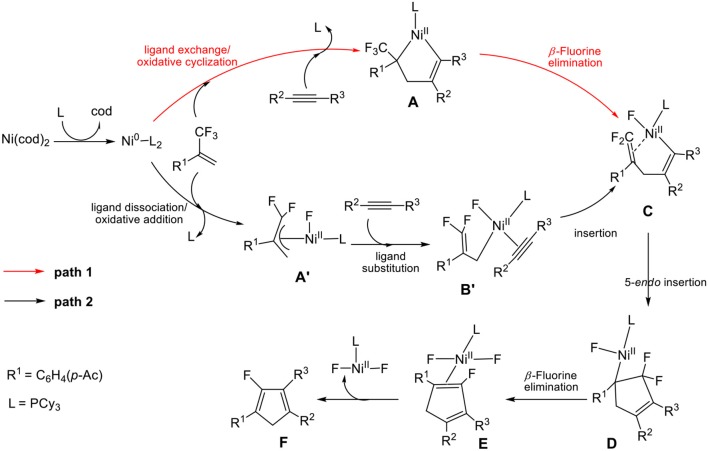
Two different oxidative addition pathways for the Ni-mediated reaction.

In the nickel-mediated cycloaddition reaction, we expanded two possible oxidative addition methods in Scheme 2 to better clarify the detailed reaction mechanism in Equation (1). **Path 1** starts from ligand exchange and oxidative cyclization of fluorine-containing alkenes and alkynes with Ni^0^ in complex **A**, which affords a five-membered nickelacycle **B**, followed by *β*-fluorine elimination gives **C**. However, **path 2** proceeds with ligand dissociation and oxidative addition of the C-F bond in 2-trifluoromethyl-1-alkenes, which results in the formation of the conjugated species **A**′, followed by the ligand substitution gives **B**′. Then, the insertion of alkynes leads to the formation of species **C**. **C** is the common species for both **path 1** and **path 2**. In the following step, the intramolecular 5-*endo* insertion generates a new five-membered difluoro-cyclopentadiene **D**. *β*-fluorine elimination of **D** affords the Ni(II) species, from which leaving of the NiF_2_L_*n*_, provides the monofluorinated cyclopentadiene product.

**Figure 1:**
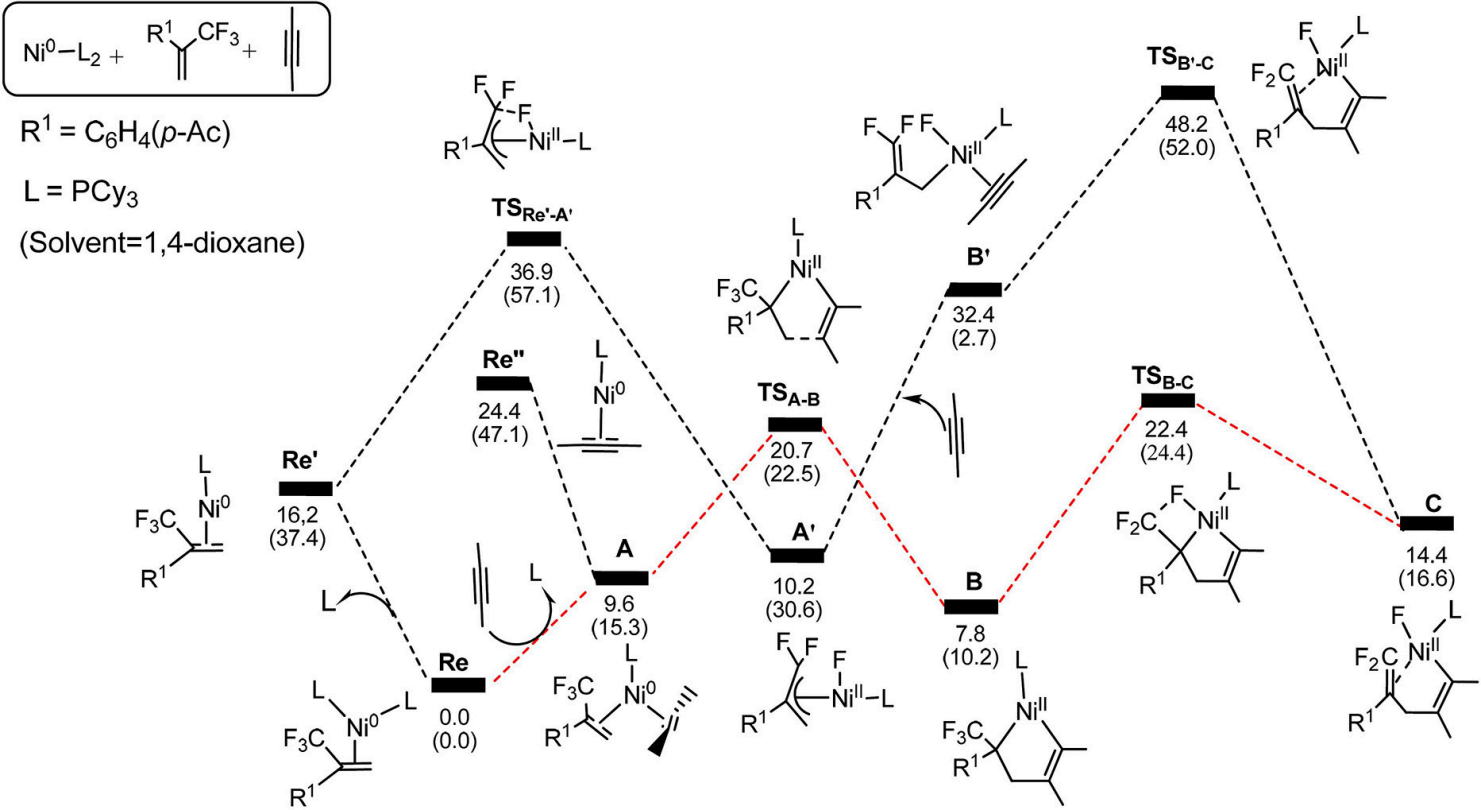
Energy profiles calculated for the oxidative cyclization (**TS**_A−B_) and *β*-Fluorine elimination (**TS**_B−C_) (**path 1** in red line), oxidative addition (**TS**${}_{\text{R}{\text{e}}^{\text{′}}-A^{\prime }}$), ligand substitution (**A**′ → **B**′) and alkyne insertion (**TS**${}_{{\text{B}}^{\text{′}}-C}$) (**path 2** in black line). The solvation-corrected relative free energies and electronic energies (in parentheses) are given in kcal/mol.

**Figure 2:**
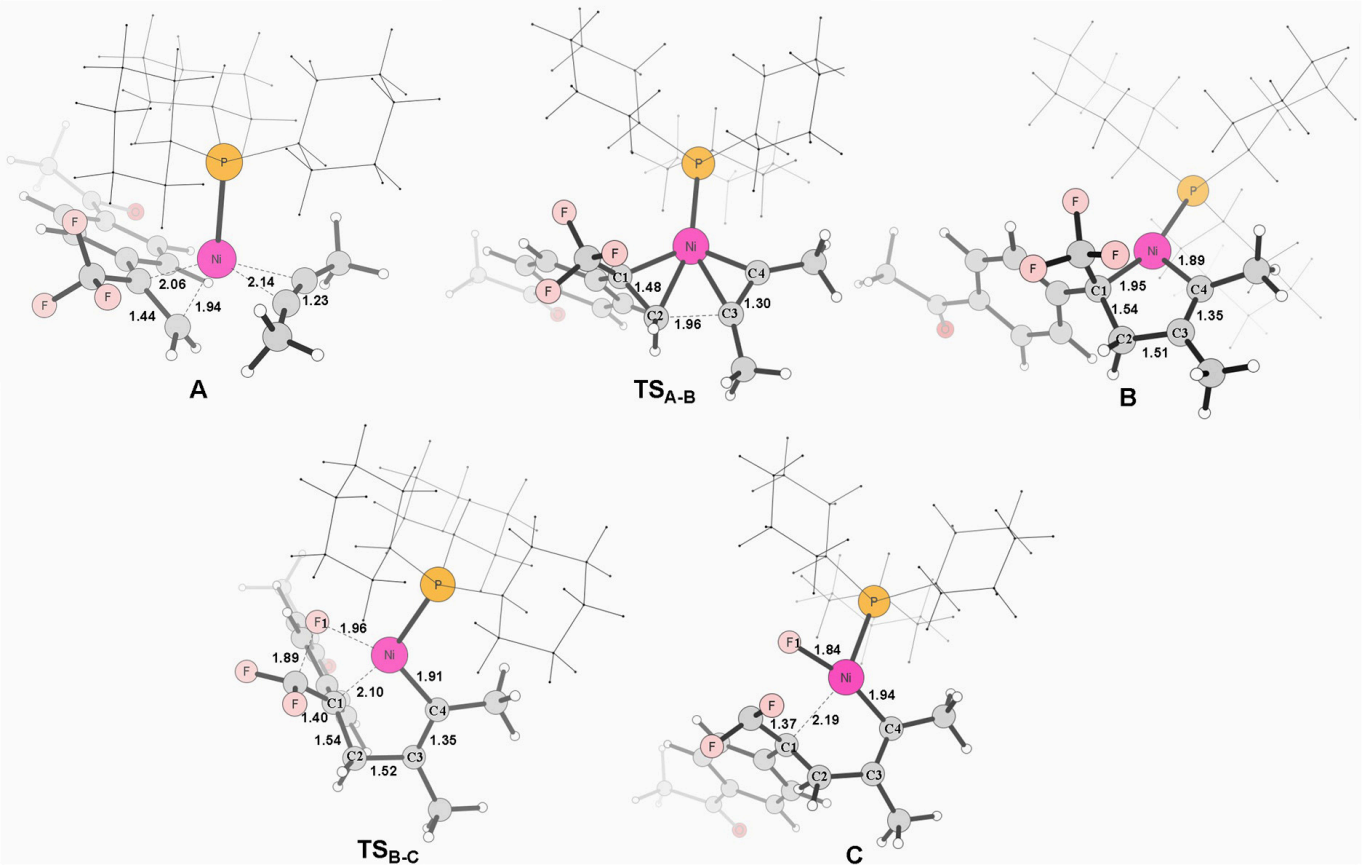
Structures calculated for selected intermediates and TSs in the oxidative cyclization (**TS**_A−B_) and *β*-fluorine elimination (**TS**_B−C_) (**path 1** in red line). The distances are given in angstroms **(**Å).

**Figure 3:**
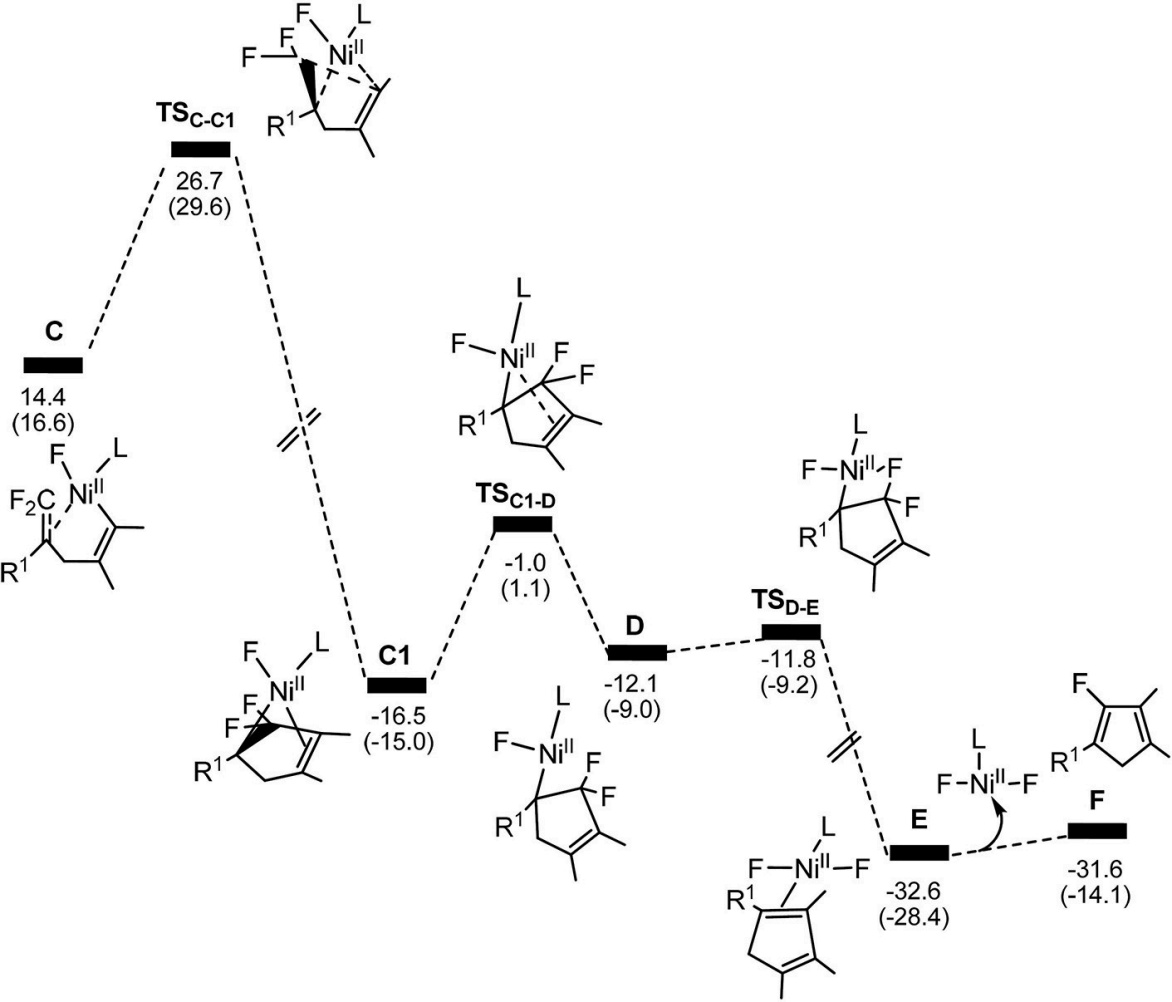
Calculated energy profiles for the pathway starting from complex **C** to the product. The solvation-corrected relative free energies and electronic energies (in parentheses) are given in kcal/mol.

**Figure 4:**
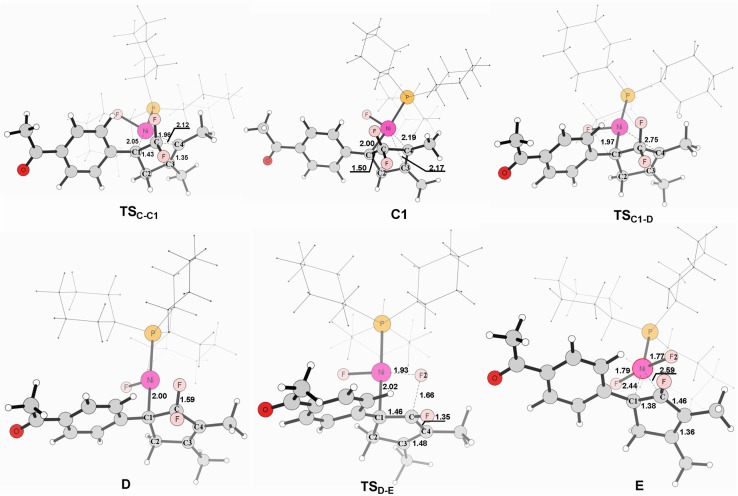
Structures calculated for selected intermediates and TSs for complex **C** to product. The distances are given in angstroms (Å).

On the basis of the mechanism shown in Scheme 2, we select butyne as simple substitute for symmetrical experimental model substrates 4-octyne and employ PCy_3_ as ligand in our calculations. The calculated energy profiles are presented in the Figures 1, 3, and the selected optimized structures of intermediates and transition states are depicted in Figures 2, 4.

Firstly, we focus on the two different oxidative addition mechanisms shown in Figure 1. Ni(0) establishes initial contact with alkene through the way of π-coordination and two PCy_3_ ligands, resulting in a stable three-component complex **Re**, which is lower in energy than the two-component complexes, **Re**′ and **Re**′′, by 16.2 and 24.4 kcal/mol, respectively. For **path 1**(in red line), ligand exchange of complex **Re** with one PCy_3_ ligand to form another three-component complex **A**, which is found to be 9.6 kcal/mol higher than **Re** in free energy. **A** undergoes oxidative addition to form the metallacycle intermediate **B** via the transition state **TS**_A__−**B**_ with a barrier of 11.1 kcal/mol. **TS**_A−B_ has C1-C2, C2-C3, and C3-C4 distances of 1.48, 1.96, and 1.30 Å, respectively (Figure 2). In **B**, both C = C of alkene and C ≡ C of alkyne were elongated to 1.54 and 1.35 Å, respectively. Subsequently, the first *β*-fluorine elimination reaction occurs from complex **B** in **path 1** to **C** through the transition state **TS**_B−C_, in which the migration of F1 atom from CF_3_ to Ni(II) center via the cleavage of the C–F bond and C–Ni bond and the formation of Ni–F bond simultaneously with a barrier of 14.6 kcal/mol. The cleavage of C–F1, Ni–C1 and formation of Ni–F1 bond occur synchronously, and the C–F1, Ni-F1 and Ni–C1 distances being 1.89, 1.96, and 2.10 Å, respectively, as shown in Figure 2. From Figure 1, we can see that *β*-fluorine elimination is the most energy-demanding for the conversion from **Re** to **C**. The overall barrier calculated for **Re** to **C** is 22.4 kcal/mol (**Re** → **TS**_B−C_).

For **path 2** (in black line), the reaction starts with **Re**′, followed by the oxidative addition of the C-F bond to Ni(0) via the transition state **TS**_Re'-A'_, generating the corresponding π-allylnickel complex **A**′ with a barrier of 20.7 kcal/mol. The coordination of the alkyne CH_3_C=CCH_3_ gives the precursor intermediate **B**′ ready for alkyne insertion. Alkyne insertion leads to formation of **C**, having an approximate ring structure with the Ni metal center being coordinated with the alkenyl moiety, via a very high barrier transition state (38.0 kcal/mol, Figure 1). The C1–C(F_2_) distance is shorten to 1.37 Å (Figure 2). The alkyne insertion is the most energy-demanding for the conversion from **Re**′ to **C**. The overall barrier calculated for **Re**′ to **C** is 38.0 kcal/mol (**A**′ → **TS**_B'-C_). It is worth of note that the pathway involving the alkyne insertion **path 2**, have considerably higher barrier than the **path1** (Figure 1).

From complex **C**, both **path 1** and **path 2** would undergo the same reaction process 5-*endo* insertion through transition state **TS**_C−C1_, shown in Figure 3, a disfavored process for the construction of five-membered rings in general because of the severe distortions required in the reaction geometry (Sakoda et al., 2005; Ichikawa et al., 2006). However, complex **C** possesses an unique moiety of 1,1-difluoro-1-alkenes, making even a nucleophilic 5-*endo*-*trig* approach feasible. The polarization of the C = C bond caused by the two fluorines (Chambers, 1973; Banks et al., 1994; Sakoda et al., 2005; Fujita et al., 2014) results in electrostatic attraction for an intramolecular nucleophile to overcome the difficulty of the formation of ring complex precursor **C1** with a barrier of 12.4 kcal/mol (Figure 3). The distance of C(F_2_)–C4 is close to 2.12 Å (Figure 4). Then, **C1** isomerizes to the difluorocyclopentenylnickel**D** by breaking the Ni center and C = C bond with a barrier of 15.5 kcal/mol. In **D**, the Ni metal center has no interaction with C3 atom, as indicated by the long Ni–C(F_2_) and Ni–C3 distances of 2.49 and 3.27 Å, respectively. Whereafter, *β*-fluorine elimination in **D** forming the 5-*endo*-*trig* species **E** via the transition state **TS**_D−E_ only need to overcome an energy barrier of 0.3 kcal/mol. In this transition state, cleavage of C–F2 and re-bonding of Ni–F2 occur synchronously with the distances being 1.66 and 1.93 Å, respectively. The distance of Ni–F2 decreases to 1.77 Å in complex **E**. Finally, the dissociation of difluorides would yield the desired 2-fluoro-1,3-cyclopentadienes product.

On the basis of the calculations shown in Figures 1, 3, it is notable that the pathway involving alkyne insertion (**path 2** in Figure 1) has considerably higher barriers than that involving oxidative cyclization (**path 1** in Figure 1). And the 5-*endo* insertion transition state is rate-determining for the whole Ni-mediated reaction and the overall energy barrier is calculated to be 26.7 kcal/mol, corresponding to the energy difference between **TS**_C−C1_ and complex **Re** (Figures 1, 3). The first *β*-fluorine elimination process is endergonic. Therefore, the combined processes of the *β*-fluorine elimination and the 5-*endo* insertion can be viewed as the rate-determining step because the endergonicity of the *β*-fluorine elimination process contributes to the overall rate-determining barrier. This finding is consistent with the recent DFT study by Bi's group (Zhang et al., 2017), in which the 5-*endo* insertion transition state is also the rate-determining for the whole Ni-mediated reaction.

### Effect of ligand

**Figure 5:**
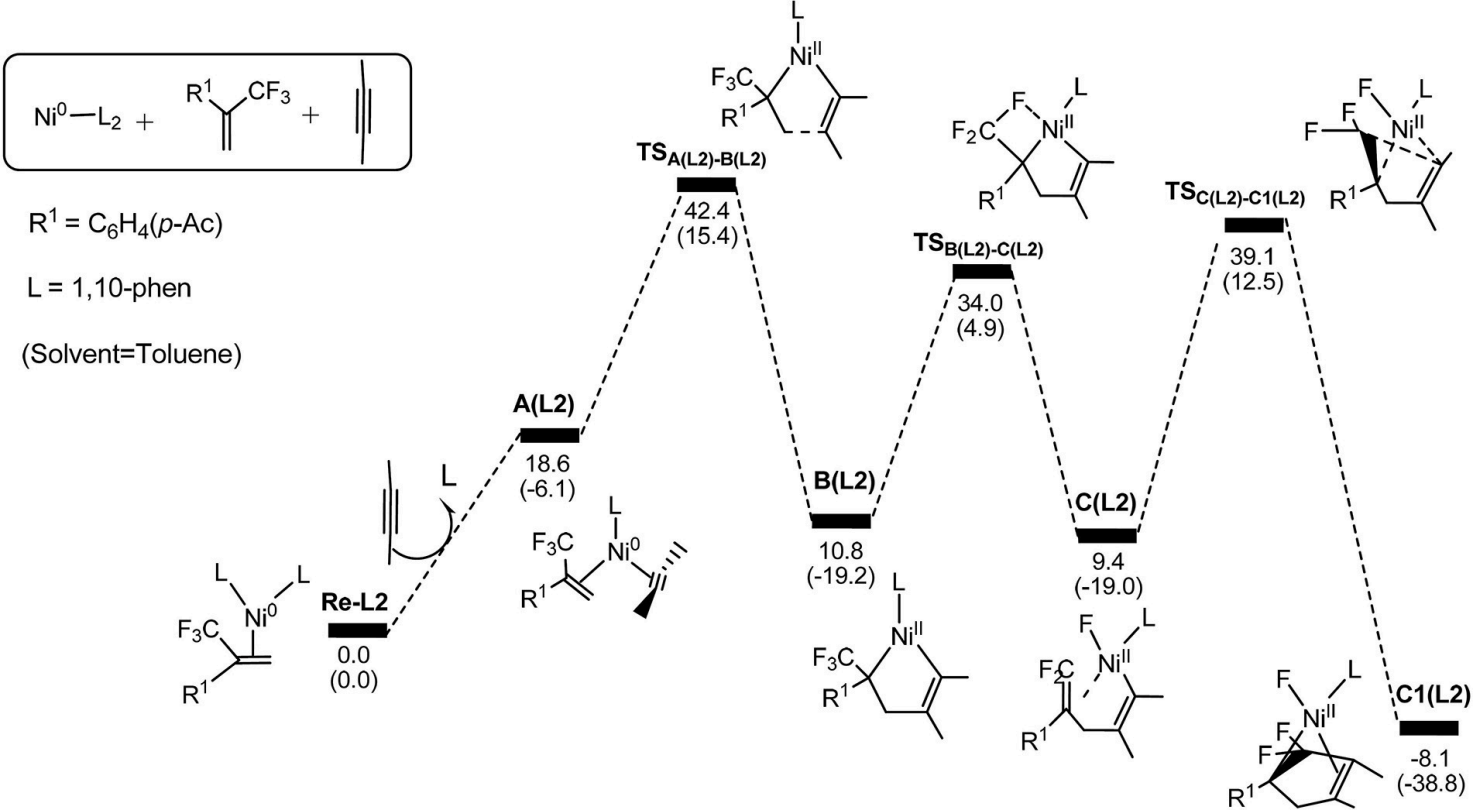
Energy profiles calculated for the **path 1** by using 1,10-phenanthroline as the ligand. The solvation-corrected relative free energies and electronic energies (in parentheses) are given in kcal/mol.

Based on the detailed mechanistic discussion above, we have achieved an understanding of the competition between two alternative pathways. Next, we turn our attention to investigate why the less electron-donating group is ineffective for the reactions. Experimentally, reactions carried out in the presence of PPh_3_ or 1,10-phenanthroline resulting in no expected cyclization products. It was also found that when the reaction was carried out in strong electron-donating ligands, IMes or PCy_3_, the yield was unexpectedly improved. In order to understand the effect of the ligands on the reaction outcomes, we calculated the energy profiles for the **path 1** (from the reactants to 5-*endo* insertion process) by selecting 1,10-phenanthroline as the ligand (Figure 5).

In Figure 5, we can clearly see that the oxidative cyclization transition state is the rate-determining from **Re-L2** to **C1(L2)** reaction and the overall barrier is calculated to be 42.4 kcal/mol, implying the oxidative cyclization process is infeasible under ambient conditions. This result is consistent with the experimental observation that electron-withdrawing ligand do not promote the reactions. The reason is that the oxidation reaction occurs easily on the highly electron-rich Ni(0) species derived from strong electron-donating ligands.

### Regioselectivity

In this section, we examine the origin for the regioselectivity observed for the reactions of unsymmetrical alkynes. When asymmetric alkynes (in Equation 1) are used, formation of regioisomeric products is highly possible. Experimentally, for the [3+2] cycloaddition of 2-trifluoromethyl-1-alkenes with asymmetric alkynes, the resulting products give complete regioselectivity with the R^3^-substituent carbon coupling with the fluorine-bonded carbon (Ichitsuka et al., 2014). For example, 4-methyl-2-pentyne (MeC ≡ C*i*-Pr) and 1-phenyl-1-propyne (MeC ≡ CPh) were employed to illustrate the regio-selectivity for the cycloaddition reaction. When 4-methyl-2-pentyne was used as the alkyne substrate, the difluoro carbon atom preferred to couple with the methyl-substituted carbon. While the alkyne 1-phenyl-1-propyne preferred to couple with the phenyl-substituted carbon rather than methyl-substituted carbon.

**Figure 6:**
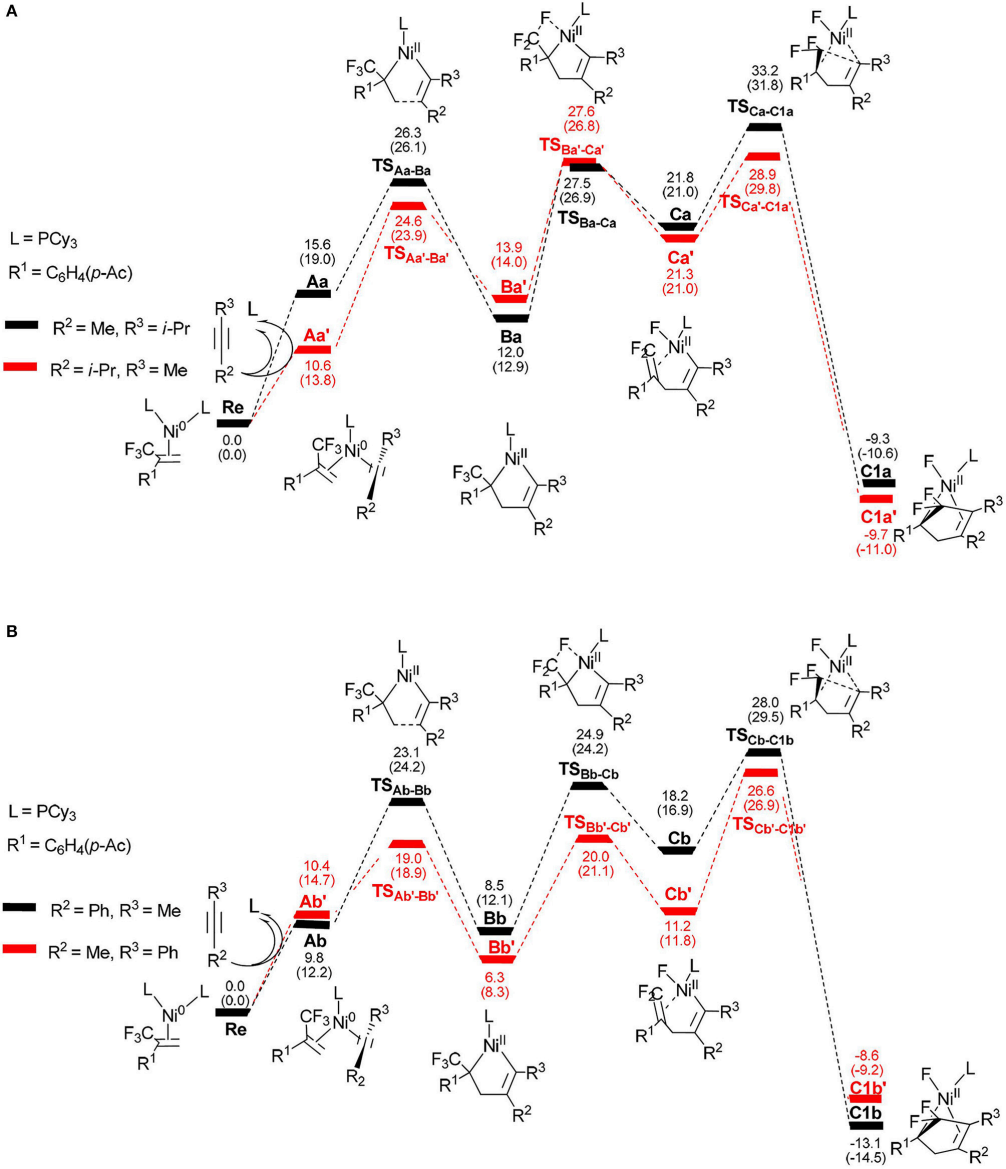
Energy profiles calculated for the reactions of unsymmetrical alkynes, 4-methyl-2-pentyne **(A)** and 1-phenyl-1-propyne **(B)**, to complexes **C1a**/**C1a**′ and **C1b**/**C1b**′, respectively. The solvation-corrected relative free energies and electronic energies (in parentheses) are given in kcal/mol.

**Figure 7:**
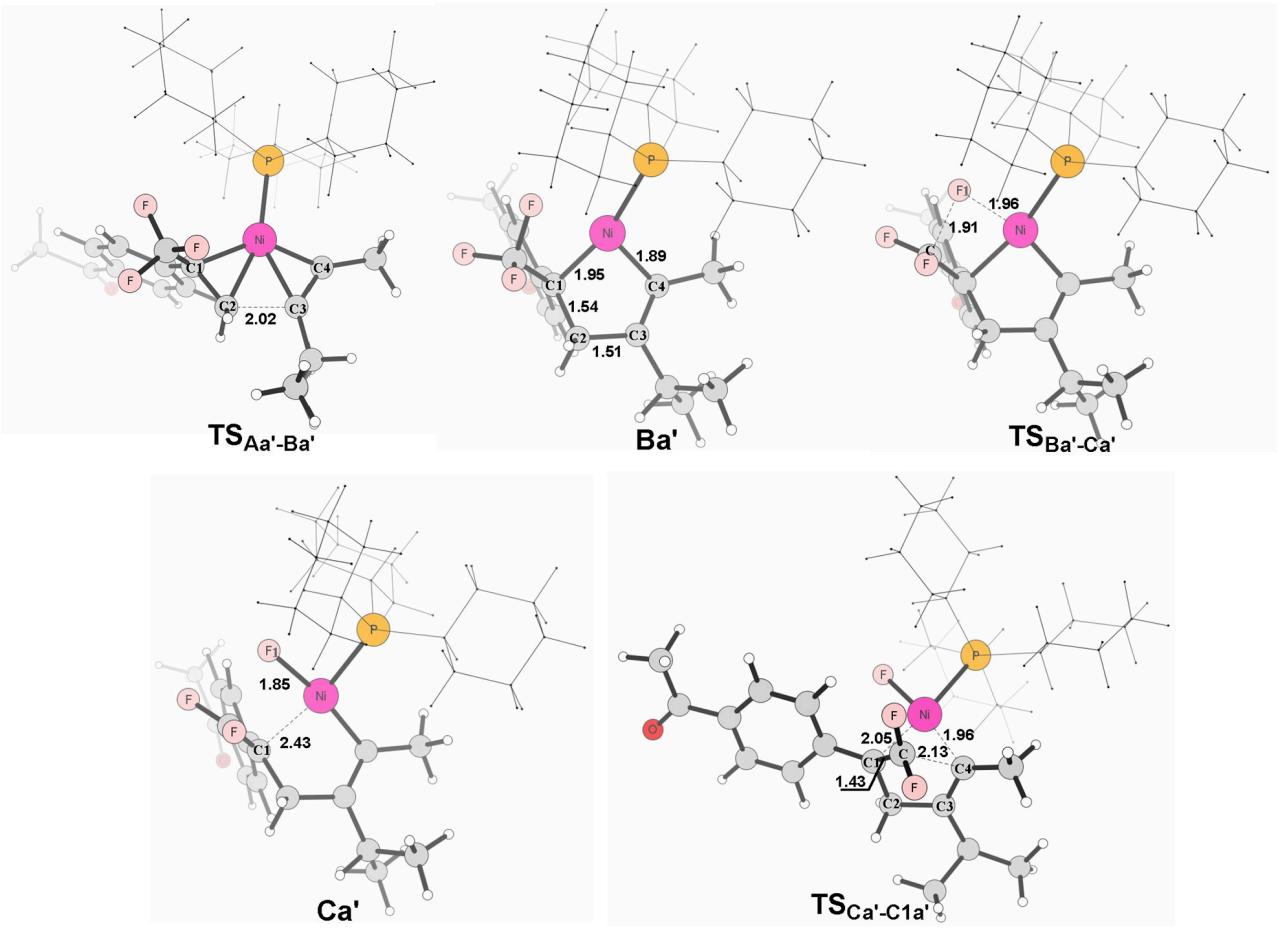
Structures calculated for selected intermediates and TSs for path in Figure 6A. The distances are given in angstroms (Å).

**Figure 8:**
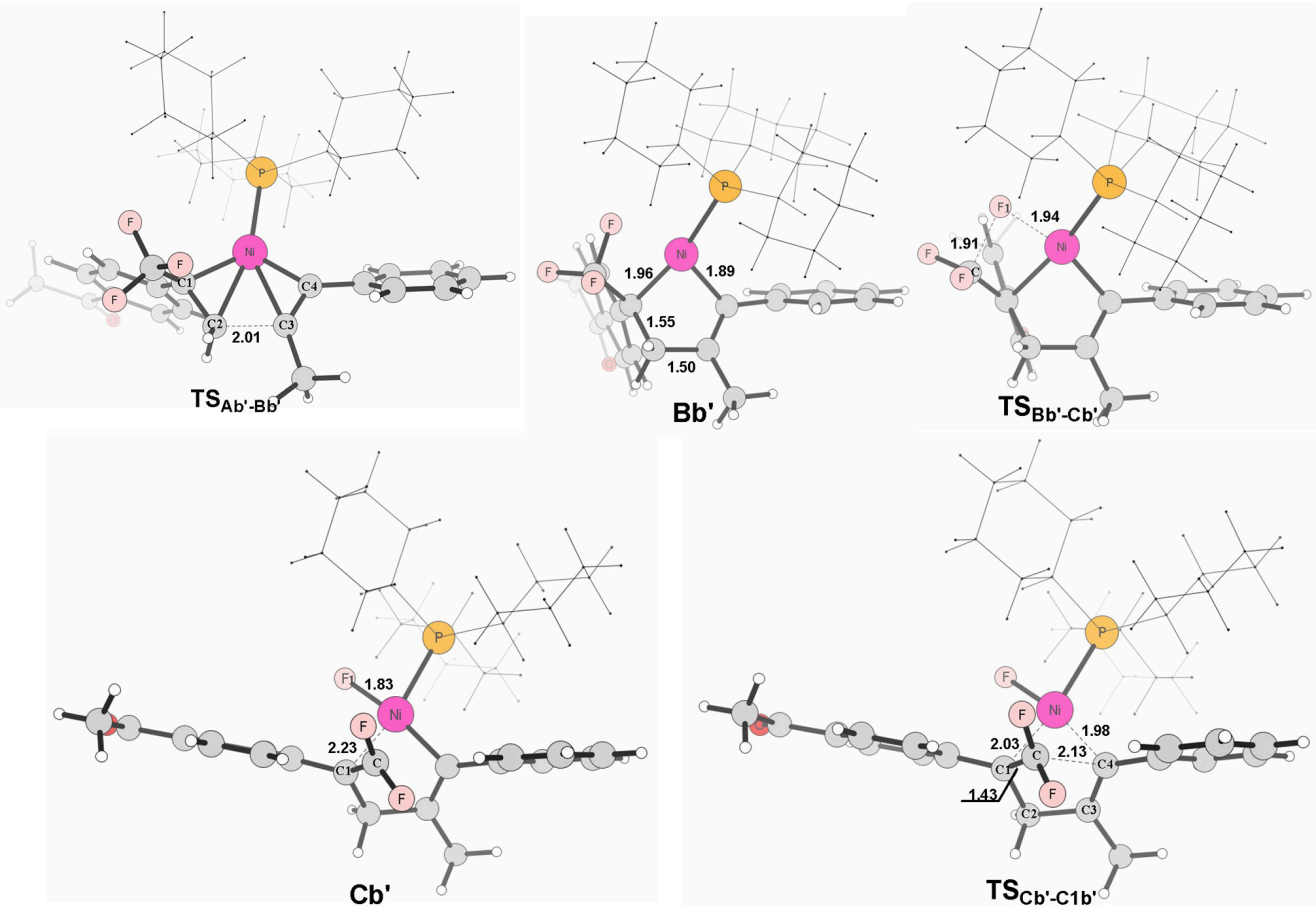
Structures calculated for selected intermediates and TSs for path in Figure 6B. The distances are given in angstroms (Å).

To better understand the regioselectivity observed, we calculated the energy profiles for the reaction shown in Figure 6. The calculated molecular structures for selected intermediates and transition states are also presented in Figures 7, 8. On the basis of the proposed mechanism shown in Scheme 2, the regioselectivity was initially controlled by the oxidative coupling step and it is obvious that the most favorable pathway is following the red line for 4-methyl-2-pentyne (Figure 6A) and 1-phenyl-1-propyne (Figure 6B). The overall barriers for the more favorable cycloaddition pathway were calculated to be 28.9 kcal/mol (**Re** → **TS**${}_{\text{C}{\text{a}}^{\text{′}}-\text{C}1{\text{a}}^{\text{′}}}$, Figure 6A) and 26.6 kcal/mol (**Re** → **TS**${}_{\text{C}{\text{b}}^{\text{′}}-\text{C}1{\text{b}}^{\text{′}}}$, Figure 6B), respectively. The calculation results are consistent with the experimentally observed regioselectivity (Ichitsuka et al., 2014).

It is well-known that the oxidation state of metal nickel changes from 0 to +2 through **TS**_Aa−Ba_/**TS**${}_{\text{A}{\text{a}}^{\text{′}}-Ba^{\prime }}$ and **TS**_Ab−Bb_/**TS**${}_{\text{A}{\text{b}}^{\text{′}}-Bb^{\prime }}$, in which the coordinated alkyne acts as a nucleophile and favorably attacks metal center. The alkyne 4-methyl-2-pentyne bears two electron-donating groups methyl and isopropyl, by contrast, *i*-Pr plays a larger role. It makes the methyl-substituted carbon of MeC ≡ C*i*-Pr π electron richer in comparison with the *i*-Pr-substituted carbon, facilitating both the oxidative addition and electrostatic attraction between the –CF_2_ carbon and the internal nucleophile. As a result, the desired pathway (red line in Figure 6A) is both electronically and sterically favorable for alkyne 4-methyl-2-pentyne by oxidative cycloaddition. The energy barrier of the cycloaddition is 14.0 kcal/mol through the transition state **TS**${}_{\text{A}{\text{a}}^{\text{′}}-Ba^{{}^{\prime }}}$, in which the C1–C2 distance is 2.02 Å and the *i*-Pr substituent is far away from the metal-bonded ligands (Figure 7). 5-*endo* insertion of **Ca**′ to form **C1a**′ via the transition state **TS**${}_{\text{C}{\text{a}}^{\text{′}}-C1a^{\prime }}$is required to overcome an energy barrier of only 7.6 kcal/mol because of the electrostatic attraction. The distance of C(F_2_)–C4 in **TS**${}_{\text{C}{\text{a}}^{{}^{\text{′}}}-C1a^{{}^{\prime }}}$is 2.13 Å (Figure 7).

When used the alkyne 1-phenyl-1-propyne, the electron-accepting phenyl makes the Me-substituted carbon of MeC ≡ CPh π electron poorer in comparison with the Ph-substituted carbon. Therefore, the nickel center is inclined to coupling with the Ph-substituted carbon no matter for oxidative cyclization or for electrostatic attraction, with the energy barriers of 8.6 kcal/mol and 15.4 kcal/mol, respectively (red line Figure 6B). The distances of C1–C2 in **TS**_Ab'-Bb'_ and C(F_2_)–C4 in **TS**_Cb'-C1b'_ are 2.01 and 2.13 Å, respectively, shown in Figure 8.

## Conclusions

The detailed reaction mechanism for Ni-mediated [3+2] cycloaddition of 2-trifluoromethyl-1-alkenes with alkynes leading to formation of cyclopentadiene derivatives have been studied with the aid of DFT calculations. For the energetics associated with the reaction, two possible oxidative additions were investigated in detail. **Path 1** starts from ligand exchange of complex **Re** to form **A**. Then, the oxidative cyclization of fluorine-containing alkenes and alkynes with Ni^0^ in complex **A**, which affords a five-membered nickelacycle **B**, followed by *β*-Fluorine elimination gives **C**. However, **path 2** proceeds with oxidative addition of the C-F bond in 2-trifluoromethyl-1-alkenes, which results in the formation of the conjugated species **A**′, followed by the ligand substitution gives **B**′. Then, the insertion of alkynes leads to the formation of species **C**. **C** is the common species for both **path 1** and **path 2**. In the following step, the intramolecular 5-*endo* insertion generates a new five-membered difluoro-cyclopentadiene **D**. *β*-Fluorine elimination of **D** affords the Ni(II) species, from which leaving of the NiF_2_L_*n*_, provides the monofluorinated cyclopentadiene product. The pathway involving the alkyne insertion **path 2** have considerably higher barrier than the **path1**. The combined processes of the *β*-fluorine elimination and the 5-*endo* insertion can be viewed as the rate-determining step according to the calculation results. The overall free energy barrier for the whole reaction was computed to be 26.7 kcal/mol (**Re** → **TS**_C−C1_).

The regioselectivity was also investigated using the unsymmetric alkynes 4-methyl-2-pentyne (MeC ≡ C*i*-Pr) and 1-phenyl-1-propyne (MeC ≡ CPh) as the substrate molecules. The alkyne MeC ≡ C*i*-Pr bears two electron-donating groups methyl and isopropyl, by contrast, *i*-Pr plays a larger role. It makes the methyl-substituted carbon of MeC ≡ C*i*-Pr π electron richer in comparison with the *i*-Pr-substituted carbon, facilitating both the oxidative cyclization and electrostatic attraction between the –CF_2_ carbon and the internal nucleophile. When used the alkyne MeC ≡ CPh, the electron-accepting phenyl makes the Me-substituted carbon of MeC ≡ CPh π electron poorer in comparison with the Ph-substituted carbon. Therefore, the nickel center is inclined to coupling with the Ph-substituted carbon no matter for oxidative cyclization or for electrostatic attraction, eventually leading to formation of the experimentally observed regioisomer.

## Author contributions

The work cannot be completed without kind cooperation of all authors. W-JC, BW, and XH were responsible for the study of concept and design of the project. R-NX and WL were responsible for the searching intermediates and transition states, analysis of data and drawing energy profiles. W-JC, XS, and Q-HW drafted the manuscript. All authors contributed to its final form and gave final approval for its publication.

### Conflict of interest statement

The authors declare that the research was conducted in the absence of any commercial or financial relationships that could be construed as a potential conflict of interest.

## Supplementary material

The Supplementary Material, including Cartesian coordinates and total energies for all of the calculated structures, can be found online at: https://www.frontiersin.org/articles/10.3389/fchem.2018.00319/full#supplementary-material
