# Supplementary material for: DFT Studies on Ni-Mediated C–F Cleavage for the Synthesis of Cyclopentadiene Derivatives
Source: Front Chem. 2018 Aug 13;6:319. doi: 10.3389/fchem.2018.00319 (PMC6099089; doi:10.3389/fchem.2018.00319)
Supplement: Supplementary file 1 [file Table_1.PDF]

Supplementary Material

DFT Studies on Ni-mediated C−F Cleavage for the Synthesis of Cyclopentadiene Derivatives

Wen-Jie Chen[1,*], Ruo-Nan Xu[2], Weimin Lin[1], Xuejiao Sun[1], Bin Wang[2,*] Qi-Hui Wu[1], and Xin Huang[2]

[1] *Department of Material Chemistry, College of Chemical Engineering and Materials Science, Quanzhou Normal University, Quanzhou 362000, China*

[2] *College of Chemistry, Fuzhou University, Fuzhou, Fujian 350116, China*

***Correspondence:** Wen-Jie Chen：chenwenjie@qztc.edu.cn;

Bin Wang：wangbin_100@fzu.edu.cn

Cartesian coordinates and total energies for all of the calculated structures

129

Re SCF Done: E(RB3LYP) = -2393.3273266

6	-0.428770000	3.088762000	1.211120000
1	-1.326924000	2.697499000	1.701636000
6	-0.699498000	4.568395000	0.868229000
1	0.188390000	5.015856000	0.402057000
1	-1.514725000	4.661334000	0.141042000
6	-1.050676000	5.366498000	2.138691000
1	-1.209373000	6.422386000	1.882787000
1	-2.004407000	4.995134000	2.541345000
6	0.038114000	5.234776000	3.214261000
1	0.958096000	5.722847000	2.857828000
1	-0.263786000	5.767549000	4.125177000
6	0.338479000	3.761731000	3.530495000
1	-0.541071000	3.296805000	3.997431000
1	1.156200000	3.688760000	4.259412000
6	0.699421000	2.972494000	2.260354000
1	0.865926000	1.921074000	2.512277000
1	1.641695000	3.361352000	1.855506000
6	0.986606000	2.610300000	-1.492591000
1	1.268225000	1.734003000	-2.089601000
6	0.471460000	3.680992000	-2.480426000
1	-0.402070000	3.320863000	-3.031488000
1	0.150609000	4.575921000	-1.931097000
6	1.557011000	4.074273000	-3.501842000
1	1.776807000	3.206029000	-4.141056000
1	1.168367000	4.857289000	-4.165776000
6	2.852266000	4.540392000	-2.825473000
1	3.618861000	4.760856000	-3.579280000
1	2.664055000	5.480432000	-2.285830000
6	3.359753000	3.485325000	-1.834380000
1	4.254438000	3.849903000	-1.312720000
1	3.665292000	2.583478000	-2.386537000
6	2.272841000	3.117652000	-0.809795000
1	2.650493000	2.370233000	-0.107520000
1	2.043262000	4.011197000	-0.217178000
6	-1.906867000	2.107896000	-1.161653000
1	-1.853086000	3.107162000	-1.610511000
6	-3.168884000	2.086277000	-0.277866000
1	-3.247219000	1.127901000	0.242263000

1	-3.112277000	2.860504000	0.495261000
6	-4.437089000	2.316755000	-1.122166000
1	-4.416541000	3.337041000	-1.535128000
1	-5.320365000	2.261466000	-0.472892000
6	-4.558970000	1.306959000	-2.271841000
1	-4.706436000	0.301552000	-1.855607000
1	-5.444249000	1.529879000	-2.881448000
6	-3.297764000	1.309515000	-3.145888000
1	-3.213663000	2.275780000	-3.666408000
1	-3.372022000	0.540171000	-3.925399000
6	-2.032663000	1.074943000	-2.302446000
1	-1.147642000	1.100563000	-2.952699000
1	-2.071576000	0.069613000	-1.863221000
15	-0.262138000	1.853495000	-0.227812000
28	0.070271000	-0.362262000	0.610207000
6	-0.464856000	-1.835825000	1.734853000
1	0.191045000	-1.906700000	2.600318000
1	-0.641889000	-2.795677000	1.252008000
6	-1.565708000	-0.910669000	1.800776000
6	-1.655676000	-0.124489000	3.072351000
9	-2.330636000	1.058086000	2.943455000
9	-2.301994000	-0.790753000	4.073762000
9	-0.433514000	0.193257000	3.578794000
6	-2.851802000	-1.278606000	1.147936000
6	-2.848372000	-2.058628000	-0.033129000
6	-4.117536000	-0.934261000	1.671043000
6	-4.021317000	-2.475461000	-0.641008000
6	-5.295893000	-1.350958000	1.057191000
6	-5.276197000	-2.131808000	-0.107711000
1	-4.191920000	-0.346194000	2.576587000
1	-3.996279000	-3.076275000	-1.544891000
1	-6.241752000	-1.065402000	1.508644000
1	-1.899223000	-2.334204000	-0.479463000
8	-6.435642000	-3.281673000	-1.821364000
6	-6.506347000	-2.611614000	-0.798521000
6	-7.864868000	-2.257149000	-0.208852000
1	-7.966942000	-2.647096000	0.811110000
1	-8.003179000	-1.170454000	-0.157299000
1	-8.642875000	-2.690163000	-0.840198000
15	2.001299000	-1.400234000	-0.144730000
6	3.630590000	-0.401992000	-0.138262000
6	4.904332000	-1.127881000	-0.617990000
1	4.742294000	-1.601160000	-1.593647000
1	5.155148000	-1.932627000	0.084120000
6	3.888557000	0.305377000	1.212807000
1	4.077117000	-0.438745000	1.994144000
1	2.995190000	0.857800000	1.525836000
6	5.102156000	1.250127000	1.139317000
1	5.281303000	1.692916000	2.127563000
1	4.878897000	2.084827000	0.460114000
6	6.361547000	0.526331000	0.641868000
1	7.196894000	1.232630000	0.554303000
1	6.665864000	-0.227781000	1.383084000
6	6.103201000	-0.165711000	-0.703451000
1	5.905383000	0.595137000	-1.473123000
1	6.995590000	-0.715274000	-1.029952000
6	2.356945000	-3.058737000	0.766306000
1	1.342640000	-3.462470000	0.872922000
6	2.903048000	-2.851063000	2.196234000
1	2.344200000	-2.066602000	2.717740000
1	3.946006000	-2.512431000	2.140921000
6	2.861298000	-4.158264000	3.006421000
1	3.265788000	-3.984020000	4.011724000
1	4.228779000	-3.828093000	-0.062532000
6	3.187303000	-4.151468000	0.053862000
1	2.806776000	-4.348955000	-0.953263000
6	3.156878000	-5.466675000	0.858376000
6	3.644443000	-5.274187000	2.301002000
1	3.559040000	-6.214888000	2.859531000
1	4.713310000	-5.013158000	2.289566000
1	3.765470000	-6.223964000	0.347316000
1	1.815021000	-4.469240000	3.139304000
1	2.126721000	-5.852421000	0.872731000
6	1.882468000	-2.002609000	-1.951042000
6	0.643222000	-2.902948000	-2.146861000
1	0.636759000	-3.730124000	-1.427809000
1	2.779910000	-2.604172000	-2.145648000
1	0.967690000	-0.240329000	-2.802168000
1	2.729320000	-0.213683000	-2.885944000
6	1.851929000	-0.863458000	-2.987525000
6	1.788003000	-1.411544000	-4.424882000
1	1.749989000	-0.576788000	-5.136700000
1	-0.256310000	-2.306815000	-1.942907000
6	0.567985000	-3.462499000	-3.578116000
6	0.580994000	-2.338982000	-4.623998000
1	-0.344638000	-1.751718000	-4.533813000
1	0.586504000	-2.760087000	-5.637232000
1	-0.335060000	-4.076064000	-3.688191000
1	1.424045000	-4.131727000	-3.750636000
1	2.713744000	-1.964902000	-4.641578000
1	3.409193000	0.387460000	-0.870756000

77

Re’ SCF Done: E(RB3LYP) = -1680.9860595

6	1.951587000	0.292498000	1.905182000
1	1.188998000	0.970522000	2.317227000
6	3.314681000	0.735110000	2.471123000
1	4.101097000	0.068767000	2.093586000
1	3.569027000	1.747631000	2.134494000
6	3.317518000	0.680049000	4.010181000
1	4.304871000	0.972809000	4.389723000
1	2.601279000	1.418940000	4.399016000
6	2.939095000	-0.716790000	4.525121000
1	3.723895000	-1.432121000	4.237356000

1	2.901728000	-0.719817000	5.621730000
6	1.594845000	-1.181878000	3.945349000
1	0.790151000	-0.539820000	4.333170000
1	1.367985000	-2.202348000	4.278539000
6	1.587029000	-1.123735000	2.408172000
1	0.602687000	-1.423892000	2.025273000
1	2.304747000	-1.856283000	2.016086000
6	3.134803000	-0.165089000	-0.956538000
1	2.758824000	-0.052028000	-1.984966000
6	4.466664000	0.608305000	-0.866035000
1	4.313207000	1.677519000	-1.052276000
1	4.883185000	0.522947000	0.144848000
6	5.487661000	0.050374000	-1.875507000
1	5.121557000	0.234218000	-2.896497000
1	6.436340000	0.594332000	-1.781676000
6	5.713856000	-1.456154000	-1.680890000
1	6.410611000	-1.836078000	-2.438703000
1	6.191852000	-1.625944000	-0.704402000
6	4.389482000	-2.232226000	-1.737366000
1	4.561419000	-3.297512000	-1.538269000
1	3.971564000	-2.167211000	-2.752705000
6	3.363131000	-1.675428000	-0.735843000
1	2.412725000	-2.216096000	-0.823180000
1	3.734476000	-1.845755000	0.284080000
6	1.666323000	2.347736000	-0.257124000
1	2.624143000	2.743049000	0.110502000
6	0.520975000	3.043989000	0.510200000
1	-0.434375000	2.589096000	0.211053000
1	0.620453000	2.882892000	1.590131000
6	0.478053000	4.556347000	0.228260000
1	1.388665000	5.025623000	0.629487000
1	-0.367038000	5.004403000	0.765835000
6	0.375061000	4.852122000	-1.273823000
1	-0.592649000	4.489381000	-1.650069000
1	0.394089000	5.934782000	-1.451038000
6	1.508835000	4.164976000	-2.046099000
1	2.470831000	4.612684000	-1.755210000
1	1.398158000	4.336513000	-3.124359000
6	1.551232000	2.652561000	-1.767487000
1	2.385975000	2.205519000	-2.319512000
1	0.634645000	2.182473000	-2.152004000
15	1.648326000	0.474996000	0.037706000
28	-0.213780000	-0.610317000	-0.531854000
6	-0.906857000	-1.160045000	-2.258530000
1	-0.252858000	-1.846723000	-2.788573000
1	-1.206962000	-0.289857000	-2.837146000
6	-1.741107000	-1.650770000	-1.213817000
6	-1.572113000	-3.086344000	-0.791983000
9	-1.336090000	-3.212116000	0.556035000
9	-2.666558000	-3.845911000	-1.040434000
9	-0.526755000	-3.691692000	-1.401204000
6	-2.966870000	-0.946679000	-0.753343000
6	-3.556348000	0.080628000	-1.525533000
6	-3.578337000	-1.250631000	0.483574000
6	-4.674183000	0.769733000	-1.082667000
6	-4.705437000	-0.562490000	0.920829000
6	-5.272039000	0.465107000	0.152136000
1	-3.167710000	-2.034481000	1.108138000
1	-5.121002000	1.551825000	-1.688377000
1	-5.141657000	-0.835126000	1.877354000
1	-3.142451000	0.324244000	-2.498387000
8	-6.925249000	2.136682000	-0.124514000
6	-6.475805000	1.238309000	0.575207000
6	-7.142717000	0.892039000	1.899412000
1	-6.447338000	1.019166000	2.737988000
1	-8.001200000	1.551222000	2.040066000
1	-7.480471000	-0.151251000	1.911539000

63

Re” SCF Done: E(RB3LYP) = -1037.6334785

6	-1.255910000	-0.695440000	-1.337040000
1	-0.628824000	-0.686577000	-2.240845000
6	-2.576786000	0.017436000	-1.684470000
1	-3.235008000	0.020393000	-0.805747000
1	-2.397130000	1.067164000	-1.947987000
6	-3.303101000	-0.694995000	-2.840690000
1	-4.253005000	-0.186970000	-3.052305000
1	-2.695125000	-0.613900000	-3.754005000
6	-3.549272000	-2.177623000	-2.523647000
1	-4.252562000	-2.253789000	-1.680815000
1	-4.029684000	-2.674064000	-3.376438000
6	-2.241221000	-2.894148000	-2.156102000
1	-1.583702000	-2.923081000	-3.037727000
1	-2.441833000	-3.937808000	-1.881983000
6	-1.507396000	-2.184128000	-1.005568000
1	-0.552325000	-2.685798000	-0.800201000
1	-2.105979000	-2.268911000	-0.089140000
6	-1.090702000	0.733987000	1.432836000
1	-0.277640000	1.078725000	2.090356000
6	-2.065135000	1.916219000	1.252669000
1	-1.571680000	2.760851000	0.758395000
1	-2.901327000	1.620797000	0.607195000
6	-2.624553000	2.375413000	2.613265000
1	-1.801955000	2.776728000	3.223676000
1	-3.333391000	3.200407000	2.464258000
6	-3.299285000	1.221693000	3.369830000
1	-3.648763000	1.564346000	4.352212000
1	-4.193574000	0.900755000	2.814654000
6	-2.347673000	0.026497000	3.527936000
1	-2.863263000	-0.809821000	4.017508000
1	-1.513719000	0.308538000	4.187274000
6	-1.783594000	-0.430154000	2.171680000
1	-1.076240000	-1.257045000	2.310692000
1	-2.610201000	-0.815618000	1.558157000

6	0.463307000	1.718578000	-0.916208000
1	-0.428953000	2.310471000	-1.166505000
6	1.234906000	1.422714000	-2.219812000
1	2.069544000	0.745677000	-1.988770000
1	0.595174000	0.896027000	-2.937620000
6	1.772147000	2.706138000	-2.876956000
1	0.926076000	3.326097000	-3.209965000
1	2.340896000	2.445509000	-3.778868000
6	2.644128000	3.518759000	-1.910789000
1	3.551020000	2.944330000	-1.671069000
1	2.978070000	4.449276000	-2.387440000
6	1.883075000	3.824923000	-0.614353000
1	1.040875000	4.497281000	-0.837182000
1	2.529885000	4.360299000	0.092779000
6	1.348797000	2.542191000	0.045584000
1	0.794510000	2.804698000	0.954409000
1	2.193800000	1.915302000	0.365373000
15	-0.093434000	0.111205000	-0.067402000
28	1.551383000	-1.316716000	0.432422000
6	3.258786000	-1.512437000	1.219290000
6	2.879918000	-2.633214000	0.758786000
6	4.199163000	-0.591370000	1.903315000
1	3.753658000	-0.170546000	2.812937000
1	5.125685000	-1.108450000	2.190734000
1	4.472331000	0.251746000	1.257177000
6	3.068012000	-4.083030000	0.494566000
1	4.047608000	-4.435617000	0.846353000
1	2.296921000	-4.680631000	0.996241000
1	2.996944000	-4.304871000	-0.577277000

87

A SCF Done: E(RB3LYP) = -1836.9785731

6	1.930251000	-0.635258000	1.654393000
1	0.981787000	-0.995772000	2.073062000
6	2.714532000	0.051441000	2.789346000
1	3.685647000	0.397239000	2.410339000
1	2.184534000	0.939625000	3.153664000
6	2.953411000	-0.924522000	3.957600000
1	3.540404000	-0.429614000	4.742355000
1	1.984842000	-1.185552000	4.408764000
6	3.658046000	-2.207982000	3.491864000
1	4.678222000	-1.958935000	3.162586000
1	3.763961000	-2.906942000	4.331377000
6	2.903425000	-2.874419000	2.332113000
1	1.926442000	-3.235430000	2.683400000
1	3.453656000	-3.755581000	1.977559000
6	2.678216000	-1.895799000	1.166691000
1	2.108442000	-2.388708000	0.374002000
1	3.650755000	-1.618097000	0.741089000
6	2.740099000	1.538251000	-0.407731000
1	2.320621000	1.936329000	-1.342979000
6	3.186928000	2.748975000	0.439659000
1	2.335443000	3.387067000	0.697460000
1	3.618181000	2.403952000	1.387850000
6	4.231633000	3.596447000	-0.311679000
1	3.759570000	4.040431000	-1.200972000
1	4.550965000	4.434479000	0.321329000
6	5.444472000	2.763141000	-0.747376000
1	6.145282000	3.381563000	-1.322580000
1	5.988915000	2.421632000	0.145701000
6	5.008017000	1.541567000	-1.568017000
1	5.877587000	0.921542000	-1.822248000
1	4.572391000	1.875989000	-2.521246000
6	3.970602000	0.696540000	-0.808707000
1	3.662546000	-0.156062000	-1.422034000
1	4.443706000	0.288515000	0.093972000
6	0.209592000	1.731275000	1.135061000
1	0.874986000	2.324857000	1.775765000
6	-0.862293000	1.096218000	2.044218000
1	-1.500373000	0.427500000	1.455802000
1	-0.391571000	0.480552000	2.818957000
6	-1.734099000	2.171149000	2.719586000
1	-1.114256000	2.756729000	3.415351000
1	-2.507254000	1.682983000	3.326665000
6	-2.377982000	3.117224000	1.696863000
1	-3.090610000	2.553677000	1.078955000
1	-2.953701000	3.897935000	2.210355000
6	-1.315732000	3.751166000	0.789051000
1	-0.671472000	4.414168000	1.386109000
1	-1.791140000	4.380829000	0.026113000
6	-0.448072000	2.679073000	0.108415000
1	0.315733000	3.168424000	-0.510416000
1	-1.074203000	2.084295000	-0.570820000
15	1.293166000	0.453673000	0.232863000
28	0.289099000	-0.823462000	-1.500775000
6	1.056070000	0.206727000	-3.217371000
6	1.647674000	-0.872691000	-3.152563000
6	0.501266000	1.505936000	-3.627241000
1	0.493021000	2.227042000	-2.803412000
1	1.089303000	1.932696000	-4.449860000
1	-0.532912000	1.396276000	-3.974855000
6	2.480504000	-2.062436000	-3.382365000
1	3.148369000	-1.903746000	-4.238599000
1	3.098569000	-2.299310000	-2.509045000
1	1.857951000	-2.939219000	-3.592625000
6	-0.887740000	-2.170317000	-2.240628000
1	-0.332661000	-3.078076000	-2.469470000
1	-1.513427000	-1.822221000	-3.060425000
6	-1.314305000	-1.954428000	-0.887057000
6	-0.952743000	-3.026526000	0.097441000
9	-0.761027000	-2.560147000	1.366958000
9	-1.907500000	-3.992689000	0.212065000
9	0.190789000	-3.679597000	-0.240400000
6	-2.544026000	-1.162876000	-0.608205000

6	-2.965574000	-0.160281000	-1.514645000
6	-3.355992000	-1.371079000	0.527213000
6	-4.121055000	0.575130000	-1.305789000
6	-4.517037000	-0.630676000	0.735398000
6	-4.925738000	0.355456000	-0.173801000
1	-3.092244000	-2.128691000	1.253458000
1	-4.430138000	1.338184000	-2.013381000
1	-5.111081000	-0.837794000	1.620816000
1	-2.364823000	0.046686000	-2.394357000
8	-6.462505000	2.037642000	-0.814827000
6	-6.160897000	1.173370000	-0.001589000
6	-7.050366000	0.914921000	1.206830000
1	-7.412968000	-0.120017000	1.216371000
1	-6.503617000	1.077200000	2.143584000
1	-7.902490000	1.595671000	1.164409000

87

TSA-B SCF Done: E(RB3LYP) = -1836.9693339

6	1.143714000	1.596141000	1.216396000
1	0.090874000	1.438525000	1.483366000
6	1.349296000	3.110283000	0.984822000
1	2.394151000	3.315075000	0.720655000
1	0.738606000	3.471933000	0.151460000
6	0.990861000	3.908084000	2.253398000
1	1.156958000	4.978172000	2.073297000
1	-0.082666000	3.788363000	2.460271000
6	1.798077000	3.437752000	3.471279000
1	2.861628000	3.670532000	3.310738000
1	1.489931000	3.990428000	4.367944000
6	1.637157000	1.927318000	3.692013000
1	0.602110000	1.702784000	3.980105000
1	2.272652000	1.593319000	4.522344000
6	1.987899000	1.129624000	2.423793000
1	1.844533000	0.060183000	2.604780000
1	3.052440000	1.280868000	2.201836000
6	3.172597000	0.264049000	-0.715511000
1	3.122790000	-0.175128000	-1.721488000
6	3.989695000	1.567674000	-0.824465000
1	3.487197000	2.293776000	-1.474713000
1	4.073701000	2.034137000	0.165152000
6	5.407250000	1.287762000	-1.357590000
1	5.337903000	0.914095000	-2.390046000
1	5.977222000	2.224677000	-1.402617000
6	6.142040000	0.254985000	-0.490915000
1	7.132040000	0.040145000	-0.912751000
1	6.313598000	0.679712000	0.509420000
6	5.326082000	-1.038978000	-0.356874000
1	5.838289000	-1.746814000	0.307206000
1	5.260124000	-1.526579000	-1.341227000
6	3.906541000	-0.766139000	0.172228000
1	3.330966000	-1.696989000	0.217232000
1	3.978439000	-0.393461000	1.200878000
6	0.632460000	1.462283000	-1.724874000
1	1.188140000	2.410111000	-1.735837000
6	-0.864150000	1.770441000	-1.501520000
1	-1.417672000	0.825649000	-1.445433000
1	-1.024014000	2.275720000	-0.542399000
6	-1.444249000	2.628052000	-2.639080000
1	-0.961432000	3.617013000	-2.636941000
1	-2.512449000	2.799077000	-2.457655000
6	-1.235635000	1.962595000	-4.005994000
1	-1.823923000	1.034548000	-4.047784000
1	-1.612535000	2.608921000	-4.808632000
6	0.245660000	1.634753000	-4.237292000
1	0.817850000	2.571248000	-4.315413000
1	0.377753000	1.108751000	-5.191625000
6	0.828017000	0.781119000	-3.095849000
1	1.890865000	0.596968000	-3.292438000
1	0.332713000	-0.199098000	-3.082252000
15	1.329124000	0.438221000	-0.286577000
28	0.329103000	-1.521395000	-0.119242000
6	1.138959000	-2.655978000	-1.382002000
6	0.278360000	-3.479168000	-0.859476000
6	2.156850000	-2.797530000	-2.464470000
1	3.171180000	-2.640724000	-2.078103000
1	2.129501000	-3.794679000	-2.927231000
1	2.001765000	-2.056712000	-3.257702000
6	-0.093077000	-4.911064000	-1.075645000
1	0.559318000	-5.345729000	-1.841791000
1	0.014799000	-5.507044000	-0.160305000
1	-1.131808000	-5.022250000	-1.413248000
6	-0.947048000	-3.028499000	0.606750000
1	-0.515042000	-3.734187000	1.312365000
1	-1.804915000	-3.452744000	0.091450000
6	-1.197293000	-1.682278000	1.173292000
6	-0.796887000	-1.574923000	2.610218000
9	-0.670873000	-0.288193000	3.051717000
9	-1.683009000	-2.156615000	3.469553000
9	0.401432000	-2.174717000	2.853817000
6	-2.434368000	-0.979683000	0.741957000
6	-2.959402000	-1.204267000	-0.555686000
6	-3.148464000	-0.079061000	1.562822000
6	-4.111847000	-0.574519000	-0.998366000
6	-4.304982000	0.551744000	1.114422000
6	-4.812268000	0.321539000	-0.172884000
1	-2.807798000	0.126343000	2.568969000
1	-4.496215000	-0.758741000	-1.996817000
1	-4.818116000	1.229537000	1.790789000
1	-2.437304000	-1.870736000	-1.235929000
8	-6.433119000	0.754967000	-1.842322000
6	-6.042864000	0.973146000	-0.701940000
6	-6.817825000	1.922974000	0.202007000
1	-6.196275000	2.772226000	0.510932000
1	-7.686659000	2.293310000	-0.345135000

1 -7.152881000 1.415824000 1.114787000

87
B SCF Done: E(RB3LYP) = -1836.9988871

6	0.558568000	-1.828161000	-1.113189000
1	-0.480097000	-1.852204000	-0.748964000
6	1.066134000	-3.284844000	-1.149933000
1	2.095284000	-3.309497000	-1.528924000
1	1.090390000	-3.720779000	-0.145062000
6	0.177022000	-4.149579000	-2.063317000
1	0.566786000	-5.175090000	-2.094914000
1	-0.831396000	-4.211906000	-1.628497000
6	0.084477000	-3.567901000	-3.481331000
1	1.073901000	-3.622711000	-3.959470000
1	-0.592558000	-4.173536000	-4.096993000
6	-0.381706000	-2.104683000	-3.455087000
1	-1.419157000	-2.056381000	-3.093008000
1	-0.383963000	-1.686183000	-4.469482000
6	0.506108000	-1.242099000	-2.541082000
1	0.136443000	-0.210953000	-2.523538000
1	1.518834000	-1.208163000	-2.963781000
6	3.190486000	-0.366051000	-0.220774000
1	3.494241000	0.218661000	0.659888000
6	4.113645000	-1.598287000	-0.293599000
1	3.973436000	-2.249005000	0.577695000
1	3.862052000	-2.196256000	-1.178765000
6	5.589601000	-1.167375000	-0.390208000
1	5.877190000	-0.656193000	0.540364000
1	6.229977000	-2.055267000	-0.468964000
6	5.829440000	-0.228184000	-1.582278000
1	6.875368000	0.103082000	-1.599014000
1	5.662401000	-0.783146000	-2.517483000
6	4.885827000	0.983314000	-1.541141000
1	5.030372000	1.609616000	-2.430367000
1	5.134332000	1.610723000	-0.672487000
6	3.413210000	0.549000000	-1.445380000
1	2.757604000	1.425626000	-1.390401000
1	3.142768000	0.012728000	-2.363806000
6	1.335519000	-1.648152000	1.755737000
1	1.925378000	-2.558876000	1.579970000
6	-0.103310000	-2.066517000	2.131488000
1	-0.725792000	-1.166900000	2.229808000
1	-0.556011000	-2.667815000	1.335160000
6	-0.142337000	-2.863653000	3.447161000
1	0.376870000	-3.823217000	3.304604000
1	-1.183245000	-3.106029000	3.694516000
6	0.519756000	-2.096643000	4.599219000
1	-0.070788000	-1.196211000	4.823688000
1	0.521356000	-2.705840000	5.511810000
6	1.950559000	-1.682361000	4.230905000
1	2.573554000	-2.582129000	4.117914000
1	2.399485000	-1.090961000	5.038991000
6	1.985068000	-0.873108000	2.922586000
1	3.023407000	-0.611535000	2.685661000
1	1.444603000	0.072032000	3.069061000
15	1.349995000	-0.638268000	0.148018000
28	0.016902000	1.287330000	0.120498000
6	0.707136000	2.753830000	1.098612000
6	-0.179795000	3.719936000	1.424689000
6	2.169329000	2.756551000	1.462570000
1	2.796431000	2.760457000	0.561462000
1	2.451772000	3.640953000	2.049150000
1	2.457899000	1.876323000	2.049161000
6	0.062392000	4.935597000	2.288639000
1	1.058567000	4.958898000	2.737346000
1	-0.064452000	5.859041000	1.704843000
1	-0.670244000	4.983337000	3.107471000
6	-1.557339000	3.572324000	0.835009000
1	-1.902145000	4.523086000	0.398418000
1	-2.301737000	3.336256000	1.606796000
6	-1.496814000	2.460997000	-0.232464000
6	-1.328797000	3.054301000	-1.608272000
9	-0.821890000	2.161836000	-2.523562000
9	-2.498282000	3.499498000	-2.153213000
9	-0.486332000	4.108878000	-1.617788000
6	-2.439143000	1.315370000	-0.131943000
6	-2.842548000	0.839279000	1.146767000
6	-2.888058000	0.567032000	-1.249661000
6	-3.649467000	-0.275808000	1.292015000
6	-3.703752000	-0.552534000	-1.096734000
6	-4.100838000	-0.997660000	0.171306000
1	-2.623730000	0.878814000	-2.251274000
1	-3.950000000	-0.619049000	2.276872000
1	-4.038066000	-1.073523000	-1.989404000
1	-2.499544000	1.357654000	2.036020000
6	-4.968235000	-2.191742000	0.389697000
8	-5.253389000	-2.561645000	1.521316000
6	-5.496828000	-2.947974000	-0.821092000
1	-6.092807000	-2.292763000	-1.467694000
1	-6.118699000	-3.774182000	-0.471994000
1	-4.675695000	-3.346263000	-1.429555000

87
TSB-C SCF Done: E(RB3LYP) = -1836.9787085

6	-2.669221000	0.001634000	1.485086000
1	-1.924970000	-0.174216000	2.270418000
6	-3.918405000	-0.845756000	1.793333000
1	-4.683103000	-0.666433000	1.026390000
1	-3.685910000	-1.917408000	1.763023000
6	-4.505538000	-0.477570000	3.168405000
1	-5.407521000	-1.073742000	3.358212000
1	-3.781726000	-0.744343000	3.952353000
6	-4.824042000	1.021804000	3.258654000
1	-5.633324000	1.261722000	2.552579000

1	-5.199937000	1.270186000	4.259316000
6	-3.590770000	1.873640000	2.925544000
1	-2.823191000	1.721727000	3.698016000
1	-3.847577000	2.940497000	2.945258000
6	-2.993982000	1.510660000	1.553481000
1	-2.082706000	2.091693000	1.384920000
1	-3.708321000	1.787606000	0.767649000
6	-2.833180000	-0.687234000	-1.547590000
1	-2.120626000	-0.831718000	-2.368487000
6	-3.781399000	-1.906829000	-1.564606000
1	-3.225417000	-2.836375000	-1.399971000
1	-4.517216000	-1.831538000	-0.755310000
6	-4.524303000	-1.997277000	-2.911442000
1	-3.796307000	-2.197577000	-3.711319000
1	-5.211275000	-2.853064000	-2.894116000
6	-5.288248000	-0.704998000	-3.233239000
1	-5.771905000	-0.784352000	-4.214931000
1	-6.094579000	-0.568277000	-2.497234000
6	-4.356551000	0.514679000	-3.193435000
1	-4.925189000	1.436901000	-3.367714000
1	-3.622876000	0.440577000	-4.009503000
6	-3.612767000	0.611304000	-1.850148000
1	-2.933433000	1.472090000	-1.852874000
1	-4.347424000	0.790720000	-1.054101000
6	-1.078930000	-2.249487000	0.297691000
1	-1.965631000	-2.897836000	0.320479000
6	-0.361678000	-2.379006000	1.659073000
1	0.462210000	-1.657892000	1.711376000
1	-1.045403000	-2.123995000	2.476383000
6	0.175259000	-3.804374000	1.879026000
1	-0.670773000	-4.503635000	1.958592000
1	0.706102000	-3.848516000	2.838594000
6	1.094917000	-4.254006000	0.735931000
1	1.993935000	-3.620377000	0.722267000
1	1.436464000	-5.283410000	0.903024000
6	0.378652000	-4.141614000	-0.615961000
1	-0.457669000	-4.856040000	-0.646074000
1	1.055387000	-4.417529000	-1.434449000
6	-0.156318000	-2.719070000	-0.850463000
1	-0.684293000	-2.680695000	-1.811694000
1	0.689382000	-2.021425000	-0.929463000
15	-1.677127000	-0.483684000	-0.055712000
28	0.132700000	0.918940000	-0.068382000
6	0.360971000	1.447632000	-1.889812000
6	1.003737000	2.634875000	-1.966231000
6	-0.079321000	0.683503000	-3.115594000
1	-1.138410000	0.845346000	-3.356751000
1	0.495304000	0.961388000	-4.008150000
1	0.059378000	-0.395596000	-2.980402000
6	1.261741000	3.450997000	-3.213762000
1	0.816414000	3.016694000	-4.111808000
1	0.851005000	4.465625000	-3.103227000

1	2.340046000	3.573913000	-3.397784000
6	1.534879000	3.238087000	-0.679777000
1	0.835984000	3.981340000	-0.277441000
1	2.469255000	3.792270000	-0.865484000
6	1.785040000	2.138741000	0.361199000
6	1.277872000	2.337290000	1.654523000
9	0.147962000	0.835218000	1.886283000
9	1.937719000	2.069558000	2.762066000
9	0.379505000	3.273864000	1.891392000
6	2.960268000	1.233023000	0.206499000
6	3.876367000	1.409647000	-0.851638000
6	3.188506000	0.147828000	1.082000000
6	4.966069000	0.564854000	-1.012230000
6	4.282574000	-0.693706000	0.919584000
6	5.195587000	-0.501995000	-0.129731000
1	2.489677000	-0.043262000	1.886945000
1	5.666667000	0.714938000	-1.827620000
1	4.417582000	-1.511929000	1.621042000
1	3.731169000	2.212054000	-1.565225000
6	6.385011000	-1.374635000	-0.352044000
8	7.149807000	-1.173470000	-1.286351000
6	6.637664000	-2.529090000	0.608467000
1	6.765144000	-2.168125000	1.636186000
1	7.542922000	-3.051909000	0.294617000
1	5.796094000	-3.232441000	0.612232000

87

C SCF Done: E(RB3LYP) = -1836.9872766

6	1.720223000	1.689840000	1.169074000
1	0.710758000	2.102496000	1.097429000
6	2.742458000	2.830558000	0.999686000
1	3.764785000	2.440232000	1.090624000
1	2.664864000	3.286045000	0.004498000
6	2.532147000	3.908695000	2.079305000
1	3.278338000	4.704847000	1.958175000
1	1.547303000	4.375553000	1.932987000
6	2.608047000	3.312512000	3.492344000
1	3.630924000	2.950285000	3.676417000
1	2.413006000	4.089229000	4.242943000
6	1.619618000	2.148820000	3.657671000
1	0.591435000	2.532438000	3.591194000
1	1.722801000	1.699572000	4.653927000
6	1.814974000	1.067928000	2.579331000
1	1.045676000	0.299842000	2.692557000
1	2.789701000	0.585730000	2.728288000
6	3.414822000	-0.158046000	-0.650941000
1	3.236668000	-1.001302000	-1.326152000
6	4.328596000	0.824960000	-1.418086000
1	3.835440000	1.189566000	-2.325459000
1	4.550040000	1.705150000	-0.803524000
6	5.650892000	0.138396000	-1.812018000
1	5.435624000	-0.667415000	-2.529358000

1	6.296095000	0.856584000	-2.334169000
6	6.379947000	-0.449180000	-0.595516000
1	7.292488000	-0.968627000	-0.914266000
1	6.701255000	0.370305000	0.064726000
6	5.469135000	-1.403152000	0.190116000
1	5.982005000	-1.768217000	1.089050000
1	5.247517000	-2.287260000	-0.425352000
6	4.150043000	-0.718515000	0.586708000
1	3.507327000	-1.420984000	1.131633000
1	4.375050000	0.101527000	1.281067000
6	1.103077000	1.371950000	-1.765210000
1	1.946653000	2.017200000	-2.044819000
6	-0.126891000	2.277188000	-1.537403000
1	-0.947705000	1.683036000	-1.125562000
1	0.103616000	3.036790000	-0.781682000
6	-0.553126000	2.976709000	-2.839544000
1	0.236403000	3.674530000	-3.158252000
1	-1.447330000	3.583413000	-2.648840000
6	-0.820584000	1.973503000	-3.969502000
1	-1.678348000	1.341671000	-3.696616000
1	-1.097832000	2.499437000	-4.891917000
6	0.407250000	1.085458000	-4.210114000
1	1.235328000	1.704074000	-4.587583000
1	0.197851000	0.337780000	-4.985994000
6	0.848524000	0.375975000	-2.919457000
1	1.746250000	-0.223737000	-3.118628000
1	0.067000000	-0.330198000	-2.605555000
15	1.646198000	0.407174000	-0.222606000
28	-0.075019000	-1.071115000	0.288854000
6	0.414807000	-2.786620000	-0.459592000
6	-0.590549000	-3.690268000	-0.497930000
6	1.770424000	-3.104729000	-1.041942000
1	2.567849000	-2.881206000	-0.325195000
1	1.888799000	-4.157904000	-1.322299000
1	1.972726000	-2.511731000	-1.943938000
6	-0.575528000	-5.061456000	-1.143473000
1	0.339779000	-5.276211000	-1.697474000
1	-0.704303000	-5.851907000	-0.388848000
1	-1.415092000	-5.165716000	-1.846199000
6	-1.916537000	-3.386762000	0.165036000
1	-2.172396000	-4.192065000	0.873739000
1	-2.725187000	-3.391224000	-0.575537000
6	-1.940646000	-2.052415000	0.899031000
6	-1.131843000	-1.962605000	2.006639000
9	-0.777748000	0.546795000	0.799941000
9	-1.193368000	-1.021094000	2.940766000
9	-0.441980000	-2.997800000	2.480053000
6	-3.022241000	-1.075816000	0.599720000
6	-3.416189000	-0.876809000	-0.737869000
6	-3.718973000	-0.387592000	1.605097000
6	-4.456161000	-0.016631000	-1.056993000
6	-4.767775000	0.469767000	1.284164000
6	-5.151782000	0.670950000	-0.049228000
1	-3.442167000	-0.519029000	2.644048000
1	-4.753523000	0.143849000	-2.088169000
1	-5.287740000	0.981581000	2.088156000
1	-2.881681000	-1.389055000	-1.532987000
6	-6.269040000	1.582808000	-0.449764000
8	-6.573174000	1.718370000	-1.626373000
6	-7.021690000	2.341673000	0.632554000
1	-7.490442000	1.652569000	1.345476000
1	-7.793635000	2.950818000	0.159080000
1	-6.345746000	2.990604000	1.202297000

87

TSC-C1 SCF Done: E(RB3LYP) = -1836.9644861

6	-1.364826000	-2.158161000	0.186941000
1	-0.466923000	-2.295335000	-0.424517000
6	-2.406600000	-3.225475000	-0.195140000
1	-3.300286000	-3.118926000	0.434441000
1	-2.735921000	-3.104824000	-1.234687000
6	-1.827876000	-4.638699000	0.007406000
1	-2.585519000	-5.391756000	-0.245761000
1	-0.991616000	-4.785958000	-0.691274000
6	-1.330586000	-4.843745000	1.446374000
1	-2.191202000	-4.818894000	2.131949000
1	-0.877511000	-5.837787000	1.552324000
6	-0.327638000	-3.753802000	1.854580000
1	0.585811000	-3.853291000	1.250984000
1	-0.028993000	-3.885497000	2.902709000
6	-0.906233000	-2.342436000	1.649889000
1	-0.154321000	-1.588738000	1.904569000
1	-1.749056000	-2.201409000	2.339159000
6	-3.500785000	0.098621000	0.365794000
1	-3.541943000	1.176787000	0.153532000
6	-4.720505000	-0.530030000	-0.343846000
1	-4.667832000	-0.373827000	-1.426447000
1	-4.733246000	-1.614453000	-0.182004000
6	-6.034533000	0.077321000	0.184175000
1	-6.072548000	1.142069000	-0.090497000
1	-6.887246000	-0.402447000	-0.313205000
6	-6.160462000	-0.056796000	1.708193000
1	-7.080849000	0.427858000	2.057653000
1	-6.248137000	-1.121284000	1.971664000
6	-4.938202000	0.539149000	2.421405000
1	-5.012782000	0.380565000	3.504789000
1	-4.918371000	1.627924000	2.265205000
6	-3.629583000	-0.073724000	1.894603000
1	-2.767635000	0.369084000	2.407483000
1	-3.621027000	-1.144092000	2.137735000
6	-1.964963000	-0.347563000	-2.139213000
1	-2.893421000	-0.893945000	-2.353761000
6	-0.822583000	-1.045067000	-2.908378000
1	0.134657000	-0.596425000	-2.627841000

1	-0.758139000	-2.098390000	-2.612874000
6	-1.045273000	-0.963048000	-4.428318000
1	-1.943909000	-1.537933000	-4.700297000
1	-0.202711000	-1.440128000	-4.944858000
6	-1.213352000	0.485172000	-4.907657000
1	-0.274752000	1.033898000	-4.740225000
1	-1.404560000	0.512057000	-5.988092000
6	-2.349760000	1.182814000	-4.148366000
1	-3.306457000	0.703664000	-4.405207000
1	-2.433931000	2.232551000	-4.458481000
6	-2.138118000	1.109009000	-2.626881000
1	-2.979658000	1.594301000	-2.115198000
1	-1.238290000	1.678979000	-2.355919000
15	-1.755076000	-0.360328000	-0.250915000
28	0.178404000	0.852652000	0.280073000
6	-0.399638000	2.641340000	0.847644000
6	0.384207000	3.491381000	0.146447000
6	-1.645214000	3.008386000	1.600666000
1	-1.590181000	2.617640000	2.624048000
1	-1.774893000	4.094778000	1.679928000
1	-2.551099000	2.593892000	1.150096000
6	0.023234000	4.878868000	-0.306154000
1	-1.005626000	5.153232000	-0.060268000
1	0.699052000	5.621121000	0.142912000
1	0.144209000	4.967331000	-1.394738000
6	1.808188000	3.029124000	-0.127394000
1	2.512342000	3.817466000	0.192423000
1	1.989989000	2.870978000	-1.198914000
6	1.993153000	1.729472000	0.648348000
6	1.248117000	1.773074000	1.867538000
9	0.919278000	-0.587100000	-0.540690000
9	1.230135000	0.681555000	2.674459000
9	1.264756000	2.824705000	2.726697000
6	3.180320000	0.879442000	0.419409000
6	4.195686000	1.338801000	-0.446803000
6	3.371461000	-0.375990000	1.032620000
6	5.343321000	0.592431000	-0.678180000
6	4.522989000	-1.117649000	0.802571000
6	5.530193000	-0.650287000	-0.056941000
1	2.607296000	-0.781492000	1.679457000
1	6.120986000	0.959432000	-1.340251000
1	4.626418000	-2.079861000	1.295146000
1	4.096358000	2.301669000	-0.935512000
6	6.784023000	-1.410980000	-0.341002000
8	7.637026000	-0.959534000	-1.093349000
6	6.986588000	-2.765314000	0.323216000
1	6.974669000	-2.675703000	1.416199000
1	7.948857000	-3.170045000	0.004506000
1	6.188544000	-3.464178000	0.044934000

87

C1 SCF Done: E(RB3LYP) = -1836.0325056

6	-1.814889000	1.232073000	-1.660628000
1	-0.888412000	1.813149000	-1.580176000
6	-2.978184000	2.178558000	-2.011424000
1	-3.912969000	1.606547000	-2.095556000
1	-3.133977000	2.919890000	-1.217880000
6	-2.712625000	2.896973000	-3.347656000
1	-3.564069000	3.542937000	-3.598489000
1	-1.840946000	3.557062000	-3.230896000
6	-2.444068000	1.899537000	-4.484105000
1	-3.358563000	1.318870000	-4.678845000
1	-2.212844000	2.436989000	-5.412559000
6	-1.301132000	0.937879000	-4.124598000
1	-0.361163000	1.501733000	-4.040887000
1	-1.155399000	0.202003000	-4.925819000
6	-1.567241000	0.211554000	-2.794208000
1	-0.712739000	-0.426548000	-2.541751000
1	-2.437269000	-0.445734000	-2.918268000
6	-3.514608000	-0.392445000	0.401583000
1	-3.283381000	-1.016426000	1.278517000
6	-4.701703000	0.514837000	0.793139000
1	-4.437363000	1.164390000	1.633713000
1	-4.958652000	1.174438000	-0.046026000
6	-5.935951000	-0.320313000	1.184020000
1	-5.711036000	-0.879779000	2.104343000
1	-6.771375000	0.349518000	1.424571000
6	-6.341712000	-1.306848000	0.080448000
1	-7.186555000	-1.923281000	0.412432000
1	-6.690432000	-0.744934000	-0.798598000
6	-5.158543000	-2.194711000	-0.329392000
1	-5.444354000	-2.853775000	-1.159091000
1	-4.884406000	-2.850603000	0.510765000
6	-3.939751000	-1.348112000	-0.734578000
1	-3.108827000	-1.998200000	-1.028751000
1	-4.204073000	-0.758543000	-1.621856000
6	-1.814765000	1.913695000	1.262061000
1	-2.801515000	2.391575000	1.200400000
6	-0.750079000	2.981636000	0.928335000
1	0.231047000	2.503268000	0.855777000
1	-0.949817000	3.414630000	-0.058556000
6	-0.741198000	4.109351000	1.975090000
1	-1.691393000	4.663309000	1.927784000
1	0.050398000	4.827153000	1.726071000
6	-0.544657000	3.573056000	3.399269000
1	0.452482000	3.116143000	3.479971000
1	-0.574807000	4.394447000	4.126354000
6	-1.610466000	2.522352000	3.737743000
1	-2.600833000	3.001114000	3.762641000
1	-1.439544000	2.106754000	4.739366000
6	-1.621364000	1.386241000	2.701575000
1	-2.403959000	0.659756000	2.958917000
1	-0.663267000	0.848227000	2.749354000
15	-1.831451000	0.445065000	0.055859000

28	0.235715000	-0.687129000	0.231559000
6	0.131052000	-2.874402000	0.273774000
6	0.128836000	-2.395940000	1.571407000
6	-0.890841000	-3.710770000	-0.430663000
1	-1.007468000	-3.394019000	-1.471395000
1	-0.548044000	-4.754282000	-0.447116000
1	-1.866034000	-3.682091000	0.061445000
6	-0.940759000	-2.582210000	2.604192000
1	-1.912937000	-2.823514000	2.165061000
1	-0.662964000	-3.406963000	3.275911000
1	-1.051655000	-1.686835000	3.224837000
6	1.527733000	-1.903073000	1.937111000
1	2.111369000	-2.695618000	2.439546000
1	1.503334000	-1.037522000	2.609058000
6	2.012671000	-1.560038000	0.524710000
6	1.522302000	-2.712285000	-0.296015000
9	0.891038000	0.854339000	-0.399996000
9	1.484804000	-2.484564000	-1.660870000
9	2.249966000	-3.896387000	-0.175870000
6	3.279085000	-0.860533000	0.278777000
6	4.067991000	-0.397202000	1.352998000
6	3.740801000	-0.604037000	-1.029006000
6	5.259586000	0.279467000	1.133436000
6	4.936331000	0.068871000	-1.246007000
6	5.717729000	0.523770000	-0.170585000
1	3.150403000	-0.934127000	-1.874212000
1	5.865637000	0.627411000	1.963864000
1	5.257238000	0.244775000	-2.268462000
1	3.747728000	-0.580442000	2.374452000
6	7.009127000	1.251004000	-0.348995000
8	7.660186000	1.627622000	0.616872000
6	7.510352000	1.519921000	-1.761171000
1	7.654313000	0.584202000	-2.314720000
1	8.461053000	2.052514000	-1.698474000
1	6.791678000	2.125445000	-2.326472000

87

TSC1-D SCF Done: E(RB3LYP) = -1836.0184604

6	-2.595439000	1.059577000	1.526911000
1	-2.603763000	0.108611000	2.083852000
6	-4.041441000	1.594183000	1.505513000
1	-4.065316000	2.560690000	0.985802000
1	-4.711561000	0.923903000	0.954081000
6	-4.568540000	1.778552000	2.941922000
1	-5.585222000	2.191523000	2.913134000
1	-4.647291000	0.791312000	3.421468000
6	-3.649709000	2.679351000	3.782034000
1	-3.685866000	3.702963000	3.380658000
1	-4.019712000	2.736697000	4.813740000
6	-2.194919000	2.184337000	3.756908000
1	-2.126617000	1.217615000	4.278700000
1	-1.547806000	2.880121000	4.305679000
6	-1.682324000	2.024901000	2.316488000
1	-0.643110000	1.685992000	2.282994000
1	-1.700511000	3.010246000	1.832580000
6	-1.634107000	1.927553000	-1.344460000
1	-1.189143000	1.393928000	-2.199164000
6	-2.920783000	2.614630000	-1.846135000
1	-3.672966000	1.883928000	-2.166385000
1	-3.370520000	3.191805000	-1.027444000
6	-2.604007000	3.564370000	-3.017452000
1	-2.258803000	2.967979000	-3.875219000
1	-3.522551000	4.071090000	-3.341129000
6	-1.525624000	4.594934000	-2.648676000
1	-1.277810000	5.209663000	-3.523554000
1	-1.929221000	5.281635000	-1.889503000
6	-0.265205000	3.916846000	-2.090378000
1	0.463128000	4.672872000	-1.771229000
1	0.221447000	3.332093000	-2.885323000
6	-0.597982000	2.989549000	-0.909989000
1	0.296196000	2.510080000	-0.504064000
1	-1.022371000	3.596870000	-0.099398000
6	-3.180965000	-0.576176000	-0.905382000
1	-4.074435000	0.049834000	-1.032752000
6	-3.556505000	-1.767265000	0.002720000
1	-2.654138000	-2.361564000	0.205603000
1	-3.921782000	-1.412883000	0.973706000
6	-4.625016000	-2.665786000	-0.645122000
1	-5.562700000	-2.098623000	-0.739026000
1	-4.841076000	-3.516552000	0.013894000
6	-4.190117000	-3.157705000	-2.032223000
1	-3.323217000	-3.825819000	-1.925482000
1	-4.988373000	-3.753281000	-2.492615000
6	-3.812739000	-1.980342000	-2.941346000
1	-4.710980000	-1.380311000	-3.148904000
1	-3.451521000	-2.345801000	-3.910904000
6	-2.743220000	-1.078741000	-2.299370000
1	-2.536055000	-0.234811000	-2.966869000
1	-1.799711000	-1.635876000	-2.205033000
15	-1.833522000	0.494338000	-0.113985000
28	0.253491000	-0.525902000	0.262751000
6	0.420348000	-3.273537000	0.206285000
6	0.409031000	-2.971865000	1.530522000
6	-0.383555000	-4.279449000	-0.560213000
1	-0.751157000	-3.859631000	-1.502212000
1	0.252469000	-5.135357000	-0.821762000
1	-1.236820000	-4.654370000	0.011340000
6	-0.509283000	-3.504105000	2.585313000
1	-1.318102000	-4.117200000	2.178446000
1	0.055731000	-4.117092000	3.300712000
1	-0.951282000	-2.681056000	3.160632000
6	1.577178000	-2.069502000	1.895366000
1	2.384924000	-2.673701000	2.347156000
1	1.306392000	-1.304354000	2.632053000

6	1.954971000	-1.485439000	0.526108000
6	1.586918000	-2.577775000	-0.445552000
9	0.937388000	1.002221000	0.895423000
9	1.202052000	-2.067426000	-1.699780000
9	2.590472000	-3.478246000	-0.758802000
6	3.200946000	-0.723174000	0.351585000
6	3.808532000	-0.077287000	1.448545000
6	3.819052000	-0.590945000	-0.909197000
6	4.980682000	0.647782000	1.296981000
6	4.995304000	0.133952000	-1.058498000
6	5.600241000	0.763967000	0.041728000
1	3.373095000	-1.065436000	-1.775874000
1	5.445965000	1.139940000	2.145016000
1	5.444701000	0.203265000	-2.044844000
1	3.346961000	-0.140649000	2.428471000
6	6.867527000	1.544212000	-0.059920000
8	7.367601000	2.070455000	0.926038000
6	7.540487000	1.677662000	-1.419791000
1	7.805473000	0.694786000	-1.827933000
1	8.445942000	2.275957000	-1.303059000
1	6.875727000	2.162976000	-2.144500000

87

D SCF Done: E(RB3LYP) = -1836.0434026

6	-1.073078000	-2.191869000	-1.002807000
1	-0.634780000	-1.694848000	-1.879008000
6	-2.107992000	-3.229882000	-1.480463000
1	-2.546460000	-3.747816000	-0.618225000
1	-2.937297000	-2.748507000	-2.011567000
6	-1.438885000	-4.272795000	-2.396174000
1	-2.180553000	-5.016695000	-2.714854000
1	-1.081810000	-3.773098000	-3.308320000
6	-0.256980000	-4.960946000	-1.697384000
1	-0.635598000	-5.557305000	-0.853397000
1	0.230867000	-5.665454000	-2.382815000
6	0.759998000	-3.934568000	-1.175760000
1	1.231741000	-3.420176000	-2.024645000
1	1.565873000	-4.440407000	-0.628888000
6	0.092821000	-2.887056000	-0.267355000
1	0.833827000	-2.146484000	0.056265000
1	-0.280939000	-3.388062000	0.636832000
6	-2.467518000	-1.085857000	1.592555000
1	-2.941784000	-0.129525000	1.862683000
6	-3.566039000	-2.168013000	1.605889000
1	-4.330385000	-1.964996000	0.846061000
1	-3.122527000	-3.140171000	1.355678000
6	-4.225054000	-2.267141000	2.993773000
1	-4.754572000	-1.327410000	3.210143000
1	-4.984765000	-3.059249000	2.987539000
6	-3.185667000	-2.531844000	4.092723000
1	-3.670167000	-2.552339000	5.077136000
1	-2.746302000	-3.528918000	3.940119000
6	-2.067884000	-1.478273000	4.070716000
1	-1.305736000	-1.714865000	4.824012000
1	-2.485992000	-0.498914000	4.346994000
6	-1.409843000	-1.369467000	2.683734000
1	-0.652680000	-0.578762000	2.688686000
1	-0.889151000	-2.309975000	2.460704000
6	-3.116462000	0.026507000	-1.105408000
1	-3.953691000	-0.683948000	-1.037475000
6	-2.727951000	0.194224000	-2.592501000
1	-1.813188000	0.793590000	-2.655059000
1	-2.479592000	-0.777858000	-3.032618000
6	-3.864049000	0.837136000	-3.406476000
1	-4.725178000	0.152273000	-3.437839000
1	-3.535651000	0.971286000	-4.444979000
6	-4.312523000	2.177882000	-2.810612000
1	-3.486973000	2.901412000	-2.882805000
1	-5.147544000	2.594290000	-3.388374000
6	-4.711326000	2.012578000	-1.338665000
1	-5.605599000	1.375157000	-1.272315000
1	-4.985576000	2.981372000	-0.901472000
6	-3.575043000	1.381129000	-0.516774000
1	-3.901031000	1.265818000	0.524235000
1	-2.714993000	2.066660000	-0.502175000
15	-1.683492000	-0.665915000	-0.078864000
28	0.098736000	0.812563000	-0.062406000
6	0.506451000	3.874028000	1.001460000
6	0.718965000	4.187660000	-0.294567000
6	-0.217837000	4.616564000	2.083440000
1	0.447779000	4.813924000	2.932460000
1	-0.609463000	5.573297000	1.728633000
1	-1.058295000	4.024476000	2.466439000
6	0.239617000	5.398175000	-1.033143000
1	-0.307127000	6.096736000	-0.393712000
1	1.083228000	5.936503000	-1.484661000
1	-0.420944000	5.103775000	-1.859913000
6	1.549922000	3.136961000	-0.999419000
1	2.525123000	3.565600000	-1.282624000
1	1.077006000	2.783759000	-1.924051000
6	1.696205000	2.016182000	0.031536000
6	1.186384000	2.589613000	1.293830000
9	0.268119000	0.484507000	-1.798981000
9	0.087830000	1.541521000	1.756603000
9	1.936429000	2.600788000	2.424654000
6	2.836131000	1.083004000	-0.011845000
6	3.522608000	0.869320000	-1.229341000
6	3.245319000	0.317911000	1.104907000
6	4.562629000	-0.042239000	-1.320764000
6	4.289698000	-0.594399000	1.007490000
6	4.971066000	-0.792833000	-0.204858000
1	2.744874000	0.445375000	2.057775000
1	5.083266000	-0.198274000	-2.260347000
1	4.574984000	-1.154263000	1.893587000

1	3.215738000	1.414576000	-2.115590000
6	6.096055000	-1.756702000	-0.362434000
8	6.664851000	-1.897077000	-1.437980000
6	6.535452000	-2.576117000	0.844623000
1	6.870612000	-1.927225000	1.662878000
1	7.357141000	-3.228022000	0.542387000
1	5.710716000	-3.187326000	1.230624000

87

TSD-E SCF Done: E(RB3LYP) = -1836.0433547

6	-1.059486000	-2.208571000	-0.972414000
1	-0.607919000	-1.729060000	-1.851498000
6	-2.090651000	-3.252171000	-1.445688000
1	-2.544588000	-3.751778000	-0.580563000
1	-2.909576000	-2.778031000	-1.999011000
6	-1.411363000	-4.314706000	-2.330863000
1	-2.150527000	-5.062735000	-2.645636000
1	-1.039560000	-3.834031000	-3.247355000
6	-0.241705000	-4.992095000	-1.601742000
1	-0.634279000	-5.569709000	-0.751132000
1	0.253174000	-5.711982000	-2.265803000
6	0.771256000	-3.958405000	-1.086767000
1	1.256061000	-3.462185000	-1.939117000
1	1.568077000	-4.455581000	-0.519085000
6	0.094603000	-2.892169000	-0.207770000
1	0.832378000	-2.146729000	0.112064000
1	-0.292007000	-3.375582000	0.700438000
6	-2.496120000	-1.047148000	1.577410000
1	-2.957295000	-0.078624000	1.825714000
6	-3.613784000	-2.109585000	1.588867000
1	-4.362599000	-1.904600000	0.814322000
1	-3.184416000	-3.093313000	1.360190000
6	-4.296006000	-2.175735000	2.967486000
1	-4.812100000	-1.223611000	3.160807000
1	-5.069414000	-2.954508000	2.961149000
6	-3.278753000	-2.440950000	4.087054000
1	-3.779318000	-2.437816000	5.063625000
1	-2.854458000	-3.447679000	3.956727000
6	-2.142794000	-1.407172000	4.066448000
1	-1.397133000	-1.644573000	4.835821000
1	-2.548029000	-0.416402000	4.320291000
6	-1.460257000	-1.331825000	2.689141000
1	-0.691283000	-0.553347000	2.692931000
1	-0.953756000	-2.285224000	2.489382000
6	-3.091682000	0.014829000	-1.153949000
1	-3.931631000	-0.692767000	-1.089844000
6	-2.674059000	0.155742000	-2.635921000
1	-1.756476000	0.751425000	-2.689870000
1	-2.420042000	-0.824465000	-3.054494000
6	-3.792150000	0.787884000	-3.482505000
1	-4.654661000	0.105021000	-3.519586000
1	-3.443012000	0.903756000	-4.516453000
6	-4.247858000	2.139715000	-2.918083000
1	-3.418650000	2.859517000	-2.986170000
1	-5.069873000	2.548822000	-3.519243000
6	-4.676320000	2.000449000	-1.451801000
1	-5.573754000	1.366760000	-1.392742000
1	-4.956299000	2.977209000	-1.036490000
6	-3.558408000	1.379980000	-0.597022000
1	-3.905219000	1.282103000	0.438924000
1	-2.697423000	2.064098000	-0.575847000
15	-1.681684000	-0.665126000	-0.087672000
28	0.094451000	0.805964000	-0.050001000
6	0.512348000	3.869285000	1.014961000
6	0.703318000	4.178072000	-0.287105000
6	-0.206116000	4.607428000	2.103285000
1	0.466983000	4.816024000	2.943594000
1	-0.613927000	5.557902000	1.749858000
1	-1.033080000	4.004118000	2.497230000
6	0.199633000	5.377390000	-1.026986000
1	-0.339631000	6.077022000	-0.382439000
1	1.028849000	5.918444000	-1.501164000
1	-0.475908000	5.069862000	-1.836825000
6	1.533134000	3.127957000	-0.993095000
1	2.501192000	3.562758000	-1.290474000
1	1.053414000	2.763233000	-1.909609000
6	1.708223000	2.019379000	0.047454000
6	1.201421000	2.593852000	1.293072000
9	0.286145000	0.455108000	-1.780426000
9	0.029281000	1.501982000	1.746013000
9	1.898511000	2.554660000	2.447979000
6	2.847740000	1.086562000	-0.003022000
6	3.538480000	0.884130000	-1.219506000
6	3.254451000	0.313865000	1.109188000
6	4.583087000	-0.022244000	-1.313504000
6	4.303351000	-0.592720000	1.009288000
6	4.991088000	-0.778550000	-0.201551000
1	2.745912000	0.428934000	2.059236000
1	5.107772000	-0.169163000	-2.252305000
1	4.586503000	-1.158932000	1.892016000
1	3.233211000	1.434321000	-2.103281000
6	6.121710000	-1.735410000	-0.361226000
8	6.695863000	-1.865212000	-1.435259000
6	6.560349000	-2.560761000	0.842076000
1	6.888369000	-1.915701000	1.666243000
1	7.386888000	-3.206130000	0.539042000
1	5.737249000	-3.179019000	1.220254000

87

E SCF Done: E(RB3LYP) = -1836.0691218

6	-1.444472000	-1.538936000	-1.752999000
1	-1.825470000	-0.671477000	-2.306165000
6	-2.326027000	-2.762975000	-2.069681000
1	-1.951246000	-3.640130000	-1.525147000

1	-3.359111000	-2.602421000	-1.736975000
6	-2.305657000	-3.069074000	-3.579156000
1	-2.913251000	-3.959869000	-3.784315000
1	-2.777409000	-2.235728000	-4.120206000
6	-0.873168000	-3.266112000	-4.096833000
1	-0.446943000	-4.173351000	-3.643062000
1	-0.882880000	-3.435158000	-5.181056000
6	0.017108000	-2.062672000	-3.751967000
1	-0.337614000	-1.177504000	-4.299887000
1	1.047679000	-2.244176000	-4.082031000
6	0.002795000	-1.756477000	-2.244213000
1	0.603768000	-0.865353000	-2.035199000
1	0.466268000	-2.591999000	-1.704901000
6	-1.141267000	-2.156152000	1.332954000
1	-1.130435000	-1.516484000	2.225147000
6	-2.174543000	-3.282339000	1.539006000
1	-3.183436000	-2.874275000	1.672573000
1	-2.210656000	-3.926811000	0.650997000
6	-1.805763000	-4.137937000	2.765636000
1	-1.887009000	-3.518371000	3.670759000
1	-2.532254000	-4.953166000	2.877945000
6	-0.380189000	-4.699380000	2.662425000
1	-0.131145000	-5.266099000	3.568739000
1	-0.332234000	-5.412197000	1.825371000
6	0.643239000	-3.578041000	2.432565000
1	1.649227000	-3.999381000	2.309901000
1	0.680909000	-2.928083000	3.318409000
6	0.288256000	-2.724148000	1.203627000
1	0.994506000	-1.895726000	1.106703000
1	0.365222000	-3.349694000	0.304185000
6	-3.322918000	-0.368262000	0.293517000
1	-3.924735000	-1.288714000	0.300253000
6	-3.849288000	0.556353000	-0.826772000
1	-3.164376000	1.404674000	-0.937341000
1	-3.840133000	0.028882000	-1.787793000
6	-5.280800000	1.039687000	-0.536516000
1	-5.969300000	0.181700000	-0.570381000
1	-5.599789000	1.725824000	-1.331437000
6	-5.393997000	1.718597000	0.834634000
1	-4.793227000	2.640135000	0.835015000
1	-6.431633000	2.019174000	1.027820000
6	-4.891220000	0.787472000	1.945580000
1	-5.556317000	-0.086129000	2.017565000
1	-4.931941000	1.291508000	2.919565000
6	-3.453616000	0.315018000	1.673928000
1	-3.128803000	-0.360068000	2.474351000
1	-2.775187000	1.179229000	1.703490000
15	-1.527715000	-0.877717000	0.006112000
28	-0.201943000	0.879027000	0.176874000
6	0.051156000	4.140243000	0.465291000
6	0.202719000	4.231266000	-0.879974000
6	-0.847652000	4.890019000	1.400934000
1	-0.282329000	5.317255000	2.237651000
1	-1.367536000	5.704968000	0.890192000
1	-1.606188000	4.225782000	1.835317000
6	-0.532697000	5.103228000	-1.844754000
1	-1.170476000	5.834729000	-1.339906000
1	0.159070000	5.648831000	-2.500003000
1	-1.171312000	4.488715000	-2.493548000
6	1.236644000	3.233779000	-1.344786000
1	2.115392000	3.758472000	-1.749160000
1	0.827163000	2.590085000	-2.127532000
6	1.631062000	2.458789000	-0.098587000
6	0.941370000	3.078988000	0.920136000
9	-0.880011000	1.447282000	-1.382723000
9	0.394766000	0.338166000	1.753848000
9	1.116912000	2.884866000	2.230259000
6	2.763759000	1.528677000	-0.041385000
6	3.451024000	1.190326000	-1.227826000
6	3.206878000	0.948562000	1.166556000
6	4.534228000	0.324027000	-1.209045000
6	4.294450000	0.083570000	1.178186000
6	4.979526000	-0.243254000	-0.004318000
1	2.667683000	1.148892000	2.081364000
1	5.061069000	0.068868000	-2.122898000
1	4.606252000	-0.343852000	2.126495000
1	3.127503000	1.611815000	-2.174677000
6	6.152884000	-1.167099000	-0.041978000
8	6.712874000	-1.429336000	-1.098215000
6	6.645779000	-1.781342000	1.260351000
1	6.941013000	-1.005207000	1.976647000
1	7.505111000	-2.418102000	1.042595000
1	5.861058000	-2.381564000	1.736485000

32

F SCF Done: E(RB3LYP) = -755.673991

6	3.537828000	0.450901000	0.000074000
6	3.535770000	-0.905723000	-0.000288000
6	4.676945000	1.424385000	0.000197000
1	4.637880000	2.078211000	0.880434000
1	5.644112000	0.914798000	0.000446000
1	4.638251000	2.077992000	-0.880224000
6	4.697135000	-1.849013000	-0.000446000
1	5.654925000	-1.320453000	-0.000521000
1	4.678779000	-2.506075000	0.880134000
1	4.678624000	-2.506040000	-0.881049000
6	2.102862000	-1.395971000	-0.000261000
1	1.902691000	-2.024999000	0.879434000
1	1.902413000	-2.024714000	-0.880088000
6	1.249956000	-0.142019000	0.000023000
6	2.142988000	0.880288000	0.000218000
9	1.853409000	2.196375000	0.000553000
6	-0.206105000	-0.120352000	0.000017000
6	-0.938658000	-1.328665000	0.000527000

6	-0.939613000	1.087256000	-0.000468000
6	-2.325151000	-1.330624000	0.000501000
6	-2.328661000	1.079774000	-0.000453000
6	-3.048560000	-0.126702000	0.000019000
1	-0.410406000	2.032140000	-0.000887000
1	-2.881939000	-2.262210000	0.000866000
1	-2.854461000	2.029991000	-0.000835000
1	-0.409023000	-2.276835000	0.000974000
6	-4.540482000	-0.190663000	0.000005000
8	-5.122830000	-1.267098000	0.000204000
6	-5.331341000	1.109722000	-0.000325000
1	-5.096333000	1.716445000	0.882579000
1	-6.396130000	0.870066000	0.000112000
1	-5.096916000	1.715526000	-0.884027000

77

TSRe-A' SCF Done: E(RB3LYP) = -1680.9544055

6	2.606448000	-0.145466000	1.618405000
1	1.925246000	0.157324000	2.427924000
6	3.968223000	0.526396000	1.878580000
1	4.674819000	0.233503000	1.090970000
1	3.883839000	1.619733000	1.836992000
6	4.547126000	0.097589000	3.239449000
1	5.527945000	0.567358000	3.389302000
1	3.895512000	0.470552000	4.043456000
6	4.661620000	-1.430341000	3.343473000
1	5.404769000	-1.786120000	2.613971000
1	5.036591000	-1.715793000	4.334604000
6	3.313247000	-2.109885000	3.065132000
1	2.602654000	-1.848643000	3.863266000
1	3.422263000	-3.201401000	3.093176000
6	2.719409000	-1.686095000	1.709622000
1	1.734044000	-2.143755000	1.573563000
1	3.365354000	-2.060428000	0.903673000
6	2.760730000	0.490687000	-1.440797000
1	2.028143000	0.736671000	-2.224100000
6	3.860530000	1.571966000	-1.481348000
1	3.447343000	2.561306000	-1.254249000
1	4.619925000	1.366653000	-0.717639000
6	4.539925000	1.604609000	-2.863756000
1	3.805361000	1.921177000	-3.619029000
1	5.335511000	2.360636000	-2.866729000
6	5.106687000	0.230780000	-3.251192000
1	5.543289000	0.270007000	-4.257110000
1	5.926525000	-0.027981000	-2.564549000
6	4.027190000	-0.859364000	-3.180599000
1	4.461997000	-1.842200000	-3.402512000
1	3.267343000	-0.671509000	-3.953532000
6	3.344975000	-0.891581000	-1.802067000
1	2.555305000	-1.652210000	-1.786016000
1	4.085070000	-1.188207000	-1.046402000
6	1.182611000	2.211122000	0.439721000
1	2.113952000	2.758019000	0.646723000
6	0.267699000	2.317882000	1.680505000
1	-0.628908000	1.703079000	1.515012000
1	0.765595000	1.911953000	2.568455000
6	-0.152124000	3.771862000	1.957637000
1	0.737317000	4.358939000	2.231113000
1	-0.823091000	3.799755000	2.825532000
6	-0.826804000	4.411504000	0.737012000
1	-1.777477000	3.896434000	0.536157000
1	-1.075338000	5.459964000	0.944307000
6	0.073914000	4.310914000	-0.501210000
1	0.974476000	4.924042000	-0.347355000
1	-0.437011000	4.721965000	-1.381120000
6	0.490873000	2.856749000	-0.781986000
1	1.145845000	2.829663000	-1.660655000
1	-0.399550000	2.264691000	-1.036558000
15	1.634552000	0.405388000	0.083781000
28	-0.131697000	-0.880772000	-0.216471000
6	-0.767586000	-1.014784000	-2.036346000
1	-0.064862000	-1.616276000	-2.613608000
1	-1.156021000	-0.157294000	-2.580853000
6	-1.708008000	-1.690305000	-1.152914000
6	-1.290013000	-2.858213000	-0.466369000
9	-2.168576000	-3.639137000	0.127309000
9	-0.271429000	-3.571993000	-0.918229000
9	-0.531768000	-2.332278000	1.165661000
6	-3.009788000	-1.075033000	-0.773285000
6	-3.809522000	-0.464318000	-1.757113000
6	-3.470046000	-1.071422000	0.557550000
6	-5.020026000	0.129667000	-1.424722000
6	-4.686927000	-0.482156000	0.885103000
6	-5.480834000	0.129580000	-0.098446000
1	-2.851679000	-1.516800000	1.329849000
1	-5.640336000	0.596477000	-2.183113000
1	-5.009916000	-0.493844000	1.921786000
1	-3.481315000	-0.474328000	-2.792618000
8	-7.443046000	1.317260000	-0.687633000
6	-6.793838000	0.780421000	0.199325000
6	-7.313770000	0.763953000	1.629331000
1	-6.613232000	1.260952000	2.311266000
1	-8.273808000	1.282362000	1.657487000
1	-7.445477000	-0.263373000	1.989873000

77

A' SCF Done: E(RB3LYP) = -1680.9857709

6	2.863652000	-0.177849000	1.292762000
1	2.198118000	-0.332265000	2.152235000
6	3.951417000	0.845646000	1.667874000
1	4.633933000	0.991443000	0.819629000
1	3.512357000	1.825835000	1.890003000
6	4.763296000	0.350659000	2.879432000
1	5.550060000	1.076851000	3.121589000

1	4.101534000	0.299429000	3.756204000
6	5.374325000	-1.034965000	2.621961000
1	6.127873000	-0.953744000	1.823943000
1	5.904626000	-1.386860000	3.515946000
6	4.301863000	-2.052316000	2.203534000
1	3.615453000	-2.229161000	3.043622000
1	4.765527000	-3.018871000	1.968302000
6	3.489098000	-1.557894000	0.994240000
1	2.701049000	-2.280393000	0.753187000
1	4.152247000	-1.495891000	0.120936000
6	2.343837000	0.862296000	-1.635603000
1	1.443335000	1.085358000	-2.228373000
6	3.225742000	2.128716000	-1.646367000
1	2.710192000	2.970095000	-1.169974000
1	4.140510000	1.950343000	-1.066770000
6	3.610347000	2.515000000	-3.087231000
1	2.701550000	2.803596000	-3.635861000
1	4.259218000	3.399973000	-3.071625000
6	4.301237000	1.358262000	-3.824130000
1	4.518254000	1.644460000	-4.860940000
1	5.270859000	1.153258000	-3.346600000
6	3.444150000	0.084350000	-3.789629000
1	3.976722000	-0.747273000	-4.268190000
1	2.525571000	0.245293000	-4.373081000
6	3.067890000	-0.300024000	-2.348366000
1	2.442420000	-1.199836000	-2.343808000
1	3.985636000	-0.550839000	-1.800737000
6	0.797650000	1.923641000	0.697662000
1	1.556391000	2.717307000	0.643887000
6	0.363327000	1.764502000	2.172953000
1	-0.253842000	0.864753000	2.273613000
1	1.243195000	1.596017000	2.804747000
6	-0.383405000	3.009230000	2.684956000
1	0.311917000	3.861492000	2.725204000
1	-0.717194000	2.831427000	3.715173000
6	-1.574010000	3.380507000	1.791641000
1	-2.322739000	2.575670000	1.827560000
1	-2.065110000	4.287319000	2.167168000
6	-1.119770000	3.580447000	0.340248000
1	-0.443552000	4.446584000	0.285151000
1	-1.977041000	3.810898000	-0.304972000
6	-0.399466000	2.330625000	-0.191975000
1	-0.071497000	2.507393000	-1.224375000
1	-1.113340000	1.496202000	-0.226431000
15	1.577416000	0.335990000	0.015599000
28	0.021021000	-1.300577000	0.006539000
6	-0.548108000	-1.473960000	-1.930140000
1	0.143025000	-2.162047000	-2.411946000
1	-0.791961000	-0.582110000	-2.499638000
6	-1.548829000	-1.993146000	-1.049181000
6	-1.082488000	-3.003317000	-0.188464000
9	-1.828477000	-3.520198000	0.781436000
9	-0.180541000	-3.914559000	-0.602922000
9	0.078921000	-1.353703000	1.817212000
6	-2.815730000	-1.267921000	-0.753412000
6	-3.633071000	-0.870333000	-1.825509000
6	-3.218702000	-0.963722000	0.558946000
6	-4.825866000	-0.196232000	-1.593319000
6	-4.415217000	-0.288276000	0.784796000
6	-5.235356000	0.103388000	-0.285060000
1	-2.573650000	-1.228710000	1.390755000
1	-5.465453000	0.109298000	-2.414922000
1	-4.700672000	-0.060935000	1.807265000
1	-3.331095000	-1.105364000	-2.842247000
8	-7.203291000	1.167448000	-1.060451000
6	-6.531500000	0.832814000	-0.095842000
6	-6.996285000	1.148651000	1.316729000
1	-6.269651000	1.783046000	1.838677000
1	-7.954274000	1.668463000	1.261402000
1	-7.113284000	0.232691000	1.908253000

87

B' SCF Done: E(RB3LYP) = -1836.9517952

6	1.822720000	-0.234886000	1.845007000
1	0.823399000	0.162293000	2.064515000
6	2.806643000	0.267239000	2.918493000
1	3.810397000	-0.133827000	2.719740000
1	2.893297000	1.360257000	2.889979000
6	2.363002000	-0.183307000	4.322835000
1	3.088465000	0.160467000	5.071698000
1	1.405682000	0.299296000	4.567363000
6	2.196684000	-1.708342000	4.399327000
1	3.180273000	-2.185571000	4.272037000
1	1.834866000	-2.001195000	5.393175000
6	1.240742000	-2.221319000	3.311925000
1	0.228837000	-1.837376000	3.504317000
1	1.173272000	-3.316293000	3.348990000
6	1.683118000	-1.773150000	1.907494000
1	0.953099000	-2.116930000	1.164822000
1	2.640619000	-2.254190000	1.666441000
6	3.831169000	0.212889000	-0.522489000
1	3.734907000	0.459478000	-1.592114000
6	4.910837000	1.147398000	0.065604000
1	4.612947000	2.197173000	-0.023456000
1	5.031472000	0.945210000	1.137711000
6	6.264885000	0.953581000	-0.643052000
1	6.169831000	1.275637000	-1.690878000
1	7.017799000	1.607522000	-0.184440000
6	6.731340000	-0.508082000	-0.604409000
1	7.670169000	-0.623530000	-1.160747000
1	6.947223000	-0.792304000	0.436267000
6	5.655120000	-1.445088000	-1.169833000
1	5.975701000	-2.491477000	-1.084509000
1	5.523256000	-1.244277000	-2.243423000

6	4.308442000	-1.252944000	-0.451193000
1	3.556258000	-1.918840000	-0.886341000
1	4.424446000	-1.548042000	0.600130000
6	1.878861000	2.307382000	0.276037000
1	2.766650000	2.646398000	0.827203000
6	0.632908000	2.755594000	1.069293000
1	-0.259136000	2.329855000	0.601842000
1	0.666004000	2.347123000	2.085691000
6	0.538818000	4.289280000	1.151186000
1	1.380832000	4.676548000	1.745293000
1	-0.375848000	4.569096000	1.689035000
6	0.562931000	4.944267000	-0.236276000
1	-0.340055000	4.651872000	-0.792181000
1	0.535032000	6.037582000	-0.144770000
6	1.804712000	4.506862000	-1.024207000
1	2.707040000	4.893560000	-0.527341000
1	1.793925000	4.939421000	-2.033221000
6	1.894666000	2.974279000	-1.117268000
1	2.797682000	2.692070000	-1.675192000
1	1.035457000	2.600518000	-1.692445000
15	2.027662000	0.418634000	0.081659000
28	0.087533000	-0.577982000	-1.115662000
6	-1.637480000	-1.245728000	-1.768861000
1	-1.419693000	-2.052157000	-2.474830000
1	-2.046406000	-0.392701000	-2.320022000
6	-2.592161000	-1.688480000	-0.707033000
6	-2.355962000	-2.813242000	-0.026522000
9	-3.101947000	-3.328058000	0.948470000
9	-1.269408000	-3.578302000	-0.206211000
9	-0.777219000	0.223514000	0.244217000
6	-3.815548000	-0.897700000	-0.404832000
6	-3.730343000	0.496174000	-0.230040000
6	-5.078153000	-1.508164000	-0.324280000
6	-4.873221000	1.246632000	0.013464000
6	-6.221211000	-0.752939000	-0.075733000
6	-6.136973000	0.637649000	0.092963000
1	-5.165481000	-2.581290000	-0.464294000
1	-4.813407000	2.320623000	0.159095000
1	-7.181920000	-1.256629000	-0.022591000
1	-2.747291000	0.953109000	-0.243371000
8	-7.218267000	2.709474000	0.486232000
6	-7.329957000	1.497724000	0.358953000
6	-8.698503000	0.839996000	0.472951000
1	-8.714660000	0.098940000	1.281108000
1	-9.441409000	1.613335000	0.676338000
1	-8.964228000	0.317177000	-0.453828000
6	1.029576000	-0.836983000	-2.984200000
6	1.127641000	-1.921814000	-2.408897000
6	1.318542000	-3.325275000	-2.021231000
1	0.353014000	-3.830885000	-1.931039000
1	1.919739000	-3.845731000	-2.776779000
1	1.826149000	-3.413076000	-1.055261000
6	1.044140000	0.290105000	-3.926308000
1	1.581207000	1.150291000	-3.513420000
1	1.535467000	-0.006914000	-4.861128000
1	0.024653000	0.614506000	-4.162873000

87

TSB'-C SCF Done: E(RB3LYP) = -1836.9245263

6	3.023120000	0.841325000	-0.820450000
1	2.433255000	1.596348000	-1.348276000
6	4.041181000	0.224695000	-1.797834000
1	4.642740000	-0.538777000	-1.286314000
1	3.531219000	-0.280281000	-2.627683000
6	4.988354000	1.304345000	-2.354084000
1	5.723298000	0.842694000	-3.026438000
1	4.407207000	2.013161000	-2.961550000
6	5.697092000	2.066760000	-1.225121000
1	6.363224000	1.376221000	-0.685934000
1	6.336630000	2.854994000	-1.642672000
6	4.682690000	2.666476000	-0.240386000
1	4.088864000	3.435985000	-0.754059000
1	5.203607000	3.171109000	0.583711000
6	3.729036000	1.598695000	0.324861000
1	2.971403000	2.074999000	0.953398000
1	4.300651000	0.906538000	0.956576000
6	2.379611000	-1.966133000	0.335192000
1	1.540780000	-2.430221000	0.872111000
6	2.809632000	-2.957477000	-0.770249000
1	1.988408000	-3.141847000	-1.469955000
1	3.633979000	-2.532992000	-1.356154000
6	3.258054000	-4.301444000	-0.165802000
1	2.395003000	-4.783617000	0.316679000
1	3.581429000	-4.975638000	-0.969280000
6	4.379666000	-4.122960000	0.865789000
1	4.644799000	-5.089796000	1.311986000
1	5.283704000	-3.753567000	0.359203000
6	3.970256000	-3.124633000	1.958150000
1	4.800254000	-2.959822000	2.657190000
1	3.144326000	-3.550199000	2.547319000
6	3.527872000	-1.780879000	1.352451000
1	3.234323000	-1.084057000	2.146734000
1	4.389488000	-1.326691000	0.847586000
6	0.677913000	-0.751551000	-1.792285000
1	1.364946000	-1.342831000	-2.413782000
6	0.236429000	0.476579000	-2.615914000
1	-0.347781000	1.149816000	-1.982517000
1	1.112375000	1.050786000	-2.937465000
6	-0.568491000	0.055591000	-3.857782000
1	0.086902000	-0.500106000	-4.545825000
1	-0.896946000	0.952698000	-4.398148000
6	-1.774438000	-0.821093000	-3.494811000
1	-2.487642000	-0.231915000	-2.901312000
1	-2.303411000	-1.136902000	-4.403508000

6	-1.335255000	-2.046624000	-2.682008000
1	-0.713495000	-2.699141000	-3.313672000
1	-2.207021000	-2.640503000	-2.379604000
6	-0.537433000	-1.634888000	-1.432931000
1	-0.218514000	-2.533449000	-0.889008000
1	-1.192847000	-1.075883000	-0.753087000
15	1.635207000	-0.297847000	-0.217516000
28	0.298013000	0.905944000	1.190415000
6	-1.304032000	2.199057000	1.911215000
1	-0.767799000	3.136825000	2.027761000
1	-2.013987000	2.061440000	2.724116000
6	-2.066074000	2.139167000	0.620769000
6	-1.808198000	3.117816000	-0.268639000
9	-2.309586000	3.200344000	-1.497929000
9	-1.060203000	4.177297000	-0.034156000
9	0.636081000	2.184992000	-0.098833000
6	-3.092648000	1.096516000	0.398985000
6	-3.139855000	-0.026334000	1.255410000
6	-4.089755000	1.184342000	-0.598150000
6	-4.110601000	-1.008970000	1.111783000
6	-5.060271000	0.198094000	-0.737211000
6	-5.089077000	-0.920125000	0.110940000
1	-4.121105000	2.036065000	-1.263879000
1	-4.129695000	-1.869040000	1.773735000
1	-5.807585000	0.314715000	-1.516592000
1	-2.392915000	-0.133759000	2.033089000
8	-6.085590000	-2.965135000	0.765467000
6	-6.105978000	-2.009062000	0.002077000
6	-7.172295000	-1.911514000	-1.079125000
1	-6.720406000	-1.878627000	-2.077905000
1	-7.824311000	-2.783812000	-1.006856000
1	-7.771337000	-1.000331000	-0.962450000
6	0.113974000	-0.213110000	2.729222000
6	-0.207178000	0.942690000	3.190595000
6	-0.318932000	1.739314000	4.444451000
1	-1.357593000	1.912017000	4.752420000
1	0.184908000	1.191259000	5.249424000
1	0.165533000	2.717899000	4.348237000
6	0.390395000	-1.576806000	3.266106000
1	-0.156483000	-2.344678000	2.705189000
1	1.453483000	-1.828875000	3.202379000
1	0.091170000	-1.656694000	4.319477000

69

Re-L2 SCF Done: E(RB3LYP) = -2111.8923612

6	4.423902000	1.319582000	1.386630000
1	3.794880000	1.088669000	3.422324000
6	3.455962000	1.258326000	2.404654000
6	2.108888000	1.429633000	2.126388000
1	1.399088000	1.407338000	2.946162000
6	1.642657000	1.654774000	0.810886000
6	-0.838636000	1.525362000	1.481233000
1	-0.550174000	1.216442000	2.485185000
1	-1.752155000	2.115163000	1.459870000
6	0.194332000	1.816785000	0.509849000
6	-0.111512000	2.863086000	-0.527596000
9	0.216027000	2.487359000	-1.807686000
9	-1.425923000	3.201069000	-0.581292000
9	0.561994000	4.029879000	-0.323570000
6	2.625384000	1.727888000	-0.202805000
1	4.688164000	1.636324000	-0.740407000
1	2.319993000	1.910580000	-1.226005000
6	3.976504000	1.570000000	0.078063000
6	5.851890000	1.115685000	1.738752000
8	6.195056000	0.825505000	2.880293000
1	7.891098000	1.105981000	1.089422000
6	6.906326000	1.271494000	0.648189000
1	6.747988000	0.550120000	-0.162837000
1	6.870695000	2.272803000	0.202708000
1	0.850977000	-1.474647000	-5.254600000
6	0.553276000	-1.280835000	-4.227181000
6	-0.647655000	-0.671593000	-3.930852000
6	-0.962020000	-0.418965000	-2.587641000
1	-1.886733000	0.075318000	-2.311873000
1	-1.341179000	-0.370741000	-4.709601000
7	-0.169696000	-0.747081000	-1.562431000
6	1.409982000	-1.661856000	-3.173913000
6	0.998884000	-1.400487000	-1.833804000
6	1.858772000	-1.842600000	-0.746236000
6	2.673663000	-2.288878000	-3.435178000
1	2.957214000	-2.459990000	-4.470493000
6	3.502421000	-2.647832000	-2.418788000
1	4.465852000	-3.108593000	-2.622152000
6	3.117047000	-2.436887000	-1.054881000
6	3.949493000	-2.813916000	0.022137000
1	4.919451000	-3.259292000	-0.185592000
6	3.522735000	-2.609831000	1.317157000
1	4.142075000	-2.872484000	2.168791000
1	1.864582000	-1.906991000	2.523797000
6	2.243425000	-2.055616000	1.514346000
7	1.435298000	-1.693063000	0.529575000
28	-0.779946000	0.117846000	0.187126000
7	-2.176411000	-1.194486000	0.854077000
6	-1.653106000	-2.251161000	1.485351000
1	-0.585987000	-2.383290000	1.346595000
6	-2.396446000	-3.128236000	2.289121000
1	-1.898665000	-3.961980000	2.774175000
6	-3.744529000	-2.892275000	2.459205000
1	-4.354921000	-3.528505000	3.095038000
6	-4.341391000	-1.804797000	1.788418000
6	-5.737797000	-1.513219000	1.943689000
1	-6.325023000	-2.150932000	2.599439000
6	-6.310763000	-0.459953000	1.302711000
1	-7.365257000	-0.232645000	1.437448000

6	-5.538463000	0.363131000	0.419755000
6	-4.153482000	0.094225000	0.211977000
6	-3.523365000	-0.985748000	0.956140000
7	-3.414222000	0.802898000	-0.671313000
6	-3.984002000	1.811421000	-1.314752000
1	-3.342781000	2.362368000	-1.998454000
1	-5.737321000	3.026148000	-1.701429000
6	-5.331325000	2.185110000	-1.147906000
6	-6.111696000	1.444688000	-0.284527000
1	-7.163614000	1.677454000	-0.137370000

93

Aa SCF Done: E(RB3LYP) = -1915.6036777

6	-1.811362000	-0.826741000	1.768402000
1	-0.908833000	-0.631289000	2.361752000
6	-2.444346000	-2.129758000	2.294913000
1	-3.372839000	-2.340656000	1.746827000
1	-1.779970000	-2.986040000	2.128391000
6	-2.769943000	-2.011372000	3.795997000
1	-3.245510000	-2.936324000	4.147658000
1	-1.830889000	-1.908474000	4.359262000
6	-3.672065000	-0.802890000	4.087587000
1	-4.657763000	-0.970580000	3.627675000
1	-3.844208000	-0.710374000	5.167569000
6	-3.070040000	0.495110000	3.528734000
1	-2.143975000	0.736849000	4.069315000
1	-3.757457000	1.334745000	3.695216000
6	-2.751112000	0.372477000	2.028509000
1	-2.287155000	1.297042000	1.671404000
1	-3.689406000	0.248749000	1.473707000
6	-2.395484000	-1.548949000	-1.191453000
1	-1.948009000	-1.320437000	-2.169105000
6	-2.676591000	-3.067828000	-1.179563000
1	-1.749878000	-3.641214000	-1.282723000
1	-3.123293000	-3.361295000	-0.221167000
6	-3.629377000	-3.463540000	-2.324801000
1	-3.123265000	-3.289053000	-3.286193000
1	-3.836717000	-4.540331000	-2.274989000
6	-4.939529000	-2.665038000	-2.292547000
1	-5.570009000	-2.933797000	-3.149748000
1	-5.507114000	-2.935844000	-1.389745000
6	-4.665175000	-1.155103000	-2.279263000
1	-5.606363000	-0.596140000	-2.196241000
1	-4.207621000	-0.857039000	-3.234435000
6	-3.725402000	-0.768327000	-1.124783000
1	-3.530952000	0.308090000	-1.141719000
1	-4.230346000	-0.990290000	-0.175698000
6	0.175714000	-2.270508000	0.085064000
1	-0.399413000	-3.194684000	0.228156000
6	1.173626000	-2.165545000	1.256036000
1	1.726305000	-1.222460000	1.191729000
1	0.642774000	-2.153540000	2.214554000
6	2.167848000	-3.341345000	1.247425000
1	1.623643000	-4.278551000	1.439520000
1	2.881913000	-3.221459000	2.072403000
6	2.912085000	-3.453366000	-0.090107000
1	3.547785000	-2.567519000	-0.226651000
1	3.580317000	-4.324215000	-0.082616000
6	1.926524000	-3.551090000	-1.262315000
1	1.371098000	-4.498779000	-1.195026000
1	2.468048000	-3.572604000	-2.216639000
6	0.931938000	-2.376919000	-1.257954000
1	0.226767000	-2.491631000	-2.092366000
1	1.479922000	-1.440882000	-1.431294000
15	-1.079125000	-0.842409000	0.013485000
28	-0.319187000	1.344838000	-0.531275000
6	-1.186252000	1.565450000	-2.459217000
6	-1.728990000	2.403693000	-1.730251000
6	-0.714664000	0.785179000	-3.616489000
1	-0.459328000	-0.243947000	-3.346945000
1	-1.479166000	0.754143000	-4.403316000
1	0.186993000	1.241363000	-4.043605000
6	-2.536097000	3.547868000	-1.235330000
1	-2.459814000	3.585843000	-0.141412000
6	0.699156000	2.959340000	-0.179430000
1	0.072720000	3.713925000	0.292259000
1	1.266742000	3.325495000	-1.033195000
6	1.263512000	1.932263000	0.647189000
6	0.948893000	2.015981000	2.110503000
9	0.966835000	0.804751000	2.741409000
9	1.829795000	2.792415000	2.804903000
9	-0.277797000	2.549973000	2.350146000
6	2.559851000	1.306910000	0.262637000
6	2.891095000	1.155118000	-1.104985000
6	3.520134000	0.873187000	1.200489000
6	4.100008000	0.610679000	-1.507541000
6	4.734429000	0.324903000	0.793907000
6	5.051024000	0.182192000	-0.564620000
1	3.332327000	0.977771000	2.261393000
1	4.338074000	0.501043000	-2.560979000
1	5.442976000	0.014715000	1.556476000
1	2.173158000	1.461907000	-1.859555000
8	6.558284000	-0.511546000	-2.252457000
6	6.336600000	-0.394909000	-1.054072000
6	7.377442000	-0.838965000	-0.035570000
1	7.683387000	-0.004986000	0.607466000
1	6.981315000	-1.625886000	0.617603000
1	8.248575000	-1.220556000	-0.571155000
6	-4.023891000	3.365727000	-1.599796000
1	-4.433936000	2.449417000	-1.164222000
1	-4.607153000	4.213986000	-1.223217000
1	-4.154935000	3.316152000	-2.686589000

6	-1.990213000	4.876909000	-1.798112000
1	-2.069767000	4.893726000	-2.891006000
1	-2.567682000	5.718773000	-1.398516000
1	-0.939789000	5.021186000	-1.530465000

93

Aa' SCF Done: E(RB3LYP) = -1915.6048275

6	-1.566834000	1.939369000	0.936481000
1	-0.544688000	2.340020000	0.911344000
6	-2.190922000	2.320545000	2.293644000
1	-3.231038000	1.968574000	2.333880000
1	-1.662380000	1.836427000	3.123463000
6	-2.173314000	3.848385000	2.493052000
1	-2.645547000	4.104282000	3.450597000
1	-1.129181000	4.187632000	2.558510000
6	-2.873837000	4.581038000	1.339017000
1	-3.947771000	4.341421000	1.360362000
1	-2.794809000	5.667071000	1.476559000
6	-2.293030000	4.170347000	-0.022398000
1	-1.253256000	4.516569000	-0.103376000
1	-2.849196000	4.656583000	-0.834413000
6	-2.320076000	2.643755000	-0.213980000
1	-1.871182000	2.384488000	-1.176732000
1	-3.364350000	2.308766000	-0.244217000
6	-2.813284000	-0.908655000	0.941280000
1	-2.543680000	-1.889287000	0.522741000
6	-3.245608000	-1.146593000	2.404781000
1	-2.421827000	-1.555537000	2.998828000
1	-3.527822000	-0.194811000	2.872928000
6	-4.439686000	-2.117798000	2.478852000
1	-4.118408000	-3.107343000	2.120616000
1	-4.742196000	-2.250281000	3.525695000
6	-5.628899000	-1.637255000	1.635742000
1	-6.441535000	-2.374127000	1.668440000
1	-6.030457000	-0.709740000	2.070624000
6	-5.204511000	-1.369987000	0.185427000
1	-6.046853000	-0.966722000	-0.391635000
1	-4.923308000	-2.319047000	-0.294799000
6	-4.015047000	-0.396775000	0.118238000
1	-3.721840000	-0.237954000	-0.924394000
1	-4.337953000	0.575231000	0.512695000
6	-0.130064000	-0.388362000	2.087040000
1	-0.759393000	-0.303621000	2.982761000
6	1.091758000	0.529201000	2.296933000
1	1.724605000	0.518630000	1.402592000
1	0.774239000	1.567492000	2.445486000
6	1.918215000	0.079219000	3.516042000
1	1.316610000	0.210846000	4.428358000
1	2.794240000	0.731564000	3.623143000
6	2.357665000	-1.387427000	3.405681000
1	3.061163000	-1.492760000	2.568444000
1	2.898551000	-1.691739000	4.310843000
6	1.151705000	-2.308484000	3.173266000
1	0.510385000	-2.304678000	4.067661000
1	1.483922000	-3.345018000	3.031748000
6	0.324260000	-1.858113000	1.957175000
1	-0.541776000	-2.522354000	1.838831000
1	0.930231000	-1.961969000	1.046738000
15	-1.219091000	0.101095000	0.603433000
28	-0.329147000	-0.286844000	-1.562033000
6	-1.414711000	-2.087591000	-2.093861000
6	-1.872949000	-1.151087000	-2.753914000
6	-1.112005000	-3.436067000	-1.558568000
1	-0.852505000	-3.340674000	-0.496024000
6	-2.603421000	-0.276594000	-3.683453000
1	-3.417468000	-0.829116000	-4.169829000
1	-3.038244000	0.586064000	-3.166647000
1	-1.937985000	0.108621000	-4.463806000
6	0.880798000	-0.012911000	-3.044899000
1	0.375315000	0.459730000	-3.884800000
1	1.447380000	-0.900115000	-3.321928000
6	1.358875000	0.818046000	-1.975016000
6	1.069813000	2.283626000	-2.113132000
9	0.953142000	2.933796000	-0.917302000
9	2.037757000	2.959917000	-2.793835000
9	-0.088029000	2.523156000	-2.786770000
6	2.580428000	0.439663000	-1.213364000
6	2.929144000	-0.923186000	-1.061088000
6	3.455803000	1.384351000	-0.636503000
6	4.074205000	-1.315184000	-0.387141000
6	4.606817000	0.988962000	0.040567000
6	4.941791000	-0.365180000	0.180371000
1	3.251845000	2.443318000	-0.725759000
1	4.325268000	-2.366059000	-0.281473000
1	5.250684000	1.757993000	0.457547000
1	2.275973000	-1.685844000	-1.472127000
8	6.389367000	-2.038286000	1.021388000
6	6.159819000	-0.842003000	0.896215000
6	7.122548000	0.187263000	1.472471000
1	7.505877000	0.852372000	0.689416000
1	6.626553000	0.817277000	2.220809000
1	7.956432000	-0.338691000	1.940818000
6	0.097955000	-4.063505000	-2.280745000
1	-0.114527000	-4.195467000	-3.347460000
1	0.987991000	-3.434769000	-2.187760000
1	0.324350000	-5.045554000	-1.850142000
6	-2.351566000	-4.348428000	-1.669546000
1	-3.206659000	-3.927108000	-1.131347000
1	-2.641224000	-4.480108000	-2.718024000
1	-2.131852000	-5.336322000	-1.248280000

93

TSAa-Ba SCF Done: E(RB3LYP) = -1915.5855701

6	-0.598463000	2.237289000	-0.312259000

1	0.423625000	2.040054000	-0.654546000
6	-0.527454000	3.428999000	0.671790000
1	-1.527057000	3.671100000	1.052788000
1	0.090580000	3.190456000	1.542168000
6	0.057159000	4.676216000	-0.019519000
1	0.087215000	5.508802000	0.695406000
1	1.099545000	4.471942000	-0.305238000
6	-0.742594000	5.069716000	-1.267688000
1	-1.752404000	5.386068000	-0.965435000
1	-0.279352000	5.934120000	-1.760408000
6	-0.849669000	3.890352000	-2.243415000
1	0.142190000	3.655942000	-2.649598000
1	-1.482653000	4.158728000	-3.099266000
6	-1.424616000	2.639402000	-1.555210000
1	-1.474979000	1.812525000	-2.269823000
1	-2.453944000	2.863761000	-1.247775000
6	-2.995799000	0.560086000	0.670299000
1	-3.138403000	-0.335141000	1.289646000
6	-3.559814000	1.761517000	1.457864000
1	-3.008677000	1.912448000	2.393476000
1	-3.439198000	2.679199000	0.868566000
6	-5.056646000	1.572090000	1.765311000
1	-5.182203000	0.712557000	2.440368000
1	-5.436667000	2.449753000	2.303915000
6	-5.868042000	1.336362000	0.484246000
1	-6.924376000	1.166153000	0.728070000
1	-5.832545000	2.242563000	-0.138691000
6	-5.306832000	0.149547000	-0.312383000
1	-5.864145000	0.018597000	-1.248805000
1	-5.454290000	-0.771208000	0.270413000
6	-3.808925000	0.325735000	-0.622440000
1	-3.420574000	-0.552039000	-1.150182000
1	-3.691866000	1.175054000	-1.303713000
6	-0.411499000	0.609952000	2.160563000
1	-0.780544000	1.550735000	2.589762000
6	1.130580000	0.652613000	2.141460000
1	1.503879000	-0.275086000	1.695015000
1	1.499858000	1.463902000	1.504314000
6	1.711349000	0.799921000	3.557980000
1	1.415936000	1.774024000	3.976552000
1	2.807021000	0.799760000	3.504613000
6	1.221720000	-0.322093000	4.483305000
1	1.630798000	-1.280989000	4.133181000
1	1.602069000	-0.172927000	5.501782000
6	-0.310917000	-0.398934000	4.492645000
1	-0.717559000	0.510461000	4.960007000
1	-0.648799000	-1.242635000	5.108282000
6	-0.888732000	-0.535895000	3.072123000
1	-1.982078000	-0.557926000	3.133618000
1	-0.577980000	-1.494459000	2.638207000
15	-1.113554000	0.529488000	0.393214000
28	-0.402679000	-1.301949000	-0.729203000
6	-1.445532000	-2.865123000	-0.266403000
6	-0.559221000	-3.330461000	-1.089549000
6	0.853770000	-2.245341000	-2.112104000
1	0.393917000	-2.450610000	-3.074721000
1	1.612920000	-2.976172000	-1.853868000
6	1.224463000	-0.854597000	-1.876704000
6	0.932386000	0.009386000	-3.067752000
9	0.978862000	1.348398000	-2.817687000
9	1.812405000	-0.188256000	-4.090237000
9	-0.301330000	-0.232886000	-3.586382000
6	2.482692000	-0.600495000	-1.118929000
6	2.911079000	-1.527017000	-0.136615000
6	3.310797000	0.522232000	-1.331290000
6	4.083470000	-1.344388000	0.579432000
6	4.486800000	0.704468000	-0.607804000
6	4.898186000	-0.220177000	0.362129000
1	3.048523000	1.259313000	-2.078236000
1	4.393579000	-2.065071000	1.329679000
1	5.090210000	1.583409000	-0.815658000
1	2.296601000	-2.394796000	0.083794000
8	6.449648000	-0.896936000	2.016439000
6	6.143354000	-0.068433000	1.168617000
6	7.035059000	1.140957000	0.922232000
1	6.492931000	2.076804000	1.104491000
1	7.892792000	1.084544000	1.594865000
1	7.389051000	1.166491000	-0.115425000
6	-0.209856000	-4.661194000	-1.668578000
1	-0.275403000	-4.669613000	-2.764018000
1	0.806367000	-4.979912000	-1.403030000
1	-0.907857000	-5.414922000	-1.284816000
6	-2.565236000	-3.644426000	0.396037000
1	-2.259612000	-4.703711000	0.367404000
6	-3.865697000	-3.547903000	-0.424473000
6	-2.817335000	-3.313077000	1.872006000
1	-1.904615000	-3.419376000	2.466588000
1	-3.186252000	-2.290906000	1.999956000
1	-3.571790000	-3.993888000	2.285114000
1	-4.254687000	-2.526040000	-0.435590000
1	-4.636835000	-4.198505000	0.006724000
1	-3.700486000	-3.854602000	-1.462758000

93

TSAa'-Ba’ SCF Done: E(RB3LYP) = -1915.5908913

6	1.335546000	-1.987659000	-0.890738000
1	0.286401000	-1.958776000	-1.210193000
6	1.626335000	-3.412152000	-0.366530000
1	2.668459000	-3.487788000	-0.032904000
1	1.001919000	-3.651650000	0.499857000
6	1.374759000	-4.459978000	-1.467877000
1	1.603054000	-5.461487000	-1.080585000
1	0.305074000	-4.459865000	-1.723829000
6	2.198037000	-4.173315000	-2.730933000

1	3.267297000	-4.294031000	-2.500227000
1	1.963121000	-4.907458000	-3.512181000
6	1.947137000	-2.748116000	-3.242839000
1	0.911591000	-2.657575000	-3.595572000
1	2.591680000	-2.533500000	-4.105039000
6	2.194535000	-1.701006000	-2.143108000
1	1.989008000	-0.698822000	-2.530932000
1	3.257313000	-1.730088000	-1.869462000
6	3.210571000	-0.178060000	0.784025000
1	3.102271000	0.430607000	1.692123000
6	4.115048000	-1.375106000	1.144225000
1	3.646559000	-2.005687000	1.909555000
1	4.261846000	-2.008121000	0.260179000
6	5.491861000	-0.895720000	1.640887000
1	5.363029000	-0.344963000	2.584528000
1	6.125577000	-1.762357000	1.869043000
6	6.177832000	0.014079000	0.611287000
1	7.136202000	0.377326000	1.003582000
1	6.410784000	-0.571815000	-0.290270000
6	5.275584000	1.195992000	0.227258000
1	5.756270000	1.807595000	-0.546914000
1	5.145738000	1.848324000	1.103969000
6	3.896075000	0.724581000	-0.266329000
1	3.256543000	1.584275000	-0.494220000
1	4.026709000	0.174971000	-1.205708000
6	0.727280000	-1.340368000	1.962884000
1	1.335956000	-2.235557000	2.151769000
6	-0.744949000	-1.771397000	1.784453000
1	-1.349021000	-0.887291000	1.547331000
1	-0.855533000	-2.453021000	0.933579000
6	-1.302497000	-2.435960000	3.054491000
1	-0.767202000	-3.379222000	3.241766000
1	-2.355786000	-2.698091000	2.895930000
6	-1.159465000	-1.518659000	4.276466000
1	-1.796186000	-0.632805000	4.137876000
1	-1.519840000	-2.025394000	5.180567000
6	0.297227000	-1.072404000	4.460274000
1	0.915502000	-1.946254000	4.714934000
1	0.381667000	-0.374771000	5.303551000
6	0.856663000	-0.410537000	3.188076000
1	1.903472000	-0.132389000	3.359182000
1	0.308658000	0.520260000	2.989339000
15	1.394430000	-0.554159000	0.367196000
28	0.275223000	1.281606000	-0.165059000
6	0.991254000	2.669101000	0.889076000
6	0.086792000	3.335646000	0.238914000
6	1.980669000	3.047549000	1.940049000
1	1.898930000	4.109369000	2.216753000
1	1.843092000	2.454192000	2.851345000
1	3.008445000	2.874680000	1.600245000
6	-1.049523000	2.514827000	-1.217407000
1	-0.588578000	3.075169000	-2.023773000
1	-1.942743000	2.998567000	-0.839045000
6	-1.211941000	1.075042000	-1.496627000
6	-0.756893000	0.699468000	-2.871238000
9	-0.558033000	-0.640815000	-3.040950000
9	-1.640810000	1.049713000	-3.850183000
9	0.419759000	1.298898000	-3.205379000
6	-2.420476000	0.400122000	-0.953744000
6	-2.997725000	0.855942000	0.258043000
6	-3.056055000	-0.693709000	-1.581666000
6	-4.124811000	0.260160000	0.801663000
6	-4.187002000	-1.290111000	-1.031898000
6	-4.745669000	-0.830948000	0.170072000
1	-2.675046000	-1.081142000	-2.517332000
1	-4.550109000	0.623224000	1.732196000
1	-4.638188000	-2.124280000	-1.561864000
1	-2.537895000	1.681417000	0.792521000
8	-6.389320000	-1.008473000	1.863788000
6	-5.950642000	-1.436272000	0.803196000
6	-6.636447000	-2.604747000	0.107958000
1	-5.951192000	-3.452628000	-0.011877000
1	-7.492330000	-2.915373000	0.709954000
1	-6.981614000	-2.321116000	-0.893618000
6	-0.387090000	4.773620000	0.296767000
1	0.354467000	5.251253000	0.955639000
6	-1.753704000	4.942073000	0.991353000
6	-0.338243000	5.540587000	-1.038862000
1	0.620941000	5.393868000	-1.547400000
1	-1.138328000	5.238007000	-1.721884000
1	-0.461120000	6.613545000	-0.850560000
1	-2.577923000	4.535950000	0.394560000
1	-1.963050000	6.006820000	1.149578000
1	-1.762711000	4.443543000	1.966222000

93

Ba SCF Done: E(RB3LYP) = -1915.6187235

6	1.141268000	-1.935031000	-1.241186000
1	0.058962000	-1.846799000	-1.420960000
6	1.445332000	-3.432827000	-1.022640000
1	2.519356000	-3.576025000	-0.849746000
1	0.930054000	-3.816213000	-0.135609000
6	1.018757000	-4.257542000	-2.252351000
1	1.264125000	-5.314911000	-2.089585000
1	-0.074895000	-4.203474000	-2.357175000
6	1.678009000	-3.747560000	-3.541938000
1	2.763063000	-3.921316000	-3.485726000
1	1.315289000	-4.319943000	-4.405004000
6	1.417952000	-2.248260000	-3.746763000
1	0.347600000	-2.079726000	-3.932648000
1	1.949417000	-1.885904000	-4.635699000
6	1.847547000	-1.428764000	-2.517945000
1	1.633166000	-0.368279000	-2.683480000
1	2.934309000	-1.524181000	-2.393591000

6	3.219877000	-0.484788000	0.582449000
1	3.187665000	-0.003883000	1.571793000
6	4.103345000	-1.741326000	0.712246000
1	3.660968000	-2.462154000	1.410573000
1	4.170822000	-2.243734000	-0.261769000
6	5.523111000	-1.368117000	1.178283000
1	5.471648000	-0.961333000	2.199059000
1	6.143935000	-2.271508000	1.233990000
6	6.170155000	-0.330682000	0.248613000
1	7.159774000	-0.046798000	0.628084000
1	6.333117000	-0.784915000	-0.740170000
6	5.281661000	0.912591000	0.091626000
1	5.733097000	1.616791000	-0.618499000
1	5.217190000	1.439124000	1.055269000
6	3.864097000	0.540880000	-0.376876000
1	3.237674000	1.437165000	-0.447469000
1	3.922685000	0.121958000	-1.388841000
6	0.749440000	-1.746242000	1.718709000
1	1.300541000	-2.696511000	1.740720000
6	-0.756642000	-2.050763000	1.551306000
1	-1.304942000	-1.104155000	1.453343000
1	-0.940360000	-2.610119000	0.626466000
6	-1.320050000	-2.838550000	2.746482000
1	-0.856667000	-3.836089000	2.778840000
1	-2.396286000	-2.994976000	2.603889000
6	-1.059039000	-2.112787000	4.072870000
1	-1.625305000	-1.170405000	4.085602000
1	-1.428061000	-2.711498000	4.914920000
6	0.434928000	-1.809637000	4.248397000
1	0.989366000	-2.754596000	4.348994000
1	0.606701000	-1.247174000	5.174955000
6	1.000078000	-1.016843000	3.056170000
1	2.072101000	-0.847705000	3.212903000
1	0.525423000	-0.026709000	3.022334000
15	1.374559000	-0.746401000	0.233513000
28	0.007415000	1.162138000	-0.278975000
6	0.174329000	2.804001000	0.631365000
6	-0.700260000	3.785524000	0.320762000
6	1.298504000	2.843544000	1.657220000
1	1.777565000	1.854153000	1.634788000
6	-0.790262000	5.177554000	0.905900000
1	-0.034700000	5.385348000	1.664476000
1	-0.684237000	5.932802000	0.113558000
1	-1.776852000	5.344987000	1.362428000
6	-1.696273000	3.461526000	-0.757697000
1	-1.563631000	4.146590000	-1.607267000
1	-2.728811000	3.631196000	-0.416823000
6	-1.493726000	1.996562000	-1.203179000
6	-1.118114000	1.915285000	-2.658873000
9	-0.581325000	0.686653000	-2.987263000
9	-2.154860000	2.097260000	-3.520985000
9	-0.177223000	2.824039000	-3.003154000
6	-2.536372000	1.020896000	-0.755082000
6	-3.142379000	1.187864000	0.518240000
6	-2.937579000	-0.111563000	-1.501016000
6	-4.075718000	0.290321000	1.006027000
6	-3.882672000	-1.009523000	-1.008049000
6	-4.467368000	-0.833201000	0.252607000
1	-2.530159000	-0.287448000	-2.487842000
1	-4.524901000	0.433530000	1.983844000
1	-4.165652000	-1.854284000	-1.629814000
1	-2.843925000	2.028447000	1.136090000
6	-5.471375000	-1.771728000	0.830202000
8	-5.911091000	-1.606187000	1.961054000
6	-5.947998000	-2.947685000	-0.011710000
1	-6.403872000	-2.604087000	-0.948047000
1	-6.684289000	-3.512204000	0.563197000
1	-5.114641000	-3.608811000	-0.278836000
6	0.795903000	3.028718000	3.105262000
1	0.027636000	2.287422000	3.351056000
1	1.622314000	2.913602000	3.818725000
1	0.357624000	4.019280000	3.261688000
6	2.407302000	3.857285000	1.302248000
1	2.050734000	4.890018000	1.370056000
1	3.259932000	3.753528000	1.986179000
1	2.767100000	3.701195000	0.279508000

93

Ba’ SCF Done: E(RB3LYP) = -1915.618188

6	-1.924145000	-1.707982000	1.233488000
1	-0.890742000	-1.964904000	1.513209000
6	-2.688798000	-3.033860000	1.041908000
1	-3.736605000	-2.822711000	0.793061000
1	-2.276739000	-3.611486000	0.206748000
6	-2.639210000	-3.880188000	2.327910000
1	-3.208269000	-4.807366000	2.182442000
1	-1.598138000	-4.179223000	2.519672000
6	-3.177586000	-3.105856000	3.540179000
1	-4.253596000	-2.922594000	3.401147000
1	-3.079756000	-3.710948000	4.450238000
6	-2.455619000	-1.761192000	3.712660000
1	-1.403815000	-1.937482000	3.979624000
1	-2.897093000	-1.195198000	4.542482000
6	-2.510574000	-0.919584000	2.425863000
1	-1.969793000	0.021140000	2.570816000
1	-3.556898000	-0.661154000	2.218812000
6	-3.253022000	-0.004802000	-1.041824000
1	-2.887911000	0.530060000	-1.930746000
6	-4.274191000	-1.052695000	-1.529505000
1	-3.797083000	-1.801224000	-2.172507000
1	-4.691411000	-1.592965000	-0.670232000
6	-5.422819000	-0.371598000	-2.297498000
1	-5.020997000	0.080308000	-3.216412000
1	-6.154191000	-1.125813000	-2.614725000

6	-6.107073000	0.714896000	-1.454267000
1	-6.882356000	1.219758000	-2.044131000
1	-6.620315000	0.242019000	-0.603735000
6	-5.090648000	1.737286000	-0.922854000
1	-5.588904000	2.467432000	-0.272852000
1	-4.667526000	2.304619000	-1.764908000
6	-3.950072000	1.049014000	-0.153416000
1	-3.225396000	1.788423000	0.206866000
1	-4.369421000	0.562468000	0.735915000
6	-0.949980000	-1.848895000	-1.586934000
1	-1.717400000	-2.615765000	-1.762080000
6	0.326175000	-2.546557000	-1.064994000
1	1.077015000	-1.784479000	-0.814885000
1	0.117337000	-3.097379000	-0.140510000
6	0.913522000	-3.514004000	-2.107826000
1	0.209356000	-4.343892000	-2.270050000
1	1.835335000	-3.957979000	-1.712001000
6	1.190919000	-2.811766000	-3.443275000
1	1.986956000	-2.067309000	-3.300965000
1	1.563484000	-3.532055000	-4.182171000
6	-0.071509000	-2.114308000	-3.967642000
1	-0.829434000	-2.870782000	-4.220548000
1	0.147913000	-1.569712000	-4.894692000
6	-0.654051000	-1.141738000	-2.927313000
1	-1.561879000	-0.677582000	-3.331565000
1	0.065662000	-0.331027000	-2.748072000
15	-1.620375000	-0.624880000	-0.301754000
28	0.041005000	0.927063000	0.366280000
6	0.125503000	2.540878000	-0.609358000
6	1.048302000	3.450882000	-0.228737000
6	-0.862447000	2.688536000	-1.738891000
1	-1.895463000	2.733714000	-1.371399000
1	-0.689977000	3.597107000	-2.326489000
1	-0.807196000	1.844805000	-2.438178000
6	1.922769000	3.048346000	0.930339000
1	1.737815000	3.716031000	1.783597000
1	2.989519000	3.178967000	0.690461000
6	1.616925000	1.586511000	1.313679000
6	1.200454000	1.447069000	2.751042000
9	0.540085000	0.246385000	2.964684000
9	2.213041000	1.463883000	3.657550000
9	0.330491000	2.407329000	3.140018000
6	2.598202000	0.562386000	0.834769000
6	3.235397000	0.743000000	-0.420904000
6	2.920340000	-0.620067000	1.540427000
6	4.125844000	-0.186697000	-0.928496000
6	3.820120000	-1.551991000	1.026824000
6	4.439325000	-1.359728000	-0.215363000
1	2.485612000	-0.811638000	2.512692000
1	4.602943000	-0.029567000	-1.890896000
1	4.041175000	-2.435693000	1.618905000
1	2.995927000	1.622596000	-1.008870000
6	5.405455000	-2.326090000	-0.810050000
8	5.892802000	-2.131120000	-1.916472000
6	5.784050000	-3.569448000	-0.016118000
1	6.231872000	-3.301301000	0.948338000
1	6.501585000	-4.149983000	-0.598756000
1	4.904130000	-4.189271000	0.194950000
6	1.305208000	4.849444000	-0.806739000
1	2.095637000	5.284652000	-0.177747000
6	0.097974000	5.799763000	-0.680918000
6	1.872952000	4.826365000	-2.240356000
1	2.769359000	4.198092000	-2.298288000
1	1.149756000	4.436755000	-2.964569000
1	2.151200000	5.838329000	-2.561271000
1	-0.741058000	5.489708000	-1.312096000
1	0.378622000	6.817860000	-0.979783000
1	-0.262471000	5.837685000	0.353267000

93

TSBa-Ca SCF Done: E(RB3LYP) = -1915.5963442

6	2.656110000	-1.551131000	-0.610547000
1	1.880578000	-2.321559000	-0.681912000
6	3.911450000	-2.182556000	0.022832000
1	4.713831000	-1.435751000	0.086479000
1	3.710608000	-2.518223000	1.047844000
6	4.409009000	-3.371379000	-0.821347000
1	5.318310000	-3.790608000	-0.371272000
1	3.651640000	-4.168590000	-0.799928000
6	4.671592000	-2.959837000	-2.277235000
1	5.510696000	-2.248444000	-2.305139000
1	4.982021000	-3.831458000	-2.867399000
6	3.431631000	-2.304509000	-2.902300000
1	2.624479000	-3.046957000	-2.979702000
1	3.650974000	-1.972006000	-3.925062000
6	2.932600000	-1.112377000	-2.066282000
1	2.018851000	-0.708621000	-2.510960000
1	3.687880000	-0.317471000	-2.095081000
6	2.989357000	1.062626000	1.035355000
1	2.337717000	1.861921000	1.409736000
6	3.908849000	0.642406000	2.203930000
1	3.321046000	0.268407000	3.048685000
1	4.567179000	-0.177351000	1.891316000
6	4.771690000	1.825449000	2.681575000
1	4.117180000	2.597898000	3.111733000
1	5.433100000	1.491640000	3.491408000
6	5.591366000	2.435660000	1.536690000
1	6.163373000	3.299935000	1.896898000
1	6.327474000	1.697589000	1.184413000
6	4.684621000	2.844813000	0.368187000
1	5.285167000	3.227640000	-0.466804000
1	4.029641000	3.667936000	0.686188000
6	3.823552000	1.665212000	-0.116956000
1	3.168444000	1.985910000	-0.935660000

1	4.488461000	0.895071000	-0.527480000
6	1.220929000	-1.163835000	1.990031000
1	2.137998000	-1.442065000	2.526506000
6	0.437038000	-2.458705000	1.685641000
1	-0.428213000	-2.223056000	1.055206000
1	1.056302000	-3.154005000	1.108879000
6	-0.029888000	-3.153672000	2.976874000
1	0.848355000	-3.502415000	3.540799000
1	-0.608759000	-4.049496000	2.717986000
6	-0.859009000	-2.216706000	3.864921000
1	-1.791193000	-1.952828000	3.344029000
1	-1.148986000	-2.726229000	4.792538000
6	-0.077768000	-0.934809000	4.180764000
1	0.798380000	-1.184255000	4.797840000
1	-0.691990000	-0.245946000	4.774338000
6	0.386763000	-0.229221000	2.895226000
1	0.958114000	0.670288000	3.157308000
1	-0.493378000	0.112745000	2.332955000
15	1.743773000	-0.240442000	0.414722000
28	-0.161730000	0.239035000	-0.815865000
6	-0.480882000	2.125179000	-0.635630000
6	-1.128788000	2.595822000	-1.726468000
6	-0.138854000	2.947010000	0.605999000
1	0.334411000	2.261821000	1.317258000
6	-1.514225000	4.022539000	-2.058165000
1	-1.213915000	4.746717000	-1.302178000
1	-1.061845000	4.333079000	-3.012125000
1	-2.603020000	4.113270000	-2.191306000
6	-1.569853000	1.581697000	-2.769336000
1	-0.824611000	1.485279000	-3.567779000
1	-2.490117000	1.919718000	-3.272544000
6	-1.813427000	0.216197000	-2.112996000
6	-1.283876000	-0.915069000	-2.747340000
9	-0.138529000	-1.629445000	-1.394048000
9	-1.915019000	-2.062001000	-2.873813000
9	-0.378522000	-0.799731000	-3.699619000
6	-3.006250000	0.020831000	-1.237545000
6	-3.971822000	1.037864000	-1.091488000
6	-3.205479000	-1.169976000	-0.504871000
6	-5.084632000	0.865206000	-0.279244000
6	-4.322494000	-1.340389000	0.304385000
6	-5.286682000	-0.327596000	0.431704000
1	-2.466757000	-1.960185000	-0.562686000
1	-5.825457000	1.652351000	-0.180479000
1	-4.435160000	-2.275514000	0.845316000
1	-3.850624000	1.978725000	-1.615164000
6	-6.505622000	-0.458037000	1.282715000
8	-7.314959000	0.455929000	1.369686000
6	-6.731583000	-1.756166000	2.046032000
1	-6.790761000	-2.611981000	1.362776000
1	-7.665956000	-1.674353000	2.604210000
1	-5.908943000	-1.953064000	2.744234000
6	-1.403727000	3.460525000	1.328285000
1	-2.097453000	2.637584000	1.531485000
1	-1.138792000	3.928831000	2.285565000
1	-1.942927000	4.204329000	0.732434000
6	0.875270000	4.088592000	0.375449000
1	0.444632000	4.920284000	-0.189732000
1	1.221212000	4.493321000	1.336097000
1	1.749162000	3.737008000	-0.181945000

93

TSBa'-Ca' SCF Done: E(RB3LYP) = -1915.5980231

6	-2.714765000	-1.117288000	1.199680000
1	-1.943447000	-1.729963000	1.679954000
6	-3.951350000	-2.000837000	0.945681000
1	-4.753100000	-1.400272000	0.496067000
1	-3.722373000	-2.805617000	0.236466000
6	-4.467600000	-2.603231000	2.265760000
1	-5.360883000	-3.211058000	2.071776000
1	-3.705545000	-3.284991000	2.670968000
6	-4.776411000	-1.512055000	3.301052000
1	-5.623213000	-0.905990000	2.945029000
1	-5.095784000	-1.967147000	4.247240000
6	-3.561991000	-0.600341000	3.530587000
1	-2.753696000	-1.179003000	4.000232000
1	-3.817711000	0.205935000	4.229963000
6	-3.039017000	0.001724000	2.214158000
1	-2.141958000	0.594938000	2.414663000
1	-3.796275000	0.683283000	1.806621000
6	-2.925303000	0.348493000	-1.547452000
1	-2.225464000	0.713133000	-2.308829000
6	-3.963650000	-0.524920000	-2.285866000
1	-3.481120000	-1.381974000	-2.768500000
1	-4.693723000	-0.931936000	-1.576445000
6	-4.705684000	0.303473000	-3.352151000
1	-3.990535000	0.612018000	-4.128907000
1	-5.454877000	-0.325319000	-3.849988000
6	-5.368545000	1.550180000	-2.749664000
1	-5.854409000	2.140091000	-3.536962000
1	-6.163970000	1.236822000	-2.057110000
6	-4.348263000	2.410030000	-1.989740000
1	-4.846571000	3.264618000	-1.514750000
1	-3.620538000	2.828499000	-2.700516000
6	-3.603742000	1.587364000	-0.924188000
1	-2.863083000	2.210700000	-0.409255000
1	-4.327257000	1.263247000	-0.164878000
6	-1.249838000	-2.103251000	-1.217050000
1	-2.170037000	-2.588498000	-1.570868000
6	-0.518262000	-3.098934000	-0.289474000
1	0.351904000	-2.609152000	0.163301000
1	-1.168061000	-3.399948000	0.539480000
6	-0.064040000	-4.353007000	-1.056856000
1	-0.948549000	-4.907451000	-1.404866000

1	0.474010000	-5.022541000	-0.373682000
6	0.814758000	-3.997219000	-2.263105000
1	1.747990000	-3.534756000	-1.909299000
1	1.099764000	-4.905020000	-2.809795000
6	0.088052000	-3.018915000	-3.195242000
1	-0.786698000	-3.519166000	-3.637114000
1	0.737214000	-2.728148000	-4.030777000
6	-0.372306000	-1.759580000	-2.441533000
1	-0.912295000	-1.099135000	-3.131401000
1	0.508502000	-1.200762000	-2.095871000
15	-1.759142000	-0.509340000	-0.320017000
28	0.107961000	0.512557000	0.545931000
6	0.393314000	2.059888000	-0.539991000
6	1.049082000	3.023882000	0.147131000
6	-0.057436000	2.219330000	-1.973276000
1	-1.105711000	2.535417000	-2.053618000
1	0.538478000	2.958233000	-2.519873000
1	0.038721000	1.275668000	-2.522959000
6	1.595489000	2.652618000	1.515851000
1	0.934901000	3.016819000	2.312761000
1	2.558764000	3.155695000	1.696096000
6	1.781370000	1.135241000	1.648220000
6	1.240717000	0.511901000	2.781701000
9	0.071394000	-0.781418000	2.012467000
9	1.857907000	-0.410443000	3.489054000
9	0.352115000	1.127856000	3.537344000
6	2.941760000	0.464760000	0.989285000
6	3.942762000	1.213203000	0.337974000
6	3.071600000	-0.941797000	0.968795000
6	5.019930000	0.594070000	-0.281939000
6	4.153293000	-1.558093000	0.351377000
6	5.151164000	-0.802826000	-0.285770000
1	2.303955000	-1.550988000	1.430307000
1	5.787528000	1.182136000	-0.775078000
1	4.210893000	-2.642662000	0.365592000
1	3.878688000	2.293996000	0.311449000
6	6.333855000	-1.410973000	-0.962515000
8	7.173107000	-0.712303000	-1.515327000
6	6.484784000	-2.926179000	-0.953398000
1	6.536209000	-3.312118000	0.071835000
1	7.401403000	-3.188668000	-1.484662000
1	5.631391000	-3.412042000	-1.441925000
6	1.301483000	4.484094000	-0.261937000
1	1.633524000	4.991982000	0.656611000
6	0.037807000	5.238698000	-0.715901000
6	2.453475000	4.651589000	-1.275475000
1	3.376569000	4.186177000	-0.913033000
1	2.216462000	4.197579000	-2.243286000
1	2.661763000	5.715150000	-1.449049000
1	-0.343778000	4.874883000	-1.674482000
1	0.254636000	6.308085000	-0.833330000
1	-0.764937000	5.135648000	0.023342000

93

Ca SCF Done: E(RB3LYP) = -1915.6066923

6	2.623295000	1.451931000	0.901091000
1	1.882195000	2.258040000	0.874782000
6	4.005240000	2.021539000	0.525571000
1	4.765389000	1.230857000	0.584878000
1	4.016752000	2.395163000	-0.505255000
6	4.396810000	3.157322000	1.490357000
1	5.391261000	3.540198000	1.226721000
1	3.693834000	3.993109000	1.361885000
6	4.372865000	2.691692000	2.953515000
1	5.166284000	1.944668000	3.106832000
1	4.602464000	3.531382000	3.621752000
6	3.016492000	2.071356000	3.320564000
1	2.237958000	2.846888000	3.288972000
1	3.038204000	1.690129000	4.349515000
6	2.629199000	0.936407000	2.355416000
1	1.643810000	0.547060000	2.622229000
1	3.344930000	0.113349000	2.472086000
6	2.995380000	-1.158090000	-0.773487000
1	2.355210000	-1.833873000	-1.353300000
6	4.211052000	-0.812054000	-1.662800000
1	3.888263000	-0.327678000	-2.590117000
1	4.868017000	-0.101636000	-1.146585000
6	5.017746000	-2.075581000	-2.015910000
1	4.397865000	-2.735262000	-2.640770000
1	5.887155000	-1.795424000	-2.624363000
6	5.462370000	-2.838669000	-0.761275000
1	6.003946000	-3.750643000	-1.042259000
1	6.168626000	-2.217876000	-0.189882000
6	4.257878000	-3.184452000	0.124721000
1	4.588772000	-3.683403000	1.044458000
1	3.613054000	-3.899563000	-0.404471000
6	3.444187000	-1.929225000	0.487365000
1	2.571799000	-2.202414000	1.092583000
1	4.073880000	-1.279172000	1.107343000
6	1.735273000	1.303405000	-1.946882000
1	2.776724000	1.442611000	-2.268651000
6	1.107601000	2.700180000	-1.747547000
1	0.113752000	2.589141000	-1.304582000
1	1.693893000	3.278950000	-1.025321000
6	1.040407000	3.479282000	-3.072756000
1	2.061202000	3.698611000	-3.421511000
1	0.557343000	4.448930000	-2.897147000
6	0.295256000	2.699839000	-4.163873000
1	-0.756982000	2.576081000	-3.868287000
1	0.293860000	3.263627000	-5.105411000
6	0.927626000	1.317675000	-4.370080000
1	1.945512000	1.438612000	-4.770198000
1	0.365079000	0.742441000	-5.116452000
6	0.989129000	0.525712000	-3.052947000

1	1.464256000	-0.446554000	-3.234487000
1	-0.032922000	0.316225000	-2.708740000
15	1.819834000	0.276659000	-0.349067000
28	-0.235073000	-0.141600000	0.446678000
6	-0.560693000	-2.011206000	0.116080000
6	-1.262211000	-2.633255000	1.090910000
6	-0.288878000	-2.613418000	-1.267689000
1	0.336908000	-1.891957000	-1.802299000
6	-1.837437000	-4.037376000	1.076834000
1	-1.649594000	-4.582651000	0.154172000
1	-1.425814000	-4.633916000	1.905418000
1	-2.926464000	-4.009722000	1.227643000
6	-1.591126000	-1.887261000	2.383537000
1	-0.740775000	-1.906218000	3.074212000
1	-2.404516000	-2.404310000	2.912329000
6	-2.012890000	-0.443606000	2.139497000
6	-1.443812000	0.531481000	2.876080000
9	-0.275530000	1.685690000	0.709306000
9	-1.808497000	1.791434000	2.910602000
9	-0.443639000	0.329214000	3.727118000
6	-3.213336000	-0.137176000	1.304388000
6	-4.294294000	-1.038221000	1.268536000
6	-3.305035000	1.044604000	0.544441000
6	-5.428802000	-0.762398000	0.517082000
6	-4.444531000	1.314120000	-0.209223000
6	-5.525324000	0.419880000	-0.233102000
1	-2.456837000	1.720862000	0.518882000
1	-6.265593000	-1.453176000	0.497461000
1	-4.478522000	2.231841000	-0.788883000
1	-4.254481000	-1.957584000	1.843237000
6	-6.773597000	0.663320000	-1.021632000
8	-7.687529000	-0.149256000	-1.013645000
6	-6.889289000	1.943400000	-1.835494000
1	-6.807228000	2.828579000	-1.193346000
1	-7.857539000	1.950718000	-2.339100000
1	-6.090406000	2.008711000	-2.583986000
6	-1.579859000	-2.717542000	-2.108490000
1	-2.087060000	-1.747728000	-2.161630000
1	-1.351600000	-3.040197000	-3.133069000
1	-2.288520000	-3.434676000	-1.681171000
6	0.501724000	-3.941655000	-1.291704000
1	-0.113022000	-4.806907000	-1.027876000
1	0.899967000	-4.127666000	-2.298137000
1	1.343000000	-3.916411000	-0.591716000

93

Ca’ SCF Done: E(RB3LYP) = -1915.6083652

6	-2.676577000	-1.129269000	1.262236000
1	-1.866907000	-1.797544000	1.577683000
6	-3.975115000	-1.948282000	1.111056000
1	-4.809566000	-1.288189000	0.843040000
1	-3.892052000	-2.692498000	0.311125000
6	-4.312490000	-2.652934000	2.438790000
1	-5.241849000	-3.226553000	2.327787000
1	-3.519879000	-3.378906000	2.670577000
6	-4.440107000	-1.648362000	3.593466000
1	-5.315617000	-1.005907000	3.415138000
1	-4.628052000	-2.176064000	4.537135000
6	-3.185561000	-0.770034000	3.714405000
1	-2.331796000	-1.389604000	4.023247000
1	-3.324670000	-0.013924000	4.497819000
6	-2.842190000	-0.079877000	2.381647000
1	-1.926865000	0.509624000	2.495164000
1	-3.649104000	0.620477000	2.127404000
6	-3.010824000	0.807977000	-1.170133000
1	-2.471090000	1.002236000	-2.106078000
6	-4.425320000	0.323331000	-1.557521000
1	-4.386065000	-0.624537000	-2.106070000
1	-5.015279000	0.140356000	-0.651357000
6	-5.147246000	1.382505000	-2.411513000
1	-4.611371000	1.504065000	-3.364472000
1	-6.154955000	1.027681000	-2.663116000
6	-5.221314000	2.736412000	-1.690820000
1	-5.696808000	3.485244000	-2.336806000
1	-5.862961000	2.636782000	-0.802628000
6	-3.827652000	3.212426000	-1.255785000
1	-3.903915000	4.147747000	-0.686937000
1	-3.226010000	3.439372000	-2.148320000
6	-3.100881000	2.151064000	-0.410827000
1	-2.101814000	2.503387000	-0.134342000
1	-3.656091000	2.005647000	0.524482000
6	-1.849490000	-1.923179000	-1.512313000
1	-2.899888000	-2.131003000	-1.762410000
6	-1.237338000	-3.199566000	-0.893385000
1	-0.229669000	-2.973640000	-0.533710000
1	-1.805399000	-3.504881000	-0.008130000
6	-1.218306000	-4.358985000	-1.904960000
1	-2.251439000	-4.652983000	-2.145818000
1	-0.746494000	-5.235069000	-1.441969000
6	-0.489456000	-3.982037000	-3.201441000
1	0.572721000	-3.799806000	-2.981576000
1	-0.524813000	-4.813505000	-3.916906000
6	-1.099940000	-2.718601000	-3.820373000
1	-2.130136000	-2.931039000	-4.143142000
1	-0.545484000	-2.422396000	-4.720012000
6	-1.110570000	-1.552520000	-2.817546000
1	-1.563513000	-0.672138000	-3.289327000
1	-0.074725000	-1.281429000	-2.570844000
15	-1.878049000	-0.449690000	-0.313531000
28	0.198546000	0.147052000	0.300385000
6	0.486290000	1.819203000	-0.591857000
6	1.174487000	2.755439000	0.100399000
6	0.113056000	2.001267000	-2.051085000
1	-0.881313000	2.446465000	-2.184585000

1	0.820364000	2.643236000	-2.586949000
1	0.105292000	1.041574000	-2.577495000
6	1.657768000	2.431740000	1.510461000
1	0.924076000	2.755700000	2.259262000
1	2.561198000	3.016503000	1.730562000
6	1.990197000	0.960401000	1.726564000
6	1.364918000	0.301395000	2.726295000
9	0.218796000	-1.521741000	1.098251000
9	1.685231000	-0.881338000	3.193254000
9	0.363308000	0.808527000	3.436651000
6	3.187727000	0.343728000	1.073694000
6	4.325613000	1.125033000	0.796836000
6	3.221139000	-1.022568000	0.733761000
6	5.453411000	0.564008000	0.211684000
6	4.353877000	-1.578307000	0.145059000
6	5.487875000	-0.797741000	-0.124362000
1	2.337067000	-1.628964000	0.902682000
1	6.332623000	1.166319000	0.006923000
1	4.341062000	-2.633849000	-0.109681000
1	4.339383000	2.178592000	1.053927000
6	6.730369000	-1.346708000	-0.752453000
8	7.693006000	-0.624749000	-0.970646000
6	6.777521000	-2.823365000	-1.115306000
1	6.627433000	-3.452780000	-0.229896000
1	7.751787000	-3.044144000	-1.554926000
1	5.989072000	-3.078611000	-1.833628000
6	1.561895000	4.175792000	-0.354556000
1	1.850860000	4.708580000	0.564255000
6	0.410611000	5.004242000	-0.954175000
6	2.808675000	4.195802000	-1.264423000
1	3.645041000	3.657097000	-0.806087000
1	2.610615000	3.725009000	-2.233084000
1	3.133338000	5.226825000	-1.454767000
1	0.098688000	4.643840000	-1.938150000
1	0.722717000	6.049667000	-1.073006000
1	-0.466718000	4.988230000	-0.297141000

93

TSCa-C1a SCF Done: E(RB3LYP) = -1915.5838314

6	-1.014960000	2.348846000	-0.429762000
1	-0.117765000	2.478713000	0.183288000
6	-1.946981000	3.557428000	-0.220348000
1	-2.840335000	3.460116000	-0.852515000
1	-2.299454000	3.611164000	0.817114000
6	-1.220999000	4.865721000	-0.587595000
1	-1.899982000	5.718470000	-0.456725000
1	-0.385962000	5.017774000	0.111494000
6	-0.680241000	4.828217000	-2.024784000
1	-1.526863000	4.798604000	-2.727353000
1	-0.125517000	5.749355000	-2.244907000
6	0.211450000	3.598903000	-2.256213000
1	1.119635000	3.684547000	-1.642540000
1	0.540647000	3.560329000	-3.302834000
6	-0.514599000	2.292089000	-1.889492000
1	0.160387000	1.441184000	-2.021762000
1	-1.353856000	2.147696000	-2.581346000
6	-3.374368000	0.336078000	-0.376758000
1	-3.514563000	-0.721975000	-0.121392000
6	-4.535445000	1.109211000	0.287796000
1	-4.506256000	0.998762000	1.376838000
1	-4.443574000	2.181975000	0.077622000
6	-5.898089000	0.606046000	-0.226695000
1	-6.040424000	-0.435862000	0.096573000
1	-6.704271000	1.188445000	0.237669000
6	-5.999859000	0.678069000	-1.756660000
1	-6.960502000	0.268675000	-2.093953000
1	-5.980728000	1.732004000	-2.071693000
6	-4.835905000	-0.067352000	-2.424415000
1	-4.887400000	0.040728000	-3.515379000
1	-4.920215000	-1.143403000	-2.212159000
6	-3.479283000	0.448204000	-1.914445000
1	-2.660386000	-0.094667000	-2.402709000
1	-3.372938000	1.499897000	-2.209674000
6	-1.840004000	0.984934000	2.104715000
1	-2.683868000	1.681692000	2.199703000
6	-0.619714000	1.629001000	2.798004000
1	0.269544000	1.018157000	2.618213000
1	-0.405215000	2.605695000	2.350295000
6	-0.866831000	1.811622000	4.305631000
1	-1.677423000	2.540409000	4.459723000
1	0.029809000	2.240805000	4.770540000
6	-1.244419000	0.491895000	4.992057000
1	-0.392872000	-0.202443000	4.938230000
1	-1.447735000	0.657867000	6.057757000
6	-2.461133000	-0.146929000	4.310559000
1	-3.341539000	0.495868000	4.460632000
1	-2.699119000	-1.114212000	4.772003000
6	-2.224454000	-0.338222000	2.803163000
1	-3.122607000	-0.773654000	2.346158000
1	-1.412434000	-1.063177000	2.650809000
15	-1.599431000	0.678678000	0.243254000
28	0.225313000	-0.802260000	-0.056023000
6	-0.469624000	-2.637712000	-0.337894000
6	0.318161000	-3.422196000	0.435075000
6	-1.820635000	-2.967166000	-0.957245000
1	-2.151772000	-2.064022000	-1.479030000
6	-0.018540000	-4.731997000	1.098387000
1	-1.011797000	-5.106356000	0.854975000
1	0.717557000	-5.495778000	0.810579000
1	0.049234000	-4.633869000	2.191302000
6	1.765995000	-2.976994000	0.604346000
1	2.436754000	-3.824271000	0.377991000
1	1.974810000	-2.690009000	1.643523000
6	1.977796000	-1.799157000	-0.335339000

6	1.203536000	-1.994770000	-1.521867000
9	1.079892000	0.683702000	0.551725000
9	1.190041000	-1.042575000	-2.486842000
9	1.203988000	-3.169597000	-2.205810000
6	3.202401000	-0.976539000	-0.250617000
6	4.193240000	-1.312044000	0.696745000
6	3.452673000	0.134717000	-1.081982000
6	5.370110000	-0.583123000	0.803632000
6	4.633260000	0.858337000	-0.976222000
6	5.613500000	0.516664000	-0.030691000
1	2.712838000	0.443168000	-1.806423000
1	6.126867000	-0.853695000	1.532955000
1	4.782197000	1.708060000	-1.635633000
1	4.050270000	-2.163720000	1.352265000
6	6.894829000	1.267615000	0.129287000
8	7.725492000	0.925220000	0.960061000
6	7.153661000	2.474699000	-0.761300000
1	7.151451000	2.191805000	-1.820903000
1	8.126354000	2.896971000	-0.502646000
1	6.378328000	3.238473000	-0.626297000
6	-2.916509000	-3.302471000	0.080155000
1	-2.903471000	-2.609094000	0.925832000
1	-3.904691000	-3.250372000	-0.393279000
1	-2.807591000	-4.314421000	0.481156000
6	-1.733972000	-4.082344000	-2.021716000
1	-1.396425000	-5.027763000	-1.581826000
1	-2.721048000	-4.253116000	-2.468916000
1	-1.036881000	-3.819622000	-2.820747000

93

TSCa'-C1a' SCF Done: E(RB3LYP) = -1915.5857493

6	1.325062000	-2.457005000	-0.202970000
1	0.426149000	-2.590384000	0.407685000
6	2.366772000	-3.521245000	0.188556000
1	3.263797000	-3.416041000	-0.436674000
1	2.690614000	-3.395122000	1.229214000
6	1.791997000	-4.936719000	-0.009124000
1	2.550706000	-5.686762000	0.249792000
1	0.954145000	-5.082647000	0.687962000
6	1.298806000	-5.149563000	-1.448263000
1	2.160814000	-5.124637000	-2.132105000
1	0.849299000	-6.145571000	-1.550974000
6	0.292961000	-4.064687000	-1.862497000
1	-0.620505000	-4.164066000	-1.258880000
1	-0.004873000	-4.202456000	-2.910069000
6	0.866661000	-2.650227000	-1.664784000
1	0.110044000	-1.901635000	-1.920238000
1	1.707700000	-2.508766000	-2.355976000
6	3.473958000	-0.213238000	-0.379204000
1	3.506900000	0.872620000	-0.212179000
6	4.683626000	-0.810266000	0.373583000
1	4.612987000	-0.612715000	1.448256000
1	4.701649000	-1.900329000	0.253860000
6	6.004892000	-0.219187000	-0.155228000
1	6.036289000	0.854799000	0.081726000
1	6.850360000	-0.678344000	0.373060000
6	6.156610000	-0.407667000	-1.671085000
1	7.080423000	0.068798000	-2.022762000
1	6.253627000	-1.480526000	-1.894306000
6	4.943501000	0.155217000	-2.425537000
1	5.036751000	-0.043052000	-3.500931000
1	4.915237000	1.248793000	-2.310109000
6	3.629814000	-0.445340000	-1.897438000
1	2.774472000	-0.028488000	-2.441591000
1	3.631420000	-1.524286000	-2.099197000
6	1.914247000	-0.646161000	2.118037000
1	2.832236000	-1.206466000	2.341353000
6	0.754056000	-1.326013000	2.876641000
1	-0.194939000	-0.867413000	2.583914000
1	0.679831000	-2.379458000	2.584139000
6	0.959827000	-1.242294000	4.398942000
1	1.846392000	-1.829753000	4.683285000
1	0.104074000	-1.705203000	4.906598000
6	1.144176000	0.204473000	4.876565000
1	0.216304000	0.767232000	4.696205000
1	1.322487000	0.231413000	5.959193000
6	2.300357000	0.882148000	4.129296000
1	3.246358000	0.388759000	4.398428000
1	2.397496000	1.931225000	4.438105000
6	2.104563000	0.808018000	2.605780000
1	2.958553000	1.279480000	2.101687000
1	1.216438000	1.390761000	2.323933000
15	1.719463000	-0.658235000	0.227608000
28	-0.202504000	0.581757000	-0.297153000
6	0.411245000	2.366323000	-0.833149000
6	-0.345540000	3.230973000	-0.120476000
6	1.660595000	2.670222000	-1.612468000
1	1.725783000	2.022383000	-2.492621000
1	1.685410000	3.705061000	-1.971274000
1	2.568179000	2.500754000	-1.022820000
6	-0.001644000	4.647477000	0.324852000
1	-0.691451000	4.879020000	1.149969000
6	-1.775336000	2.782227000	0.162101000
1	-2.472094000	3.586802000	-0.131950000
1	-1.943055000	2.610476000	1.233978000
6	-2.000845000	1.500105000	-0.630617000
6	-1.270141000	1.545996000	-1.857098000
9	-0.965506000	-0.873135000	0.479143000
9	-1.286501000	0.471132000	-2.684959000
9	-1.266696000	2.615539000	-2.694873000
6	-3.203810000	0.673126000	-0.399744000
6	-4.194419000	1.138105000	0.491788000
6	-3.434553000	-0.564052000	-1.036244000
6	-5.356906000	0.415465000	0.724318000

6	-4.600876000	-1.281815000	-0.804693000
6	-5.583846000	-0.808270000	0.079200000
1	-2.690058000	-0.974728000	-1.702492000
1	-6.115517000	0.787155000	1.405596000
1	-4.735282000	-2.230315000	-1.316048000
1	-4.063749000	2.087119000	0.999839000
6	-6.852841000	-1.542860000	0.364433000
8	-7.684231000	-1.086007000	1.137454000
6	-7.098432000	-2.877264000	-0.325085000
1	-7.098479000	-2.764792000	-1.416024000
1	-8.066386000	-3.264541000	-0.001903000
1	-6.314708000	-3.601591000	-0.072411000
6	1.422072000	4.823213000	0.878192000
1	1.540237000	5.829516000	1.297213000
1	1.628627000	4.099204000	1.674161000
1	2.184640000	4.698777000	0.103490000
6	-0.303316000	5.662232000	-0.801515000
1	0.375333000	5.528708000	-1.650704000
1	-1.327700000	5.555515000	-1.174039000
1	-0.182370000	6.687618000	-0.432025000

93

C1a SCF Done: E(RB3LYP) = -1915.6494875

6	1.627605000	-1.564897000	-1.587831000
1	0.673649000	-2.080092000	-1.429867000
6	2.709465000	-2.606822000	-1.930344000
1	3.674743000	-2.109528000	-2.100271000
1	2.858866000	-3.303809000	-1.096599000
6	2.324409000	-3.390605000	-3.199229000
1	3.117652000	-4.107196000	-3.449145000
1	1.420411000	-3.981602000	-2.992606000
6	2.056371000	-2.453396000	-4.386153000
1	2.993741000	-1.949948000	-4.668027000
1	1.739601000	-3.033979000	-5.261972000
6	0.999020000	-1.395027000	-4.035750000
1	0.030688000	-1.887115000	-3.866666000
1	0.857875000	-0.704642000	-4.877326000
6	1.384013000	-0.604121000	-2.773244000
1	0.586239000	0.103549000	-2.521351000
1	2.284498000	-0.014717000	-2.986862000
6	3.534040000	0.037630000	0.283253000
1	3.401515000	0.765128000	1.099336000
6	4.651090000	-0.933777000	0.724990000
1	4.372816000	-1.453811000	1.647084000
1	4.798241000	-1.706884000	-0.040755000
6	5.980348000	-0.191355000	0.959306000
1	5.863977000	0.490362000	1.815072000
1	6.757883000	-0.912887000	1.240942000
6	6.417169000	0.613498000	-0.271431000
1	7.339463000	1.167855000	-0.056785000
1	6.651853000	-0.078621000	-1.093671000
6	5.307726000	1.574108000	-0.721251000
1	5.606456000	2.103331000	-1.635186000
1	5.156439000	2.343340000	0.050173000
6	3.986039000	0.826192000	-0.965805000
1	3.206664000	1.527859000	-1.284501000
1	4.131529000	0.127555000	-1.798671000
6	1.710872000	-2.076410000	1.365394000
1	2.665524000	-2.614199000	1.298412000
6	0.577252000	-3.097610000	1.127149000
1	-0.378615000	-2.570043000	1.059921000
1	0.716183000	-3.594926000	0.160429000
6	0.542916000	-4.165704000	2.234280000
1	1.459101000	-4.774311000	2.186989000
1	-0.295175000	-4.849745000	2.050817000
6	0.427054000	-3.544408000	3.632375000
1	-0.540549000	-3.028944000	3.721020000
1	0.438810000	-4.326213000	4.402405000
6	1.560108000	-2.539236000	3.876953000
1	2.522648000	-3.071900000	3.897321000
1	1.447694000	-2.061030000	4.858788000
6	1.597596000	-1.462859000	2.778935000
1	2.425153000	-0.766469000	2.970620000
1	0.672372000	-0.870167000	2.826544000
15	1.770693000	-0.677755000	0.076267000
28	-0.256057000	0.564041000	0.278973000
6	-0.146919000	2.809816000	0.334061000
6	-0.169160000	2.319183000	1.630034000
6	0.856667000	3.644629000	-0.443593000
1	1.021606000	3.116380000	-1.392939000
6	0.826856000	2.452563000	2.747479000
1	1.857889000	2.561767000	2.415263000
1	0.574799000	3.332517000	3.356647000
1	0.767152000	1.582037000	3.409035000
6	-1.571470000	1.803458000	1.961406000
1	-2.180151000	2.590907000	2.441726000
1	-1.544395000	0.947303000	2.645017000
6	-2.018929000	1.436666000	0.547920000
6	-1.535840000	2.598625000	-0.256897000
9	-0.960673000	-0.972779000	-0.315263000
9	-1.475094000	2.382718000	-1.623024000
9	-2.322699000	3.747385000	-0.136007000
6	-3.280172000	0.732818000	0.281064000
6	-4.098046000	0.292361000	1.342392000
6	-3.705141000	0.449305000	-1.033123000
6	-5.283190000	-0.389868000	1.104257000
6	-4.894187000	-0.228666000	-1.269405000
6	-5.704534000	-0.661968000	-0.206508000
1	-3.090384000	0.760459000	-1.868107000
1	-5.911679000	-0.721414000	1.924637000
1	-5.186637000	-0.426518000	-2.296408000
1	-3.805199000	0.496496000	2.368162000
6	-6.989244000	-1.396073000	-0.405326000
8	-7.666930000	-1.752575000	0.549730000

6	-7.449040000	-1.698369000	-1.824809000
1	-7.578543000	-0.775931000	-2.403617000
1	-8.400183000	-2.231753000	-1.777407000
1	-6.713186000	-2.314927000	-2.355014000
6	2.222454000	3.852656000	0.223566000
1	2.706125000	2.910060000	0.489172000
1	2.886260000	4.381429000	-0.469591000
1	2.138503000	4.466336000	1.127374000
6	0.252122000	5.024430000	-0.802643000
1	0.019749000	5.596682000	0.102953000
1	0.982329000	5.595548000	-1.387104000
1	-0.663091000	4.933681000	-1.388683000

93

C1a' SCF Done: E(RB3LYP) = -1915.649941

6	1.610710000	-2.129216000	-0.917127000
1	0.656858000	-2.528820000	-0.554924000
6	2.688293000	-3.224526000	-0.812319000
1	3.648797000	-2.843829000	-1.186601000
1	2.851480000	-3.517793000	0.232287000
6	2.289849000	-4.459453000	-1.642140000
1	3.078567000	-5.220455000	-1.578606000
1	1.385230000	-4.906862000	-1.205377000
6	2.017537000	-4.091588000	-3.108376000
1	2.955807000	-3.757132000	-3.576755000
1	1.689848000	-4.977890000	-3.666544000
6	0.969719000	-2.973664000	-3.218650000
1	-0.000566000	-3.345216000	-2.859412000
1	0.827027000	-2.687619000	-4.268721000
6	1.364681000	-1.737245000	-2.391853000
1	0.571947000	-0.983498000	-2.452051000
1	2.268804000	-1.292000000	-2.827970000
6	3.536698000	0.085040000	0.135205000
1	3.433338000	1.025858000	0.696023000
6	4.681262000	-0.704310000	0.808562000
1	4.441259000	-0.926760000	1.853519000
1	4.818704000	-1.668941000	0.304827000
6	6.002095000	0.087847000	0.760312000
1	5.897551000	0.995377000	1.373655000
1	6.803382000	-0.505925000	1.218460000
6	6.384766000	0.487417000	-0.671413000
1	7.302121000	1.089473000	-0.666281000
1	6.608742000	-0.419014000	-1.253224000
6	5.244474000	1.255426000	-1.354300000
1	5.505811000	1.482851000	-2.395709000
1	5.102032000	2.222176000	-0.848860000
6	3.930184000	0.457124000	-1.310807000
1	3.125104000	1.021433000	-1.795879000
1	4.061281000	-0.460895000	-1.898434000
6	1.695220000	-1.339109000	1.986229000
1	2.641988000	-1.868462000	2.156557000
6	0.548044000	-2.346007000	2.218703000
1	-0.404376000	-1.887370000	1.938018000
1	0.672028000	-3.212159000	1.558674000
6	0.518159000	-2.829997000	3.678933000
1	1.428504000	-3.410879000	3.893073000
1	-0.327919000	-3.515065000	3.816218000
6	0.424355000	-1.662497000	4.670957000
1	-0.536528000	-1.145748000	4.531754000
1	0.435476000	-2.034146000	5.703482000
6	1.570778000	-0.666923000	4.450936000
1	2.527612000	-1.152684000	4.693874000
1	1.474469000	0.190053000	5.130318000
6	1.608283000	-0.171629000	2.995624000
1	2.450138000	0.521628000	2.863463000
1	0.692932000	0.401906000	2.787281000
15	1.769080000	-0.629907000	0.223769000
28	-0.238459000	0.601290000	-0.160358000
6	-0.120067000	2.557652000	-1.215461000
6	-0.136498000	2.821589000	0.143340000
6	0.859329000	2.888324000	-2.304592000
1	0.817973000	2.121546000	-3.083331000
1	0.566287000	3.837456000	-2.773879000
1	1.886750000	2.983083000	-1.958920000
6	0.850610000	3.534826000	1.049046000
1	0.984530000	2.882206000	1.924574000
6	-1.539144000	2.530732000	0.690840000
1	-2.154123000	3.446731000	0.713003000
1	-1.510211000	2.132124000	1.711925000
6	-1.999331000	1.510842000	-0.346737000
6	-1.507032000	2.098292000	-1.629002000
9	-0.956262000	-1.031680000	-0.005269000
9	-1.450469000	1.212816000	-2.692213000
9	-2.248841000	3.168890000	-2.129428000
6	-3.266387000	0.780449000	-0.213329000
6	-4.060591000	0.921724000	0.943921000
6	-3.722017000	-0.101483000	-1.214852000
6	-5.251755000	0.224931000	1.091474000
6	-4.917278000	-0.793792000	-1.067476000
6	-5.703390000	-0.645548000	0.087524000
1	-3.126366000	-0.242873000	-2.107551000
1	-5.861697000	0.342462000	1.981471000
1	-5.234083000	-1.463031000	-1.861858000
1	-3.744666000	1.596035000	1.734403000
6	-6.992169000	-1.369053000	0.297530000
8	-7.647714000	-1.207499000	1.318724000
6	-7.484618000	-2.319560000	-0.785212000
1	-7.635084000	-1.792561000	-1.735176000
1	-8.430646000	-2.757256000	-0.461353000
1	-6.757696000	-3.119922000	-0.968683000
6	2.240991000	3.833185000	0.473036000
1	2.877654000	4.251268000	1.261006000
1	2.733691000	2.936279000	0.090641000
1	2.193949000	4.571906000	-0.334446000

6	0.218220000	4.849438000	1.567761000
1	-0.002973000	5.531651000	0.738214000
1	-0.709740000	4.673337000	2.119125000
1	0.917646000	5.354765000	2.242888000

94

Ab SCF Done: E(RB3LYP) = -2028.7165089

6	-1.676125000	2.203767000	0.740016000
1	-0.712787000	2.727715000	0.708654000
6	-2.406706000	2.648051000	2.022730000
1	-3.394753000	2.170856000	2.072536000
1	-1.858785000	2.331428000	2.918322000
6	-2.590122000	4.177621000	2.043300000
1	-3.136747000	4.474924000	2.947821000
1	-1.601422000	4.655707000	2.103621000
6	-3.320266000	4.679988000	0.788597000
1	-4.354427000	4.303582000	0.798823000
1	-3.387760000	5.775362000	0.803227000
6	-2.623799000	4.204439000	-0.495446000
1	-1.633983000	4.675128000	-0.577272000
1	-3.195466000	4.523324000	-1.376884000
6	-2.449912000	2.676094000	-0.511916000
1	-1.917895000	2.370721000	-1.418808000
1	-3.439207000	2.204609000	-0.548194000
6	-2.569359000	-0.753672000	1.051643000
1	-2.185714000	-1.734311000	0.741523000
6	-2.992257000	-0.885050000	2.532203000
1	-2.139068000	-1.154690000	3.162643000
1	-3.369864000	0.075214000	2.906398000
6	-4.085192000	-1.957590000	2.704177000
1	-3.665352000	-2.940368000	2.443951000
1	-4.381221000	-2.013536000	3.759886000
6	-5.307129000	-1.680832000	1.818541000
1	-6.048772000	-2.482191000	1.930691000
1	-5.795111000	-0.753042000	2.153135000
6	-4.893474000	-1.534077000	0.348280000
1	-5.764667000	-1.281166000	-0.270565000
1	-4.511665000	-2.495776000	-0.021811000
6	-3.807184000	-0.457948000	0.175431000
1	-3.517584000	-0.380410000	-0.877576000
1	-4.232774000	0.511824000	0.463998000
6	-0.000474000	0.211014000	2.177930000
1	-0.671287000	0.302298000	3.042053000
6	1.082955000	1.297533000	2.326357000
1	1.751293000	1.277134000	1.459877000
1	0.632344000	2.295576000	2.353528000
6	1.910832000	1.085038000	3.607520000
1	1.263017000	1.221808000	4.486695000
1	2.688650000	1.856989000	3.671479000
6	2.541453000	-0.313474000	3.655362000
1	3.277218000	-0.406263000	2.843791000
1	3.088753000	-0.453549000	4.596523000
6	1.473824000	-1.403569000	3.492978000
1	0.804015000	-1.391399000	4.366048000
1	1.940716000	-2.396584000	3.470550000
6	0.644975000	-1.191660000	2.214060000
1	-0.117797000	-1.976056000	2.133839000
1	1.299898000	-1.300549000	1.338940000
15	-1.093203000	0.396426000	0.629479000
28	-0.224546000	0.118785000	-1.568227000
6	-1.296371000	-1.540730000	-2.160836000
6	-1.509824000	-0.627714000	-2.986064000
6	-2.000357000	0.050243000	-4.199808000
1	-2.818780000	-0.524602000	-4.651228000
1	-2.371512000	1.058003000	-3.981779000
1	-1.203758000	0.146448000	-4.946433000
6	0.967696000	0.677521000	-3.022165000
1	0.461890000	1.352665000	-3.707059000
1	1.451276000	-0.168918000	-3.505170000
6	1.524360000	1.222566000	-1.826404000
6	1.399208000	2.709573000	-1.668196000
9	1.372280000	3.122542000	-0.368668000
9	2.434941000	3.388166000	-2.237698000
9	0.272019000	3.201818000	-2.243925000
6	2.691099000	0.556585000	-1.183399000
6	2.856332000	-0.844479000	-1.299655000
6	3.686377000	1.257841000	-0.471869000
6	3.942225000	-1.499377000	-0.740261000
6	4.778022000	0.597543000	0.087607000
6	4.928888000	-0.791351000	-0.032296000
1	3.622788000	2.332801000	-0.362243000
1	4.053977000	-2.574665000	-0.838336000
1	5.522135000	1.185264000	0.617335000
1	2.110469000	-1.426029000	-1.832539000
8	6.151817000	-2.763961000	0.430775000
6	6.077991000	-1.547637000	0.546474000
6	7.164687000	-0.779477000	1.285253000
1	7.635570000	-0.034991000	0.632080000
1	6.752429000	-0.242229000	2.147942000
1	7.920395000	-1.487855000	1.629288000
6	-1.409644000	-2.864368000	-1.594045000
6	-2.522233000	-3.669005000	-1.918396000
6	-0.419996000	-3.395367000	-0.746387000
6	-2.637877000	-4.958615000	-1.405140000
6	-0.541916000	-4.687005000	-0.234937000
6	-1.650030000	-5.472440000	-0.559543000
1	-3.289333000	-3.270429000	-2.575691000
1	0.443367000	-2.787594000	-0.496846000
1	-3.501053000	-5.565024000	-1.666628000
1	0.234284000	-5.081559000	0.415149000
1	-1.742366000	-6.478889000	-0.160876000

94

Ab’ SCF Done: E(RB3LYP) = -2028.7154623

6	-0.933331000	-1.354350000	1.954570000
1	0.025467000	-1.015041000	2.368857000
6	-1.194359000	-2.779292000	2.480902000
1	-2.164413000	-3.138793000	2.111035000
1	-0.437406000	-3.481119000	2.111273000
6	-1.210007000	-2.799576000	4.021262000
1	-1.425427000	-3.815327000	4.377883000
1	-0.206683000	-2.543772000	4.392633000
6	-2.231098000	-1.804354000	4.592101000
1	-3.245586000	-2.136521000	4.324448000
1	-2.183791000	-1.799995000	5.688619000
6	-2.002751000	-0.389240000	4.040414000
1	-1.040773000	-0.000622000	4.403933000
1	-2.777200000	0.293488000	4.413308000
6	-1.993386000	-0.373854000	2.501752000
1	-1.788993000	0.638667000	2.144574000
1	-2.990354000	-0.647265000	2.135816000
6	-1.995444000	-2.025042000	-0.888931000
1	-1.820359000	-1.638944000	-1.904026000
6	-1.989484000	-3.566340000	-0.978430000
1	-1.016482000	-3.940946000	-1.312069000
1	-2.169477000	-3.999629000	0.013941000
6	-3.072789000	-4.069280000	-1.952367000
1	-2.821158000	-3.734619000	-2.969946000
1	-3.065593000	-5.166781000	-1.976136000
6	-4.468946000	-3.551332000	-1.580840000
1	-5.206900000	-3.880065000	-2.323814000
1	-4.774595000	-3.992727000	-0.620441000
6	-4.476196000	-2.021338000	-1.455550000
1	-5.462798000	-1.668173000	-1.129449000
1	-4.292981000	-1.570570000	-2.442125000
6	-3.399078000	-1.534435000	-0.471417000
1	-3.425261000	-0.442828000	-0.399642000
1	-3.636291000	-1.925092000	0.526830000
6	0.877963000	-2.323932000	-0.177066000
1	0.518793000	-3.341913000	0.025109000
6	2.059310000	-2.051393000	0.777383000
1	2.408265000	-1.019673000	0.652238000
1	1.742836000	-2.151978000	1.821554000
6	3.225605000	-3.022154000	0.514615000
1	2.909333000	-4.045026000	0.769609000
1	4.059928000	-2.778287000	1.184740000
6	3.689481000	-2.984878000	-0.947740000
1	4.121689000	-1.998759000	-1.165802000
1	4.485673000	-3.721727000	-1.113737000
6	2.517318000	-3.243987000	-1.903916000
1	2.159184000	-4.276474000	-1.774760000
1	2.847969000	-3.156099000	-2.946846000
6	1.356440000	-2.268344000	-1.643950000
1	0.528882000	-2.493221000	-2.329690000
1	1.688833000	-1.245956000	-1.870297000
15	-0.600902000	-1.154220000	0.092825000
28	-0.353434000	1.110763000	-0.607187000
6	-1.223625000	1.028244000	-2.524403000
6	-2.060746000	1.569569000	-1.786990000
6	-0.470021000	0.522674000	-3.680744000
1	-0.130737000	-0.507319000	-3.528823000
1	-1.093771000	0.548120000	-4.583554000
1	0.419600000	1.135756000	-3.869721000
6	0.354104000	2.915532000	-0.527694000
1	-0.406168000	3.605296000	-0.168578000
1	0.834790000	3.238931000	-1.449051000
6	1.104032000	2.148703000	0.423520000
6	0.758160000	2.364675000	1.866563000
9	0.971105000	1.267988000	2.650024000
9	1.476330000	3.366224000	2.446245000
9	-0.550752000	2.704027000	2.043895000
6	2.498789000	1.726047000	0.117981000
6	2.898839000	1.512663000	-1.222692000
6	3.486362000	1.544583000	1.109540000
6	4.195522000	1.151532000	-1.550683000
6	4.789199000	1.180830000	0.777979000
6	5.172935000	0.977675000	-0.555060000
1	3.247855000	1.702082000	2.153391000
1	4.484476000	0.993935000	-2.585160000
1	5.512534000	1.063023000	1.579686000
1	2.169027000	1.624210000	-2.018057000
8	6.829365000	0.394136000	-2.141525000
6	6.553319000	0.588325000	-0.964582000
6	7.621789000	0.435779000	0.109015000
1	7.755799000	1.368755000	0.669479000
1	7.347282000	-0.342049000	0.831837000
1	8.563213000	0.164001000	-0.371744000
6	-3.273401000	2.210101000	-1.343191000
6	-4.373316000	2.274305000	-2.224646000
6	-3.398539000	2.774588000	-0.060863000
6	-5.557688000	2.891014000	-1.831939000
6	-4.588145000	3.392090000	0.323405000
6	-5.670273000	3.453061000	-0.556702000
1	-4.284597000	1.837730000	-3.215092000
1	-2.559255000	2.729505000	0.623475000
1	-6.395236000	2.933944000	-2.523121000
1	-4.668110000	3.826981000	1.316010000
1	-6.595706000	3.934367000	-0.252657000

94

TSAb-Bb SCF Done: E(RB3LYP) = -2028.7023822

6	1.705035000	-2.100474000	-0.873226000
1	0.657463000	-2.248661000	-1.166253000
6	2.241163000	-3.453635000	-0.353777000
1	3.292398000	-3.356049000	-0.055995000
1	1.692540000	-3.783249000	0.534357000
6	2.130565000	-4.535942000	-1.445415000
1	2.529570000	-5.484551000	-1.063312000

1	1.068037000	-4.710808000	-1.669564000
6	2.859731000	-4.126826000	-2.733089000
1	3.940621000	-4.071777000	-2.533812000
1	2.724192000	-4.894061000	-3.505991000
6	2.367457000	-2.764342000	-3.239099000
1	1.321456000	-2.843974000	-3.562173000
1	2.944212000	-2.453448000	-4.119715000
6	2.473657000	-1.684894000	-2.148074000
1	2.101150000	-0.730395000	-2.531361000
1	3.534586000	-1.543823000	-1.902501000
6	3.273808000	0.008546000	0.775764000
1	3.065530000	0.625091000	1.661307000
6	4.353483000	-1.019554000	1.171780000
1	3.988267000	-1.692212000	1.957018000
1	4.599454000	-1.648573000	0.306455000
6	5.636221000	-0.313331000	1.648928000
1	5.420656000	0.239415000	2.575270000
1	6.397829000	-1.061886000	1.902452000
6	6.174148000	0.658909000	0.589547000
1	7.061114000	1.181546000	0.969374000
1	6.501821000	0.087829000	-0.291916000
6	5.098966000	1.670567000	0.165125000
1	5.479726000	2.320710000	-0.632830000
1	4.864886000	2.326763000	1.016962000
6	3.811922000	0.970685000	-0.307132000
1	3.046210000	1.711949000	-0.561725000
1	4.028772000	0.416524000	-1.227743000
6	1.022831000	-1.539336000	1.979868000
1	1.781836000	-2.307968000	2.181918000
6	-0.345272000	-2.234573000	1.806945000
1	-1.101513000	-1.480826000	1.557574000
1	-0.325857000	-2.937833000	0.966582000
6	-0.770649000	-2.974799000	3.086655000
1	-0.071496000	-3.802348000	3.280679000
1	-1.758298000	-3.427073000	2.934352000
6	-0.796013000	-2.034702000	4.299129000
1	-1.586089000	-1.283399000	4.155939000
1	-1.053880000	-2.590608000	5.209315000
6	0.553149000	-1.324235000	4.473328000
1	1.323448000	-2.064636000	4.736286000
1	0.507102000	-0.612875000	5.307933000
6	0.980202000	-0.585353000	3.191868000
1	1.958603000	-0.118087000	3.355551000
1	0.271439000	0.227488000	2.984025000
15	1.546292000	-0.670474000	0.376690000
28	0.150634000	0.938853000	-0.178414000
6	0.618506000	2.455581000	0.816843000
6	-0.388514000	2.946898000	0.141921000
6	1.511303000	3.049253000	1.855921000
1	1.222323000	4.080006000	2.101944000
1	1.490233000	2.459225000	2.779829000
1	2.554040000	3.066625000	1.516139000
6	-1.399477000	1.989882000	-1.187698000
1	-1.110029000	2.630341000	-2.017380000
1	-2.361337000	2.287887000	-0.781069000
6	-1.307877000	0.533672000	-1.485329000
6	-0.821944000	0.259366000	-2.872629000
9	-0.390258000	-1.023402000	-3.056903000
9	-1.768942000	0.454217000	-3.835305000
9	0.224116000	1.061209000	-3.216518000
6	-2.385286000	-0.335973000	-0.943806000
6	-2.997349000	-0.013580000	0.293514000
6	-2.860283000	-1.493207000	-1.599596000
6	-4.009246000	-0.790864000	0.834365000
6	-3.876696000	-2.270973000	-1.053378000
6	-4.472953000	-1.940583000	0.172589000
1	-2.444173000	-1.786166000	-2.554403000
1	-4.463841000	-0.528385000	1.784665000
1	-4.210781000	-3.145502000	-1.604274000
1	-2.651647000	0.853211000	0.849072000
8	-6.036952000	-2.421002000	1.882966000
6	-5.562527000	-2.739857000	0.799613000
6	-6.084660000	-3.969979000	0.068789000
1	-5.287053000	-4.706232000	-0.088223000
1	-6.877859000	-4.420760000	0.667957000
1	-6.481774000	-3.704936000	-0.918532000
6	-0.992926000	4.291805000	0.093806000
6	-2.292745000	4.540029000	0.567373000
6	-0.240768000	5.371867000	-0.399296000
6	-2.820473000	5.830676000	0.554649000
6	-0.771498000	6.662523000	-0.413006000
6	-2.062779000	6.896253000	0.062851000
1	-2.884153000	3.719831000	0.965987000
1	0.761904000	5.188466000	-0.775172000
1	-3.824246000	6.004454000	0.933250000
1	-0.174795000	7.484917000	-0.798894000
1	-2.476643000	7.900857000	0.050958000

94

TSAb'-Bb' SCF Done: E(RB3LYP) = -2028.7064558

6	-0.497705000	2.652685000	0.121344000
1	0.584995000	2.639330000	-0.057907000
6	-0.773170000	3.673676000	1.245158000
1	-1.854102000	3.747718000	1.420924000
1	-0.319688000	3.360848000	2.192735000
6	-0.237536000	5.065069000	0.854316000
1	-0.457743000	5.783603000	1.654552000
1	0.858089000	5.015968000	0.770302000
6	-0.828796000	5.549773000	-0.477544000
1	-1.907805000	5.723895000	-0.349500000
1	-0.388626000	6.515304000	-0.757725000
6	-0.613205000	4.517900000	-1.594162000
1	0.457834000	4.432505000	-1.820973000
1	-1.103801000	4.850255000	-2.518068000

6	-1.149752000	3.132299000	-1.194696000
1	-0.968380000	2.415180000	-1.998488000
1	-2.236471000	3.201617000	-1.067979000
6	-2.632636000	0.581259000	1.056078000
1	-2.686489000	-0.512040000	1.134877000
6	-3.051840000	1.158660000	2.426225000
1	-2.367078000	0.837319000	3.218094000
1	-3.015134000	2.254984000	2.403530000
6	-4.480496000	0.714038000	2.793991000
1	-4.493214000	-0.377894000	2.923463000
1	-4.761494000	1.148693000	3.762124000
6	-5.496926000	1.106083000	1.713408000
1	-6.496664000	0.741337000	1.981730000
1	-5.566931000	2.203220000	1.663075000
6	-5.078145000	0.557411000	0.342565000
1	-5.777424000	0.895602000	-0.433354000
1	-5.129001000	-0.538772000	0.354781000
6	-3.650552000	0.997260000	-0.028002000
1	-3.366945000	0.577676000	-1.000068000
1	-3.643572000	2.088898000	-0.136460000
6	0.134030000	0.528567000	2.121524000
1	-0.346281000	1.163633000	2.877631000
6	1.617692000	0.939904000	2.042282000
1	2.119378000	0.361513000	1.259664000
1	1.711600000	1.995525000	1.763881000
6	2.331091000	0.706068000	3.386307000
1	1.902322000	1.376076000	4.147001000
1	3.389188000	0.979878000	3.288862000
6	2.199368000	-0.748766000	3.857766000
1	2.738205000	-1.403946000	3.159380000
1	2.674868000	-0.876281000	4.838489000
6	0.726006000	-1.175083000	3.921732000
1	0.214356000	-0.603547000	4.710781000
1	0.645411000	-2.233331000	4.201708000
6	0.007949000	-0.939713000	2.581447000
1	-1.046580000	-1.226175000	2.674476000
1	0.443838000	-1.591784000	1.812513000
15	-0.821889000	0.819800000	0.506604000
28	-0.237630000	-0.589343000	-1.132129000
6	-1.564444000	-1.949498000	-1.219257000
6	-0.795719000	-2.281723000	-2.211625000
6	-0.764128000	-3.380084000	-3.223043000
1	-1.658488000	-4.001149000	-3.102482000
1	-0.748728000	-2.994323000	-4.250249000
1	0.117595000	-4.023052000	-3.105809000
6	0.885371000	-1.315485000	-2.751525000
1	0.515615000	-1.073933000	-3.744273000
1	1.451920000	-2.242359000	-2.738782000
6	1.526272000	-0.200078000	-2.041641000
6	1.571643000	1.061923000	-2.845578000
9	1.764810000	2.184010000	-2.093610000
9	2.576252000	1.077815000	-3.768764000
9	0.424611000	1.264188000	-3.548683000
6	2.712221000	-0.520254000	-1.201965000
6	2.783226000	-1.764041000	-0.527948000
6	3.807492000	0.354237000	-1.035941000
6	3.872580000	-2.107886000	0.257211000
6	4.899498000	0.006047000	-0.245771000
6	4.956846000	-1.228068000	0.418148000
1	3.817698000	1.316043000	-1.531954000
1	3.908878000	-3.065299000	0.767607000
1	5.718327000	0.714148000	-0.156140000
1	1.955032000	-2.462429000	-0.606060000
8	6.085859000	-2.726589000	1.860212000
6	6.098080000	-1.647655000	1.281516000
6	7.286784000	-0.709093000	1.435082000
1	6.979974000	0.250679000	1.868249000
1	8.021565000	-1.180902000	2.089903000
1	7.750036000	-0.494933000	0.464403000
6	-2.792733000	-2.538818000	-0.691529000
6	-3.956604000	-2.594082000	-1.483027000
6	-2.836227000	-3.108878000	0.595253000
6	-5.119037000	-3.199039000	-1.004441000
6	-3.998020000	-3.719198000	1.067679000
6	-5.146176000	-3.765259000	0.272312000
1	-3.940231000	-2.152941000	-2.475835000
1	-1.943776000	-3.082646000	1.213932000
1	-6.005183000	-3.230404000	-1.633352000
1	-4.005329000	-4.162941000	2.060032000
1	-6.051327000	-4.238105000	0.643264000

94

Bb SCF Done: E(RB3LYP) = -2028.7313498

6	-2.921179000	-0.540995000	1.041887000
1	-2.269467000	-1.386556000	1.311794000
6	-4.306617000	-1.125471000	0.697722000
1	-4.999438000	-0.312214000	0.446578000
1	-4.254324000	-1.777203000	-0.181789000
6	-4.873156000	-1.916957000	1.891785000
1	-5.868860000	-2.303856000	1.639736000
1	-4.234433000	-2.793436000	2.075592000
6	-4.938024000	-1.059941000	3.164641000
1	-5.676692000	-0.257203000	3.020998000
1	-5.294082000	-1.662943000	4.009368000
6	-3.573103000	-0.435885000	3.491381000
1	-2.859225000	-1.226409000	3.764006000
1	-3.654889000	0.224059000	4.364129000
6	-3.012559000	0.354833000	2.296802000
1	-2.030523000	0.768325000	2.548711000
1	-3.677150000	1.204217000	2.094195000
6	-2.776597000	1.795071000	-1.047354000
1	-2.098602000	2.062289000	-1.870969000
6	-4.184669000	1.624077000	-1.652707000
1	-4.211316000	0.791178000	-2.364931000

1	-4.903028000	1.383721000	-0.858518000
6	-4.632574000	2.919500000	-2.356422000
1	-3.975575000	3.105004000	-3.218816000
1	-5.645715000	2.790831000	-2.758224000
6	-4.582678000	4.128028000	-1.409614000
1	-4.855486000	5.043624000	-1.949160000
1	-5.334783000	3.996346000	-0.617539000
6	-3.196088000	4.280759000	-0.766146000
1	-3.199217000	5.109542000	-0.047029000
1	-2.459352000	4.540908000	-1.540432000
6	-2.755483000	2.985074000	-0.062556000
1	-1.757116000	3.105105000	0.373651000
1	-3.440951000	2.789490000	0.771523000
6	-2.026684000	-1.013916000	-1.768831000
1	-3.089041000	-1.153431000	-2.011975000
6	-1.444862000	-2.374524000	-1.324624000
1	-0.403847000	-2.234197000	-1.003139000
1	-1.991000000	-2.765295000	-0.458254000
6	-1.487462000	-3.413030000	-2.459255000
1	-2.535718000	-3.644684000	-2.700848000
1	-1.031535000	-4.348626000	-2.112383000
6	-0.773657000	-2.906201000	-3.719216000
1	0.298534000	-2.792192000	-3.505319000
1	-0.855846000	-3.644042000	-4.526894000
6	-1.350502000	-1.557327000	-4.168958000
1	-2.393785000	-1.694688000	-4.490149000
1	-0.802714000	-1.176990000	-5.040306000
6	-1.300788000	-0.514650000	-3.038163000
1	-1.738553000	0.426964000	-3.391288000
1	-0.250662000	-0.304069000	-2.791265000
15	-1.911310000	0.253030000	-0.362763000
28	0.279562000	0.429782000	0.502619000
6	1.360926000	1.768369000	-0.264919000
6	2.610131000	1.922221000	0.236638000
6	0.792540000	2.552776000	-1.420075000
1	0.017165000	3.261457000	-1.099987000
1	1.570158000	3.130157000	-1.936348000
1	0.331875000	1.889606000	-2.162147000
6	3.002745000	0.976858000	1.344718000
1	3.178332000	1.542724000	2.268977000
1	3.953971000	0.467982000	1.134956000
6	1.863793000	-0.044927000	1.541838000
6	1.322121000	-0.036572000	2.944128000
9	0.060028000	-0.612345000	2.989809000
9	2.058154000	-0.717085000	3.860882000
9	1.163595000	1.212106000	3.437521000
6	2.083806000	-1.403551000	0.952783000
6	2.802133000	-1.523300000	-0.265951000
6	1.580968000	-2.602269000	1.510092000
6	3.000899000	-2.748081000	-0.878358000
6	1.786448000	-3.833932000	0.890991000
6	2.495122000	-3.934587000	-0.313443000
1	1.041125000	-2.584457000	2.447850000
1	3.551465000	-2.819529000	-1.811159000
1	1.389646000	-4.725058000	1.369042000
1	3.180592000	-0.625318000	-0.742447000
6	2.736922000	-5.225651000	-1.018782000
8	3.333484000	-5.257601000	-2.087598000
6	2.228416000	-6.514840000	-0.387380000
1	2.666752000	-6.668476000	0.606062000
1	2.499598000	-7.350557000	-1.034958000
1	1.138945000	-6.493148000	-0.262346000
6	3.599236000	2.951940000	-0.194612000
6	4.917304000	2.590560000	-0.530173000
6	3.262892000	4.316562000	-0.240575000
6	5.852776000	3.550147000	-0.915943000
6	4.197602000	5.278929000	-0.624682000
6	5.496907000	4.899864000	-0.966471000
1	5.209472000	1.544088000	-0.502126000
1	2.260774000	4.621800000	0.047267000
1	6.861922000	3.242681000	-1.178665000
1	3.911847000	6.327688000	-0.647808000
1	6.226754000	5.648386000	-1.263413000

94

Bb SCF Done: E(RB3LYP) = -2028.7363072

6	0.694771000	-2.464593000	-0.894743000
1	-0.384007000	-2.305640000	-1.043164000
6	0.867121000	-3.887532000	-0.322221000
1	1.932438000	-4.101927000	-0.167957000
1	0.382071000	-3.983595000	0.654936000
6	0.272007000	-4.935812000	-1.282414000
1	0.428865000	-5.941637000	-0.871901000
1	-0.816679000	-4.788642000	-1.340162000
6	0.872107000	-4.833095000	-2.692011000
1	1.936681000	-5.108310000	-2.651910000
1	0.388777000	-5.554495000	-3.362923000
6	0.740962000	-3.409287000	-3.251471000
1	-0.319339000	-3.169516000	-3.414319000
1	1.231304000	-3.337010000	-4.230472000
6	1.348101000	-2.372576000	-2.291359000
1	1.235560000	-1.365922000	-2.706638000
1	2.424429000	-2.567401000	-2.202155000
6	3.014383000	-0.832222000	0.440590000
1	3.080581000	-0.044209000	1.205772000
6	3.749946000	-2.077136000	0.975654000
1	3.264721000	-2.464104000	1.879502000
1	3.706213000	-2.878336000	0.226080000
6	5.226263000	-1.757737000	1.275219000
1	5.276638000	-1.028649000	2.097160000
1	5.736907000	-2.663085000	1.627914000
6	5.940850000	-1.184714000	0.042746000
1	6.976583000	-0.922001000	0.292848000
1	5.994397000	-1.960307000	-0.735959000

6	5.198513000	0.040602000	-0.511967000
1	5.691181000	0.403136000	-1.423418000
1	5.246038000	0.859620000	0.218954000
6	3.723417000	-0.276503000	-0.813845000
1	3.206328000	0.621397000	-1.168499000
1	3.680618000	-1.010618000	-1.626529000
6	0.510098000	-1.531742000	1.929920000
1	0.925736000	-2.530092000	2.123349000
6	-1.031293000	-1.638151000	1.918871000
1	-1.456589000	-0.656738000	1.668247000
1	-1.369378000	-2.329909000	1.137911000
6	-1.581116000	-2.095119000	3.281196000
1	-1.248046000	-3.124403000	3.482338000
1	-2.677092000	-2.120785000	3.242403000
6	-1.109050000	-1.176634000	4.416230000
1	-1.547225000	-0.177736000	4.277413000
1	-1.473156000	-1.546454000	5.382942000
6	0.421364000	-1.062839000	4.430212000
1	0.856610000	-2.041295000	4.682954000
1	0.747193000	-0.365720000	5.212845000
6	0.972731000	-0.600840000	3.069329000
1	2.066861000	-0.557891000	3.117897000
1	0.630751000	0.421010000	2.865111000
15	1.137012000	-0.978403000	0.225847000
28	-0.094715000	0.836119000	-0.727385000
6	0.369058000	2.639122000	-0.401247000
6	-0.386478000	3.601227000	-0.971789000
6	-0.190198000	5.090470000	-0.820102000
1	-1.088103000	5.563865000	-0.397272000
1	0.658033000	5.333331000	-0.175370000
1	-0.022481000	5.563140000	-1.798603000
6	-1.515411000	3.124951000	-1.840016000
1	-1.352499000	3.453488000	-2.875896000
1	-2.472751000	3.578993000	-1.543425000
6	-1.581081000	1.583770000	-1.760716000
6	-1.409642000	0.950849000	-3.115230000
9	-1.087485000	-0.388974000	-3.013736000
9	-2.510306000	0.997863000	-3.912078000
9	-0.404272000	1.510640000	-3.826734000
6	-2.686236000	1.015996000	-0.925786000
6	-3.092478000	1.703582000	0.248195000
6	-3.342829000	-0.205706000	-1.200811000
6	-4.081038000	1.207221000	1.079419000
6	-4.340714000	-0.699789000	-0.362634000
6	-4.729239000	-0.009270000	0.792660000
1	-3.094750000	-0.770673000	-2.089749000
1	-4.378054000	1.746803000	1.973242000
1	-4.822263000	-1.636183000	-0.629954000
1	-2.597046000	2.632100000	0.512048000
6	-5.788126000	-0.495528000	1.723542000
8	-6.068528000	0.128557000	2.738862000
6	-6.522804000	-1.786940000	1.390516000
1	-7.034598000	-1.711654000	0.423530000
1	-7.257651000	-1.982034000	2.173600000
1	-5.828771000	-2.633737000	1.326617000
6	1.534759000	2.895620000	0.469252000
6	1.404374000	2.903640000	1.871831000
6	2.805275000	3.171197000	-0.073936000
6	2.496189000	3.172823000	2.697539000
6	3.896984000	3.446757000	0.750825000
6	3.749036000	3.443054000	2.140754000
1	0.425399000	2.719480000	2.306189000
1	2.920937000	3.186036000	-1.154511000
1	2.366575000	3.183371000	3.776992000
1	4.863293000	3.673670000	0.307175000
1	4.598918000	3.659076000	2.782725000

94

TSBb-Cb SCF Done: E(RB3LYP) = -2028.7122751

6	-3.040022000	-1.046889000	1.091583000
1	-2.399540000	-1.823805000	1.525683000
6	-4.394942000	-1.680256000	0.720365000
1	-5.067413000	-0.909963000	0.320514000
1	-4.277482000	-2.436114000	-0.065831000
6	-5.054182000	-2.312300000	1.960166000
1	-6.026388000	-2.741664000	1.685449000
1	-4.430463000	-3.146399000	2.313359000
6	-5.221690000	-1.286442000	3.090849000
1	-5.942556000	-0.517381000	2.774844000
1	-5.649648000	-1.767959000	3.979282000
6	-3.884767000	-0.615533000	3.439688000
1	-3.202021000	-1.362109000	3.869791000
1	-4.033593000	0.153140000	4.208828000
6	-3.216011000	0.011307000	2.202924000
1	-2.241264000	0.421998000	2.481430000
1	-3.833638000	0.844828000	1.845049000
6	-2.903061000	0.677588000	-1.507246000
1	-2.121792000	0.983082000	-2.213609000
6	-4.044310000	0.064093000	-2.347965000
1	-3.697942000	-0.821893000	-2.891480000
1	-4.863484000	-0.264569000	-1.697962000
6	-4.585407000	1.096568000	-3.355789000
1	-3.794457000	1.338411000	-4.080812000
1	-5.409911000	0.653728000	-3.928914000
6	-5.048155000	2.386013000	-2.662024000
1	-5.385870000	3.117164000	-3.407184000
1	-5.918046000	2.162630000	-2.026529000
6	-3.930579000	2.984560000	-1.795239000
1	-4.295395000	3.869764000	-1.259059000
1	-3.109802000	3.326812000	-2.442458000
6	-3.387483000	1.955779000	-0.788781000
1	-2.575067000	2.393008000	-0.195904000
1	-4.188837000	1.696070000	-0.085193000
6	-1.653785000	-2.035697000	-1.369178000

1	-2.625192000	-2.341074000	-1.783221000
6	-1.111682000	-3.206367000	-0.519665000
1	-0.196082000	-2.891465000	-0.005319000
1	-1.828986000	-3.474793000	0.263486000
6	-0.821130000	-4.445224000	-1.385370000
1	-1.766266000	-4.832274000	-1.794728000
1	-0.407028000	-5.241149000	-0.753266000
6	0.135558000	-4.124496000	-2.541508000
1	1.115544000	-3.836940000	-2.133194000
1	0.299531000	-5.016095000	-3.159804000
6	-0.411420000	-2.974235000	-3.396590000
1	-1.339051000	-3.297546000	-3.892157000
1	0.296581000	-2.717047000	-4.194366000
6	-0.695814000	-1.727522000	-2.542090000
1	-1.105522000	-0.933775000	-3.179030000
1	0.250787000	-1.346973000	-2.134085000
15	-1.943372000	-0.466885000	-0.339369000
28	0.035483000	0.165360000	0.640313000
6	0.614001000	1.708236000	-0.328792000
6	1.458151000	2.470095000	0.414693000
6	0.243963000	2.071842000	-1.746759000
1	-0.754025000	2.521171000	-1.824137000
1	0.949473000	2.789289000	-2.183104000
1	0.243057000	1.182100000	-2.387775000
6	1.925845000	1.908303000	1.746801000
1	1.350508000	2.347305000	2.570570000
1	2.969267000	2.184227000	1.953164000
6	1.789814000	0.379281000	1.769887000
6	1.130307000	-0.190301000	2.868335000
9	-0.271985000	-1.174490000	2.019718000
9	1.540277000	-1.261144000	3.513338000
9	0.378618000	0.543012000	3.664313000
6	2.779378000	-0.468798000	1.041191000
6	3.862739000	0.112625000	0.350410000
6	2.658654000	-1.875556000	0.981179000
6	4.785610000	-0.665829000	-0.334654000
6	3.587245000	-2.651924000	0.297762000
6	4.672701000	-2.063917000	-0.371068000
1	1.817947000	-2.359527000	1.462179000
1	5.617942000	-0.205178000	-0.857260000
1	3.454045000	-3.729901000	0.287002000
1	3.978288000	1.189105000	0.332749000
6	5.700267000	-2.847711000	-1.117162000
8	6.625103000	-2.288747000	-1.691666000
6	5.581731000	-4.365263000	-1.152696000
1	5.613139000	-4.788322000	-0.141374000
1	6.411432000	-4.766330000	-1.737536000
1	4.633787000	-4.677585000	-1.607407000
6	1.942395000	3.833805000	0.046733000
6	3.300721000	4.191890000	0.143490000
6	1.040675000	4.828001000	-0.376461000
6	3.739322000	5.473577000	-0.188352000
6	1.474873000	6.111473000	-0.707719000
6	2.828347000	6.441080000	-0.617199000
1	4.031909000	3.456896000	0.468953000
1	-0.017375000	4.588319000	-0.428603000
1	4.796499000	5.715728000	-0.113463000
1	0.752228000	6.857784000	-1.028628000
1	3.169086000	7.440917000	-0.872643000

94

TSBb'-Cb' SCF Done: E(RB3LYP) = -2028.7155574

6	-2.497720000	-0.337945000	1.496966000
1	-1.607852000	-0.354950000	2.136849000
6	-3.447356000	-1.456145000	1.978158000
1	-4.360278000	-1.476769000	1.369616000
1	-2.980576000	-2.442033000	1.878728000
6	-3.831080000	-1.240235000	3.455343000
1	-4.525149000	-2.029321000	3.772737000
1	-2.929882000	-1.346498000	4.076745000
6	-4.447846000	0.144786000	3.694171000
1	-5.411258000	0.208104000	3.166111000
1	-4.666784000	0.283569000	4.760589000
6	-3.518880000	1.256737000	3.187967000
1	-2.598284000	1.272177000	3.788075000
1	-3.993661000	2.238427000	3.313623000
6	-3.142514000	1.048584000	1.711360000
1	-2.454355000	1.837758000	1.394193000
1	-4.051102000	1.136460000	1.100627000
6	-2.959798000	-0.184170000	-1.583677000
1	-2.427311000	-0.535961000	-2.477751000
6	-4.276116000	-0.979614000	-1.467789000
1	-4.078235000	-2.049776000	-1.332578000
1	-4.828305000	-0.646564000	-0.579596000
6	-5.162398000	-0.765600000	-2.708254000
1	-4.655291000	-1.182337000	-3.590836000
1	-6.100786000	-1.323216000	-2.593421000
6	-5.449012000	0.724604000	-2.941402000
1	-6.046651000	0.859382000	-3.851790000
1	-6.056159000	1.110311000	-2.108803000
6	-4.146274000	1.532203000	-3.035872000
1	-4.367289000	2.601298000	-3.148662000
1	-3.601279000	1.229009000	-3.941670000
6	-3.245519000	1.318479000	-1.805120000
1	-2.302616000	1.864063000	-1.922652000
1	-3.745569000	1.739339000	-0.924693000
6	-1.308178000	-2.359388000	-0.327784000
1	-2.202653000	-2.877860000	0.042064000
6	-0.127826000	-2.694815000	0.611703000
1	0.756680000	-2.131693000	0.282217000
1	-0.340516000	-2.363323000	1.635643000
6	0.192800000	-4.199258000	0.605280000
1	-0.652752000	-4.753188000	1.039956000
1	1.057946000	-4.393591000	1.251987000

6	0.458255000	-4.711662000	-0.816912000
1	1.373140000	-4.241662000	-1.206355000
1	0.641467000	-5.793640000	-0.804310000
6	-0.715272000	-4.379625000	-1.748877000
1	-1.605184000	-4.939825000	-1.424076000
1	-0.495390000	-4.707327000	-2.772697000
6	-1.033774000	-2.873448000	-1.754247000
1	-1.897701000	-2.692921000	-2.403703000
1	-0.192320000	-2.327181000	-2.191707000
15	-1.690072000	-0.504498000	-0.220217000
28	0.120618000	0.923965000	-0.117800000
6	0.526519000	1.272065000	-1.970034000
6	0.984116000	2.534473000	-2.134493000
6	1.363508000	3.190738000	-3.441834000
1	2.425313000	3.479338000	-3.445420000
1	1.187769000	2.539161000	-4.300368000
1	0.791571000	4.117794000	-3.595866000
6	1.204178000	3.364468000	-0.882352000
1	0.322986000	3.974520000	-0.650753000
1	2.022496000	4.085854000	-1.034325000
6	1.533276000	2.442779000	0.300036000
6	0.886829000	2.680376000	1.518577000
9	-0.022603000	1.026104000	1.813918000
9	1.470505000	2.613377000	2.694564000
9	-0.168384000	3.468855000	1.587943000
6	2.852583000	1.745757000	0.349407000
6	3.869813000	2.046424000	-0.578655000
6	3.122452000	0.742325000	1.305829000
6	5.096359000	1.396046000	-0.539343000
6	4.352151000	0.095987000	1.343491000
6	5.365288000	0.411799000	0.423840000
1	2.347740000	0.455443000	2.007448000
1	5.875839000	1.640301000	-1.254117000
1	4.514103000	-0.669320000	2.096903000
1	3.703477000	2.797215000	-1.342586000
6	6.704306000	-0.248127000	0.419800000
8	7.551578000	0.050824000	-0.411154000
6	7.004214000	-1.304809000	1.474060000
1	6.917964000	-0.889858000	2.485567000
1	8.020244000	-1.672625000	1.320157000
1	6.301007000	-2.143649000	1.405111000
6	0.497600000	0.306082000	-3.095151000
6	1.517221000	-0.661679000	-3.193952000
6	-0.470476000	0.338243000	-4.115477000
6	1.562402000	-1.559603000	-4.260736000
6	-0.432207000	-0.566099000	-5.180131000
6	0.582010000	-1.522223000	-5.256567000
1	2.287649000	-0.688717000	-2.427585000
1	-1.246827000	1.097363000	-4.081541000
1	2.368721000	-2.286889000	-4.317879000
1	-1.191634000	-0.514952000	-5.956886000
1	0.615676000	-2.222088000	-6.087259000

94

Cb SCF Done: E(RB3LYP) = -2028.7213572

6	-2.248571000	2.149489000	-0.766474000
1	-1.344957000	2.743823000	-0.607742000
6	-3.474658000	2.970207000	-0.325049000
1	-4.393850000	2.395916000	-0.498098000
1	-3.436307000	3.191435000	0.748836000
6	-3.561990000	4.284675000	-1.123424000
1	-4.450910000	4.848893000	-0.812454000
1	-2.690971000	4.910698000	-0.881170000
6	-3.595264000	4.021323000	-2.635731000
1	-4.527468000	3.494399000	-2.889688000
1	-3.614829000	4.971026000	-3.185567000
6	-2.395060000	3.172371000	-3.078721000
1	-1.469200000	3.747145000	-2.933234000
1	-2.460484000	2.949640000	-4.151544000
6	-2.292251000	1.858469000	-2.282131000
1	-1.384834000	1.325288000	-2.575994000
1	-3.149611000	1.220200000	-2.531956000
6	-3.425992000	-0.379479000	0.584520000
1	-3.040002000	-1.287270000	1.059030000
6	-4.486075000	0.199658000	1.549003000
1	-4.035568000	0.465252000	2.511504000
1	-4.919714000	1.119012000	1.138990000
6	-5.613950000	-0.822615000	1.790213000
1	-5.202091000	-1.694884000	2.318878000
1	-6.369908000	-0.384275000	2.454430000
6	-6.260520000	-1.284863000	0.476724000
1	-7.028428000	-2.042568000	0.677512000
1	-6.776292000	-0.433353000	0.008108000
6	-5.209075000	-1.835368000	-0.497332000
1	-5.677380000	-2.107799000	-1.451719000
1	-4.776340000	-2.758709000	-0.085661000
6	-4.084750000	-0.814516000	-0.743387000
1	-3.333834000	-1.233014000	-1.424891000
1	-4.511162000	0.062399000	-1.247797000
6	-1.417104000	1.314585000	2.006346000
1	-2.351340000	1.721508000	2.417181000
6	-0.376235000	2.454648000	1.981959000
1	0.519451000	2.122039000	1.449684000
1	-0.766392000	3.304266000	1.410258000
6	-0.027631000	2.926405000	3.404124000
1	-0.912732000	3.391710000	3.864540000
1	0.740975000	3.707468000	3.347785000
6	0.450148000	1.770487000	4.292715000
1	1.395362000	1.373819000	3.894374000
1	0.662895000	2.129750000	5.307577000
6	-0.594816000	0.647859000	4.332929000
1	-1.505171000	1.016086000	4.829399000
1	-0.232124000	-0.196603000	4.933049000
6	-0.949182000	0.158343000	2.919002000

1	-1.719955000	-0.620282000	2.982650000
1	-0.067099000	-0.314267000	2.464507000
15	-1.838362000	0.629061000	0.287309000
28	0.091114000	-0.315539000	-0.569677000
6	-0.002940000	-2.210978000	-0.203078000
6	1.147697000	-2.889961000	-0.425432000
6	-1.201190000	-2.939933000	0.358011000
1	-1.040575000	-4.021405000	0.431279000
1	-1.455480000	-2.583395000	1.364779000
1	-2.085734000	-2.785779000	-0.270377000
6	2.343754000	-2.216479000	-1.068452000
1	2.654653000	-2.774289000	-1.964919000
1	3.208135000	-2.254157000	-0.396542000
6	2.083605000	-0.764194000	-1.448786000
6	1.208777000	-0.571977000	-2.487612000
9	0.451155000	1.473765000	-0.725385000
9	1.032424000	0.552535000	-3.168646000
9	0.680253000	-1.583057000	-3.173469000
6	2.971833000	0.301540000	-0.913726000
6	3.420712000	0.213717000	0.418779000
6	3.433504000	1.369661000	-1.699190000
6	4.285533000	1.160960000	0.946548000
6	4.308027000	2.314642000	-1.170455000
6	4.745875000	2.228279000	0.158918000
1	3.108548000	1.467485000	-2.727512000
1	4.623255000	1.097987000	1.975834000
1	4.648102000	3.124532000	-1.808512000
1	3.067104000	-0.596687000	1.049738000
6	5.679839000	3.220104000	0.779020000
8	6.036875000	3.098888000	1.942216000
6	6.178103000	4.385028000	-0.062431000
1	6.723941000	4.029774000	-0.944833000
1	6.841523000	4.998973000	0.549186000
1	5.342866000	4.998766000	-0.420761000
6	1.387114000	-4.330405000	-0.085149000
6	1.466037000	-4.761551000	1.249092000
6	1.584977000	-5.282634000	-1.099662000
6	1.723477000	-6.097968000	1.560283000
6	1.840843000	-6.619328000	-0.792249000
6	1.912427000	-7.032193000	0.540222000
1	1.322985000	-4.036083000	2.046060000
1	1.520715000	-4.972770000	-2.140137000
1	1.779264000	-6.408248000	2.600780000
1	1.981550000	-7.339877000	-1.594133000
1	2.113985000	-8.072720000	0.780776000

94

Cb' SCF Done: E(RB3LYP) = -2028.7265983

6	-0.934987000	2.523322000	-0.836567000
1	0.130477000	2.701774000	-0.673327000
6	-1.722916000	3.766155000	-0.378302000
1	-2.799188000	3.613539000	-0.528810000
1	-1.577636000	3.948068000	0.693992000
6	-1.285268000	5.010065000	-1.174704000
1	-1.875177000	5.879435000	-0.856199000
1	-0.235910000	5.237895000	-0.937045000
6	-1.426019000	4.790245000	-2.687729000
1	-2.491044000	4.672058000	-2.938561000
1	-1.073610000	5.674904000	-3.233547000
6	-0.655623000	3.541288000	-3.139789000
1	0.421266000	3.704820000	-2.989416000
1	-0.799998000	3.371807000	-4.214681000
6	-1.082004000	2.287125000	-2.355822000
1	-0.453327000	1.442593000	-2.650392000
1	-2.117370000	2.036303000	-2.616622000
6	-3.030475000	0.661465000	0.490638000
1	-3.065601000	-0.380824000	0.826946000
6	-3.700334000	1.520605000	1.587881000
1	-3.184099000	1.394327000	2.545375000
1	-3.637686000	2.586169000	1.333513000
6	-5.179760000	1.134762000	1.771160000
1	-5.236519000	0.102143000	2.144430000
1	-5.630324000	1.774249000	2.541441000
6	-5.963401000	1.240605000	0.456837000
1	-7.006470000	0.934980000	0.609147000
1	-5.989288000	2.291873000	0.132087000
6	-5.310329000	0.381050000	-0.633315000
1	-5.842784000	0.500805000	-1.586126000
1	-5.389291000	-0.677391000	-0.356705000
6	-3.827744000	0.741510000	-0.831391000
1	-3.381169000	0.076866000	-1.580531000
1	-3.771734000	1.759294000	-1.236079000
6	-0.491388000	1.413191000	1.921914000
1	-1.215167000	2.111771000	2.362069000
6	0.881984000	2.118203000	1.888859000
1	1.597098000	1.500073000	1.338667000
1	0.807561000	3.061316000	1.335640000
6	1.391366000	2.412411000	3.310857000
1	0.728169000	3.145755000	3.794848000
1	2.382071000	2.880477000	3.250727000
6	1.452289000	1.145514000	4.173684000
1	2.195881000	0.454846000	3.749929000
1	1.791766000	1.388898000	5.188522000
6	0.084025000	0.452736000	4.219968000
1	-0.635000000	1.101516000	4.742436000
1	0.141294000	-0.478207000	4.798990000
6	-0.438319000	0.148271000	2.806050000
1	-1.427562000	-0.321341000	2.867355000
1	0.222759000	-0.586471000	2.324703000
15	-1.170029000	0.956950000	0.205164000
28	0.228646000	-0.663276000	-0.647831000
6	-0.623436000	-2.386304000	-0.344457000
6	0.184761000	-3.455996000	-0.535450000
6	-0.174161000	-4.906401000	-0.284689000

1	-1.182753000	-5.028194000	0.112512000
1	-0.100184000	-5.489375000	-1.214709000
1	0.529057000	-5.366264000	0.425227000
6	1.588807000	-3.286392000	-1.070973000
1	1.727050000	-3.934640000	-1.951359000
1	2.320569000	-3.643647000	-0.336999000
6	1.938969000	-1.853466000	-1.448322000
6	1.272877000	-1.345867000	-2.532656000
9	1.270041000	0.834628000	-0.784996000
9	1.582914000	-0.245285000	-3.201469000
9	0.422536000	-2.070388000	-3.254981000
6	3.145397000	-1.214397000	-0.857437000
6	3.447372000	-1.440894000	0.499720000
6	4.037802000	-0.439076000	-1.614145000
6	4.587457000	-0.902954000	1.078552000
6	5.185738000	0.093267000	-1.033997000
6	5.477968000	-0.126773000	0.319643000
1	3.836580000	-0.246729000	-2.660801000
1	4.814849000	-1.069591000	2.126459000
1	5.855695000	0.684330000	-1.650808000
1	2.765278000	-2.027562000	1.108622000
6	6.694317000	0.427126000	0.994377000
8	6.910059000	0.200281000	2.176301000
6	7.658315000	1.279790000	0.183912000
1	8.066763000	0.716296000	-0.663704000
1	8.475815000	1.596963000	0.833686000
1	7.154503000	2.163985000	-0.224628000
6	-2.011664000	-2.649618000	0.117790000
6	-2.314836000	-2.818281000	1.482289000
6	-3.053229000	-2.836501000	-0.809664000
6	-3.602275000	-3.159382000	1.902955000
6	-4.338596000	-3.187305000	-0.392424000
6	-4.621891000	-3.348083000	0.966875000
1	-1.520053000	-2.704397000	2.214194000
1	-2.836881000	-2.731172000	-1.869904000
1	-3.804271000	-3.294434000	2.962926000
1	-5.119248000	-3.345916000	-1.132660000
1	-5.621425000	-3.625461000	1.290853000

94

TSCb-C1b SCF Done: E(RB3LYP) = -2028.6988642

6	-0.559297000	2.892861000	0.334457000
1	0.211528000	2.581974000	1.048413000
6	-1.290478000	4.131605000	0.885434000
1	-2.033786000	4.485630000	0.158786000
1	-1.837304000	3.896951000	1.806712000
6	-0.284740000	5.267879000	1.153615000
1	-0.814755000	6.152937000	1.529303000
1	0.406226000	4.953194000	1.948938000
6	0.517496000	5.621404000	-0.107995000
1	-0.162571000	6.056077000	-0.856340000
1	1.262465000	6.394052000	0.121348000
6	1.200297000	4.382114000	-0.706108000
1	1.972304000	4.017051000	-0.014284000
1	1.713455000	4.645018000	-1.640248000
6	0.192940000	3.247925000	-0.966614000
1	0.714509000	2.368722000	-1.358682000
1	-0.515732000	3.574796000	-1.739697000
6	-3.075439000	1.460746000	-0.842571000
1	-3.527203000	0.460031000	-0.778722000
6	-4.154547000	2.460833000	-0.375806000
1	-4.426525000	2.288170000	0.671607000
1	-3.763931000	3.483561000	-0.434702000
6	-5.415601000	2.353083000	-1.253961000
1	-5.870654000	1.361757000	-1.109935000
1	-6.160124000	3.088249000	-0.922427000
6	-5.094995000	2.552447000	-2.742645000
1	-6.000639000	2.418373000	-3.347615000
1	-4.761386000	3.588166000	-2.904565000
6	-3.992826000	1.590326000	-3.210293000
1	-3.731627000	1.789966000	-4.257369000
1	-4.369454000	0.557280000	-3.173300000
6	-2.736747000	1.702940000	-2.329551000
1	-1.967619000	0.999458000	-2.668461000
1	-2.312008000	2.707752000	-2.447323000
6	-2.210104000	0.978846000	1.967352000
1	-2.996875000	1.728611000	2.130299000
6	-1.149341000	1.148309000	3.078068000
1	-0.288123000	0.509113000	2.859896000
1	-0.765639000	2.174563000	3.076256000
6	-1.731229000	0.833948000	4.467030000
1	-2.496070000	1.583310000	4.722625000
1	-0.939662000	0.928214000	5.221290000
6	-2.361034000	-0.563977000	4.525745000
1	-1.577560000	-1.322173000	4.380471000
1	-2.796904000	-0.747331000	5.516166000
6	-3.428265000	-0.724989000	3.435601000
1	-4.264585000	-0.040087000	3.640686000
1	-3.845170000	-1.740421000	3.450660000
6	-2.854941000	-0.423872000	2.040688000
1	-3.650206000	-0.524669000	1.290255000
1	-2.090092000	-1.173381000	1.792722000
15	-1.512034000	1.262645000	0.225240000
28	0.153769000	-0.343777000	-0.229793000
6	-0.726454000	-1.885310000	-1.051452000
6	-0.120884000	-2.962040000	-0.480998000
6	-2.002065000	-1.892510000	-1.839794000
1	-2.320653000	-2.912875000	-2.082720000
1	-2.824369000	-1.397321000	-1.313672000
1	-1.857488000	-1.355312000	-2.784425000
6	1.372315000	-2.801131000	-0.195935000
1	1.942209000	-3.612110000	-0.676828000
1	1.594062000	-2.864287000	0.875551000
6	1.767661000	-1.444372000	-0.763200000

6	1.045357000	-1.174236000	-1.965574000
9	1.044953000	0.598012000	1.041542000
9	1.233263000	0.019206000	-2.586496000
9	0.873096000	-2.062224000	-2.978526000
6	3.077176000	-0.837083000	-0.440141000
6	3.529256000	-0.804739000	0.894741000
6	3.927211000	-0.326243000	-1.438114000
6	4.781782000	-0.301779000	1.210788000
6	5.181806000	0.184681000	-1.117524000
6	5.632025000	0.199992000	0.210956000
1	3.610202000	-0.334815000	-2.475433000
1	5.127999000	-0.274966000	2.238842000
1	5.812816000	0.562225000	-1.916534000
1	2.872398000	-1.148039000	1.684903000
6	6.972310000	0.726715000	0.611733000
8	7.331403000	0.707085000	1.780972000
6	7.891061000	1.291816000	-0.462489000
1	8.125065000	0.535127000	-1.221091000
1	8.816155000	1.625982000	0.010639000
1	7.421780000	2.137589000	-0.979313000
6	-0.774906000	-4.256289000	-0.184040000
6	-2.114101000	-4.307634000	0.250441000
6	-0.075077000	-5.473836000	-0.296217000
6	-2.733914000	-5.522302000	0.537219000
6	-0.697620000	-6.689069000	-0.016455000
6	-2.029830000	-6.720433000	0.400601000
1	-2.661740000	-3.380839000	0.388587000
1	0.959669000	-5.475890000	-0.623136000
1	-3.766229000	-5.531801000	0.877026000
1	-0.138235000	-7.614716000	-0.123031000
1	-2.511519000	-7.668157000	0.625050000

94

TSCb'-C1b' SCF Done: E(RB3LYP) = -2028.7009343

6	-0.331714000	2.562999000	-0.437515000
1	0.456356000	2.653636000	0.316703000
6	-1.123125000	3.883651000	-0.497503000
1	-1.915256000	3.812693000	-1.255305000
1	-1.620305000	4.087027000	0.459341000
6	-0.197297000	5.060161000	-0.858699000
1	-0.782949000	5.986366000	-0.926591000
1	0.529844000	5.206960000	-0.046754000
6	0.555227000	4.803638000	-2.172552000
1	-0.167548000	4.773046000	-3.002107000
1	1.239848000	5.634639000	-2.385726000
6	1.325020000	3.475478000	-2.122052000
1	2.121826000	3.544039000	-1.367720000
1	1.819040000	3.284928000	-3.083663000
6	0.403725000	2.294420000	-1.769306000
1	0.993365000	1.376791000	-1.687519000
1	-0.313820000	2.147607000	-2.586300000
6	-2.938884000	0.871833000	-0.633470000
1	-3.247145000	-0.143204000	-0.349933000
6	-4.068033000	1.831880000	-0.196480000
1	-4.227810000	1.775516000	0.885742000
1	-3.793129000	2.870432000	-0.420194000
6	-5.390059000	1.497638000	-0.913021000
1	-5.727639000	0.500303000	-0.595130000
1	-6.166797000	2.207194000	-0.599262000
6	-5.232189000	1.513267000	-2.439132000
1	-6.178513000	1.238505000	-2.922552000
1	-4.997438000	2.536177000	-2.770100000
6	-4.111389000	0.562687000	-2.880835000
1	-3.967651000	0.619361000	-3.967859000
1	-4.400625000	-0.471759000	-2.653475000
6	-2.784734000	0.882490000	-2.170815000
1	-2.014427000	0.165813000	-2.480268000
1	-2.444884000	1.873404000	-2.496625000
6	-1.703819000	1.539671000	2.017894000
1	-2.466048000	2.327863000	1.951479000
6	-0.506330000	2.101890000	2.815820000
1	0.323109000	1.389174000	2.773181000
1	-0.137993000	3.019276000	2.343322000
6	-0.895430000	2.415833000	4.270547000
1	-1.626706000	3.238440000	4.284088000
1	-0.012099000	2.776052000	4.813084000
6	-1.500209000	1.199458000	4.982950000
1	-0.736840000	0.411981000	5.069865000
1	-1.798271000	1.462629000	6.005978000
6	-2.702445000	0.654784000	4.200110000
1	-3.508604000	1.403580000	4.204711000
1	-3.107257000	-0.241925000	4.687602000
6	-2.322609000	0.326541000	2.746553000
1	-3.205327000	-0.038083000	2.206717000
1	-1.591097000	-0.493959000	2.739558000
15	-1.253777000	1.051727000	0.238435000
28	0.336141000	-0.664561000	0.248801000
6	-0.616649000	-2.399280000	0.168694000
6	0.069914000	-3.184565000	1.035474000
6	-0.473658000	-4.332269000	1.840408000
1	-1.511116000	-4.565999000	1.592714000
1	0.139130000	-5.229480000	1.676988000
1	-0.419124000	-4.110364000	2.915626000
6	1.573786000	-2.948120000	1.111374000
1	2.101102000	-3.907648000	0.969082000
1	1.880416000	-2.575646000	2.096797000
6	1.901353000	-1.940809000	0.016201000
6	1.062890000	-2.156044000	-1.118300000
9	1.366977000	0.666182000	0.929949000
9	1.146363000	-1.308107000	-2.171339000
9	0.836302000	-3.375757000	-1.675895000
6	3.227980000	-1.292198000	-0.062878000
6	3.826676000	-0.768001000	1.100894000
6	3.953521000	-1.222287000	-1.266514000

6	5.097877000	-0.216346000	1.062229000
6	5.226575000	-0.660697000	-1.304425000
6	5.821925000	-0.153178000	-0.140251000
1	3.525852000	-1.618194000	-2.181323000
1	5.556911000	0.188339000	1.958347000
1	5.757751000	-0.633143000	-2.251043000
1	3.270600000	-0.769518000	2.030724000
6	7.189359000	0.450278000	-0.120898000
8	7.679425000	0.863325000	0.921057000
6	7.969002000	0.543820000	-1.425037000
1	8.136726000	-0.450246000	-1.857144000
1	8.932187000	1.015464000	-1.222429000
1	7.424011000	1.135610000	-2.170221000
6	-1.996763000	-2.637877000	-0.301225000
6	-3.074412000	-2.691861000	0.603787000
6	-2.264729000	-2.867974000	-1.663072000
6	-4.368024000	-2.981961000	0.165779000
6	-3.557624000	-3.159544000	-2.097752000
6	-4.615932000	-3.220263000	-1.187123000
1	-2.892768000	-2.500135000	1.656814000
1	-1.449547000	-2.842990000	-2.377998000
1	-5.181515000	-3.023099000	0.885644000
1	-3.736692000	-3.347007000	-3.153288000
1	-5.621270000	-3.450936000	-1.528580000

94

C1b SCF Done: E(RB3LYP) = -2028.7641247

6	1.332613000	-2.540860000	-0.902789000
1	0.339099000	-2.896563000	-0.603137000
6	2.361624000	-3.663250000	-0.666964000
1	3.364160000	-3.316834000	-0.954737000
1	2.414573000	-3.929123000	0.396021000
6	2.010564000	-4.912804000	-1.495616000
1	2.771879000	-5.688275000	-1.340004000
1	1.059932000	-5.328036000	-1.130643000
6	1.879418000	-4.582709000	-2.989824000
1	2.862511000	-4.279208000	-3.380746000
1	1.584537000	-5.478560000	-3.550966000
6	0.868925000	-3.449962000	-3.224415000
1	-0.136107000	-3.792770000	-2.939739000
1	0.822592000	-3.194872000	-4.290955000
6	1.218673000	-2.195489000	-2.404689000
1	0.447793000	-1.430102000	-2.551126000
1	2.164314000	-1.779631000	-2.776124000
6	3.250292000	-0.332698000	0.218817000
1	3.101616000	0.665408000	0.657481000
6	4.296747000	-1.061731000	1.089391000
1	3.935893000	-1.189269000	2.115116000
1	4.479281000	-2.068938000	0.691353000
6	5.622869000	-0.277462000	1.125104000
1	5.454183000	0.681435000	1.636836000
1	6.359324000	-0.826642000	1.725790000
6	6.175156000	-0.008133000	-0.281724000
1	7.086839000	0.599763000	-0.222003000
1	6.465519000	-0.962313000	-0.746165000
6	5.127378000	0.682733000	-1.165889000
1	5.513220000	0.809598000	-2.185754000
1	4.923211000	1.691090000	-0.777389000
6	3.815130000	-0.119025000	-1.201961000
1	3.081292000	0.386605000	-1.837887000
1	4.011440000	-1.094233000	-1.667186000
6	1.232065000	-1.679466000	1.960860000
1	2.139314000	-2.250149000	2.200577000
6	0.022739000	-2.625933000	2.123348000
1	-0.882067000	-2.116367000	1.779926000
1	0.142463000	-3.503601000	1.478093000
6	-0.125023000	-3.097356000	3.580615000
1	0.739592000	-3.721412000	3.854565000
1	-1.011432000	-3.738555000	3.665394000
6	-0.223554000	-1.920460000	4.560848000
1	-1.148167000	-1.359540000	4.361188000
1	-0.292867000	-2.285574000	5.593443000
6	0.980157000	-0.982002000	4.406663000
1	1.896700000	-1.509109000	4.711355000
1	0.882102000	-0.115840000	5.074197000
6	1.129923000	-0.500846000	2.953906000
1	2.003142000	0.158756000	2.869445000
1	0.251079000	0.103459000	2.685623000
15	1.467805000	-1.010847000	0.195769000
28	-0.378334000	0.341638000	-0.380719000
6	-0.041664000	2.063845000	-1.717861000
6	0.086329000	2.504101000	-0.407709000
6	0.942214000	2.047045000	-2.846750000
1	0.778335000	2.924275000	-3.488146000
1	1.976823000	2.064249000	-2.501761000
1	0.786798000	1.161928000	-3.470417000
6	-1.302505000	2.534615000	0.245849000
1	-1.733081000	3.549324000	0.182492000
1	-1.283331000	2.256678000	1.304824000
6	-1.998452000	1.494788000	-0.630421000
6	-1.506368000	1.814493000	-2.004001000
9	-1.301358000	-1.169152000	-0.126899000
9	-1.675531000	0.807995000	-2.936233000
9	-2.086127000	2.930481000	-2.611168000
6	-3.345137000	0.992702000	-0.334292000
6	-4.039490000	1.423801000	0.815971000
6	-3.976977000	0.042169000	-1.162930000
6	-5.302855000	0.936561000	1.120458000
6	-5.243700000	-0.439474000	-0.858530000
6	-5.929217000	-0.004128000	0.287963000
1	-3.461307000	-0.320056000	-2.042600000
1	-5.835412000	1.276493000	2.002963000
1	-5.696936000	-1.171033000	-1.520773000
1	-3.587746000	2.161815000	1.472471000

6	-7.288467000	-0.495345000	0.661846000
8	-7.850453000	-0.090954000	1.671435000
6	-7.972330000	-1.515935000	-0.237659000
1	-8.104232000	-1.122655000	-1.252861000
1	-8.948933000	-1.755978000	0.186648000
1	-7.376314000	-2.432948000	-0.318916000
6	1.259629000	3.142779000	0.229909000
6	2.142247000	3.959205000	-0.500515000
6	1.479625000	3.004320000	1.612942000
6	3.221198000	4.587105000	0.123378000
6	2.560347000	3.626443000	2.235071000
6	3.439995000	4.417382000	1.491463000
1	1.965704000	4.134357000	-1.555863000
1	0.804318000	2.393145000	2.202175000
1	3.883404000	5.220289000	-0.460810000
1	2.711946000	3.498518000	3.303464000
1	4.279343000	4.907449000	1.976902000

94

C1b' SCF Done: E(RB3LYP) = -2028.7603506

6	-1.418293000	1.755621000	-1.747433000
1	-0.395187000	2.146774000	-1.732391000
6	-2.382619000	2.887978000	-2.148453000
1	-3.416563000	2.516649000	-2.166706000
1	-2.354882000	3.703206000	-1.414939000
6	-2.029729000	3.436638000	-3.543793000
1	-2.746097000	4.218481000	-3.828049000
1	-1.041321000	3.916679000	-3.499448000
6	-2.004725000	2.321817000	-4.599668000
1	-3.021755000	1.920137000	-4.725321000
1	-1.709018000	2.730673000	-5.574298000
6	-1.057292000	1.184799000	-4.188393000
1	-0.023918000	1.559510000	-4.170941000
1	-1.085994000	0.377331000	-4.931194000
6	-1.407773000	0.623941000	-2.798511000
1	-0.674888000	-0.136624000	-2.509249000
1	-2.386304000	0.130360000	-2.851293000
6	-3.352427000	0.724955000	0.465681000
1	-3.252348000	0.095203000	1.364134000
6	-4.267826000	1.909712000	0.848583000
1	-3.827324000	2.497754000	1.659996000
1	-4.376710000	2.586562000	-0.008675000
6	-5.659649000	1.421030000	1.293343000
1	-5.554867000	0.848606000	2.227216000
1	-6.293932000	2.285279000	1.528457000
6	-6.329207000	0.536818000	0.232769000
1	-7.292697000	0.163086000	0.601740000
1	-6.548529000	1.141460000	-0.659519000
6	-5.416084000	-0.631335000	-0.162859000
1	-5.878537000	-1.226585000	-0.960520000
1	-5.293318000	-1.303386000	0.700093000
6	-4.035584000	-0.132746000	-0.622805000
1	-3.404921000	-0.980986000	-0.907751000
1	-4.169399000	0.474029000	-1.526795000
6	-1.147113000	2.647483000	1.098433000
1	-2.010747000	3.320284000	1.014882000
6	0.103243000	3.432353000	0.643699000
1	0.962244000	2.757152000	0.587192000
1	-0.044393000	3.816630000	-0.371945000
6	0.393978000	4.615755000	1.584109000
1	-0.419504000	5.353422000	1.507251000
1	1.308213000	5.124360000	1.253482000
6	0.530132000	4.172870000	3.046875000
1	1.410687000	3.521650000	3.148784000
1	0.704027000	5.040621000	3.695714000
6	-0.719677000	3.408832000	3.503287000
1	-1.584079000	4.089442000	3.503364000
1	-0.601357000	3.053834000	4.535361000
6	-1.011105000	2.217230000	2.575904000
1	-1.917457000	1.696881000	2.915072000
1	-0.185780000	1.494697000	2.653950000
15	-1.530481000	1.118713000	0.029629000
28	0.314376000	-0.367212000	0.313025000
6	0.015813000	-2.573335000	0.772972000
6	0.085469000	-1.863776000	1.963076000
6	-0.911993000	-1.820758000	3.083983000
1	-1.785275000	-2.449492000	2.900359000
1	-0.425432000	-2.174527000	4.002756000
1	-1.252109000	-0.798259000	3.283687000
6	1.519415000	-1.394688000	2.195180000
1	2.061146000	-2.122082000	2.827655000
1	1.570076000	-0.424554000	2.703777000
6	1.991230000	-1.344727000	0.744341000
6	1.403172000	-2.582814000	0.148458000
9	1.155974000	0.895392000	-0.638942000
9	1.354464000	-2.592980000	-1.229987000
9	2.082147000	-3.767521000	0.463858000
6	3.310334000	-0.817326000	0.369645000
6	4.182872000	-0.302332000	1.350821000
6	3.740493000	-0.782472000	-0.972588000
6	5.425799000	0.213861000	1.011384000
6	4.987058000	-0.270137000	-1.309291000
6	5.851811000	0.238224000	-0.325554000
1	3.084643000	-1.152431000	-1.749944000
1	6.096312000	0.602441000	1.771230000
1	5.281745000	-0.260604000	-2.354449000
1	3.887557000	-0.318221000	2.395854000
6	7.198832000	0.803615000	-0.634507000
8	7.920993000	1.234901000	0.254816000
6	7.666072000	0.836926000	-2.083001000
1	7.701722000	-0.172460000	-2.510370000
1	8.663289000	1.279359000	-2.117706000
1	6.983955000	1.429560000	-2.704449000
6	-1.064638000	-3.443712000	0.265735000

6	-2.424929000	-3.144695000	0.453297000
6	-0.731478000	-4.638696000	-0.401571000
6	-3.419295000	-4.012525000	0.003621000
6	-1.727725000	-5.503799000	-0.849471000
6	-3.074978000	-5.197610000	-0.648628000
1	-2.706382000	-2.214509000	0.932519000
1	0.309975000	-4.895284000	-0.550497000
1	-4.464066000	-3.756166000	0.156308000
1	-1.447173000	-6.422565000	-1.357116000
1	-3.849382000	-5.873208000	-1.001306000
